# Designing molecular qubits: computational insights into first-row and group 6 transition metal complexes†

**DOI:** 10.1039/d5sc02544c

**Published:** 2025-06-13

**Authors:** Arturo Sauza-de la Vega, Andrea Darù, Stephanie Nofz, Laura Gagliardi

**Affiliations:** a Department of Chemistry, The University of Chicago Chicago Illinois 60637 USA lgagliardi@uchicago.edu; b Pritzker School of Molecular Engineering, The University of Chicago Illinois 60637 USA; c James Franck Institute, The University of Chicago Illinois 60637 USA

## Abstract

In the realm of optically addressable qubits, a previously synthesized and characterized Cr(iv) pseudo-tetrahedral complex, featuring four strongly donating ligands surrounding the chromium center, has demonstrated potential as a qubit candidate. This study proposes analogs of this complex through a metal substitution strategy, extending the investigation to different complexes based on metal centers selected from first-row and Group 6 transition metals. Computational modeling based on multiconfigurational methods CASPT2 and MC-PDFT was utilized to calculate energy gaps between ground and excited electronic spin states, and zero-field splitting parameters. Simulations were applied to each equilibrium geometry and related deformations based on vibrational modes. All results align with previous experimental findings, but also show that qubits based on V and Ti centers could be more electronically stable than the Cr one, suggesting a lower electronic features dependency from their related geometry. In some cases geometrical deformations provide changes in relative energy gaps between triplet and singlet excited state, that could potentially swap, offering a different initialization process, and some inspiration for ligand design based on such deformations. Additionally, this study identifies an unsynthesized Ti(ii) compound as a promising candidate for molecular qubits. This finding highlights the role of computational multireference methods in the rational design of qubit systems.

## Abstract 2

Metal-substituted Cr(iv) qubits studied *via* CASPT2/MC-PDFT reveal Ti(ii) as a qubit candidate. Considering geometrical deformations show changes in relative energy gaps between excited states, this can provide some inspiration for ligand design.

## Graphical abstract

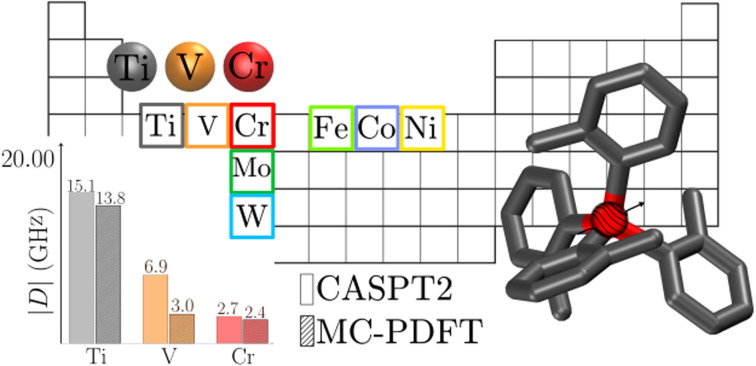

## 1 Introduction

Quantum bits (qubits) are the fundamental building blocks of quantum information science and technology. Their development is expected to significantly enhance transformative applications, including secure communication,^1,2^ complex financial modeling,^3,4^ and efficient drug design.^5,6^ Spin-based qubits have garnered significant attention due to their potential for long coherence times and compatibility with established experimental techniques. Among these, nitrogen-vacancy centers (NVs) in diamond have been extensively studied and are well-recognized for their applications in molecular sensing and quantum sensing technologies.^7–12^ While NV centers remain the most advanced systems demonstrating these properties, transition metal complexes with metal centers like chromium and vanadium also show significant promise as alternatives. This is because of the difficulty in selectively addressing disordered defect centers. In contrast, molecular qubits offer structural reproducibility and modularity. Molecular systems with a triplet ground state and a singlet excited state have demonstrated the ability of mimicking the mechanisms of the NV centers for optical initialization and readout mechanisms through spin polarization.^13^ The optical transitions in these materials typically fall within the visible to near-infrared spectrum, corresponding to energy gaps suitable for efficient optical manipulation. Furthermore, they present unique advantages such as tunable physical and chemical properties enabled by chemical synthesis, as well as scalability in production.^14–16^ Various platforms have been explored for molecular qubits, including organic radicals,^17^ rare-earth complexes,^18,19^ transition metal complexes,^14,20–24^ and hybrid systems featuring metal ions coordinated with organic radicals.^25,26^

Designing optically addressable molecular qubits requires specific spin properties and controlled zero-field splitting (ZFS). A non-singlet spin state, often a triplet analogous to NV centers, can be achieved by incorporating an appropriate metal ion into symmetric environments such as octahedral or tetrahedral coordination. For instance, d^2^ tetrahedral complexes^14,15^ have demonstrated favorable magnetic anisotropy, crucial for stabilizing specific spin states. Additionally, higher spin multiplicities in organic systems, such as quartet states, can also be viable qubits through intersystem crossing with a chromophore.^17^

Another key requirement for coherent microwave control is a small ZFS parameter (|*D*|), enabling precise spin-state manipulation. For optically addressable molecular qubits, a |*D*| value below 20 GHz is preferred,^15,27^ as it ensures that the spin polarization values fall within the X-band frequency range commonly utilized in electron paramagnetic resonance (EPR) spectroscopy. Triplet states have been widely studied due to their simplicity featuring only two unpaired electrons and three *M*_S_ levels (0 and ±1). Finally, an essential criterion is that the spin-lattice relaxation time (*T*_1_) must exceed the optical emission lifetime (*τ*_opt_) to maintain coherence.

In this study, we explore transition metal systems with a pseudo-tetrahedral (*S*_4_) symmetry and a strong ligand field to achieve the desired electronic structure.^15,28–30^ This structural choice optimizes microwave addressability by tuning the orbital configuration to yield a small |*D*| value, ensuring efficient spin control.^31–35^

When starting from existing qubit structures, these features are addressed after structural modifications. These modifications involve metal or ligand substitution, and solvent or host matrix modification.^15^ ZFS changes upon ligand substitution were previously experimentally studied by Freedman and collaborators,^32^ and computationally by^36,37^ using Cr(iv) as the metal center.^30^

One challenge in performing electronic structure calculations on such systems is selecting methodologies that can accurately capture their complex electronic structure. Multireference methods and Kohn–Sham density functional theory (KS-DFT) are usually the methods of choice.^38–40^ The zero-field splitting parameters can be computed using a coupled-perturbed response formalism within the framework of density functional theory.^41,42^ In systems containing transition metals, excited states lie close in energy, and multireference methods are generally preferred.^43^ While multiconfiguration perturbation theory methods like CASPT2 and NEVPT2 (ref. 44–46) provide improved accuracy over KS-DFT, their high computational cost limits their applicability to large molecular systems. Multiconfiguration pair-density functional theory^47–51^ (MC-PDFT) provides results of PT2 quality at a significantly reduced cost because it does not require the higher order density matrices used in PT2-based methods.

**Fig. 1:**
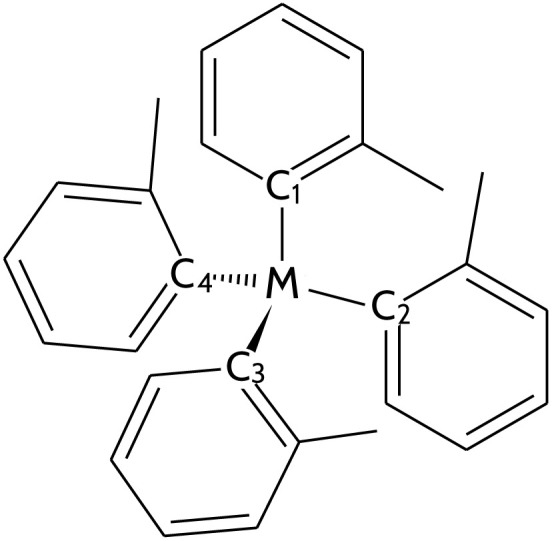
Molecular qubit structure with pseudo-tetrahedral ligand field. The M label indicates the considered metal centers, while the labels C_1_, C_2_, C_3_, and C_4_ the carbon atoms of each *o*-tolyl ligand bonded to the metal.

In this study, we computationally investigate the effect of metal substitution, starting from the experimentally made pseudo-tetrahedral Cr(iv) complex.^32,36^ We change the metal center while maintaining the same strong-field ligand environment formed by four *o*-tolyl ligands (Fig. 1). Seven complexes in addition to the Cr complex were considered; the metal centers were selected based on their similarity to Cr as a first-row transition metal and Group 6 elements. Specifically, we examined Ti, V, Fe, Co, Ni (first-row), and Mo and W (Group 6). The resulting complexes include titanium(ii) (Ti(*o*-tol)_4_^2−^), vanadium(iii) (V(*o*-tol)_4_^−^), chromium(iv) (Cr(*o*-tol)_4_), iron(ii) (Fe(*o*-tol)_4_^2−^), cobalt(ii) (Co(*o*-tol)_4_^2−^), nickel(ii) (Ni(*o*-tol)_4_^2−^), molybdenum(iv) (Mo(*o*-tol)_4_), and tungsten(iv) (W(*o*-tol)_4_). This selection was intended to explore the chemical space for metals that might offer ideal properties for molecular qubits, such as a small triplet–singlet gap, within the visible to near-infrared spectrum, and without any other triplet state in between, and axial ZFS parameters |*D*| smaller than 20 GHz (EPR X-band limit).^27^ Furthermore, given the potential toxicity of Cr, substituting it with less toxic metals such as Ti or Fe could present an advantage for biological or medical applications of molecular qubits. Multireference computational methods, namely CASPT2 and MC-PDFT, were used to assess the electronic and magnetic properties of these complexes, facilitating the identification of promising candidates for experimental validation. By systematically analyzing the impact of metal substitution on the electronic and magnetic properties of these compounds, we aim to provide valuable insights into the design principles of molecular qubits. Our major finding is that the Ti-based species investigated here for the first time has the requirements needed for a potential effective qubit (if it can be synthesized and stabilized). Furthermore, we demonstrate that MC-PDFT provides a quantitatively accurate description of these systems, making it a promising method for future studies due to its computational advantages over CASPT2.

## 2 Results and discussion

The electronic configuration, formal charge of the metal ions, and spin multiplicity of the complexes are reported in Table 1.

**Table 1:** Electronic configuration (El. Conf.) and formal charge of the metal ion, and spin considered for each compound

Complex	El. Conf.	Charge	Spin
Ti(*o*-tol)_4_^2−^	3d^2^	II	Triplet
V(*o*-tol)_4_^−^	3d^2^	III	Triplet
Cr(*o*-tol)_4_	3d^2^	IV	Triplet
Fe(*o*-tol)_4_^2−^	3d^6^	II	Quintet
Co(*o*-tol)_4_^2−^	3d^7^	II	Quartet
Ni(*o*-tol)_4_^2−^	3d^8^	II	Triplet^a^
Mo(*o*-tol)_4_	4d^2^	IV	Triplet
W(*o*-tol)_4_	5d^2^	IV	Triplet

a Notice that the ground state of Ni(*o*-tol)_4_^2−^ is a singlet state, but for comparative purposes the triplet has been considered in this work.

The molecular geometries of all species at any accessible spin state were optimized using density functional theory (DFT) with four functionals: PBE-D3BJ, B3LYP-D3BJ, TPSSh-D3BJ, and M06 (see Computational details). Upon optimization, these complexes adopt a pseudo-tetrahedral structure in all accessible spin states (beside the Ni singlet state, see below), which is an optimal geometry for optically addressable molecular qubits.^30,32^ The adiabatic energy gaps between spin states were evaluated with all the listed functionals. The geometric deviations among the selected functionals were minor, therefore the TPSSh-D3BJ optimized structures were selected for further multireference calculations due to its accuracy with most transition metal complexes.^52–54^ Vertical triplet–singlet gaps were calculated with *ab initio* multireference methods (MR) state-specific complete active space followed by second-order perturbation theory (CASPT2),^55,56^ and multiconfigurational pair-density functional theory (MC-PDFT)^47,57^ with the tPBE and tPBE0 functionals.

In the following, such complexes are discussed in two separate sections: first the complexes with electronic configuration d^2^, which have a triplet ground state (Cr(*o*-tol)_4_, V(*o*-tol)_4_^−^, Mo(*o*-tol)_4_, W(*o*-tol)_4_, Ti(*o*-tol)_4_^2−^); and subsequently those with a different ground-state spin multiplicity (Fe(*o*-tol)_4_^2−^, Co(*o*-tol)_4_^2−^, Ni(*o*-tol)_4_^2−^).

For the MR calculations, we explored active spaces of increasing size (see Computational details). In the following, we present results with the largest active space for each system, while we discuss the effect of increasing the active space in the Active Space Dependency section in the ESI.†

### 2.1 Triplet ground state: Ti, Cr, V, Mo, and W systems

In this section, we discuss the Cr(*o*-tol)_4_, Mo(*o*-tol)_4_, W(*o*-tol)_4_, Ti(*o*-tol)_4_^2−^, and V(*o*-tol)_4_^−^ complexes, which exhibit a d^2^ electronic configuration and a triplet ground state. The Cr, Mo, and V complexes have been previously investigated.^31^ The triplet–singlet energy gap of Cr(*o*-tol)_4_ has been experimentally determined as 1.209 eV (1025 nm).^30^ For Mo(*o*-tol)_4_ and V(*o*-tol)_4_^−^, the triplet–singlet gaps have been computationally estimated using periodic boundary condition density functional theory (PBC-DFT) with the PBE functional, yielding values of 0.69 eV (1800 nm) and 0.80 eV (1550 nm), respectively.^31,58^ In Table 2 we report the triplet–singlet gaps for these species.

**Table 2:** Triplet–singlet gaps (Δ*E*_TS_) for the Cr(*o*-tol)_4_, V(*o*-tol)_4_^−^, Mo(*o*-tol)_4_, W(*o*-tol)_4_, and Ti(*o*-tol)_4_^2−^ complexes. The DFT values correspond to adiabatic energy differences. The multireference (MR) values correspond to vertical energy differences, computed at the TPSSh triplet optimized geometries. All values are in eV. All the species have a d^2^ electronic configuration and a triplet ground state

Complex^a^	MR vertical Δ*E*_TS_			DFT^b^ adiabatic Δ*E*_TS_				Ref.
	CASPT2	tPBE	tPBE0	PBE	B3LYP	TPSSh	M06	
Cr(*o*-tol)_4_	1.44	1.04	1.20	0.51	1.52	1.55	1.67	1.209^c^
V(*o*-tol)_4_^−^	1.18	0.65	0.85	0.92	1.42	1.36	1.37	0.80^d^
Mo(*o*-tol)_4_	0.82	0.54	0.67	0.87	1.03	1.07	1.01	0.69^d^
W(*o*-tol)_4_	0.65	0.43	0.55	0.74	0.89	0.91	0.87	—
Ti(*o*-tol)_4_^2−^	0.56	0.49	0.55	0.20	0.43	0.41	0.44	—

a For Cr(*o*-tol)_4_, Mo(*o*-tol)_4_, W(*o*-tol)_4_, and Ti(*o*-tol)_4_^2−^, the data corresponds to the (10,15) active space, and for V(*o*-tol)_4_^−^, the (8,13) active space.

b All DFT functionals except M06 include D3BJ empirical correction. The RMSD values between optimized geometries is lower than 0.4 Å, thus DFT adiabatic triplet–singlet differences can be compared with MR vertical differences.

c Experimental value from ref. 30.

d These values are estimations based on periodic PBE diabatic calculations from ref. 31 corrected using the experimental values from ref. 30.

The multireference values are vertical differences computed at the ground-state triplet geometry, while the DFT ones are adiabatic. The geometrical differences between the optimized singlet and the triplet structures are minimal, (root mean square deviation values are lower than 0.4 Å see ESI†) therefore comparing DFT adiabatic values with vertical MR values is justified. Geometric parameters values are reported in the ESI in Tables S1 and S2.†

Our calculations predict a triplet ground state for all species. Table 2 shows that, among the multireference methods, the tPBE0 relative energies closely align with reference values, namely the experimental value for the Cr(*o*-tol)_4_ complex, and computational values for Mo(*o*-tol)_4_, and V(*o*-tol)_4_^−^,^31^ the values obtained are 1.20, 0.85, and 0.67 eV (27.7, 19.6, 15.5 kcal mol^−1^) respectively. In contrast, CASPT2 and tPBE exhibit a slightly larger discrepancy of approximately 0.2 eV in comparison to tPBE0 and the reference values (Table 2). The triplet–singlet energy gaps calculated, for the same list of complexes, are 1.44, 1.18, 0.82 eV (33.2, 27.2, 18.9 kcal mol^−1^) with CASPT2, and 1.04, 0.65, 0.54 eV with tPBE. We also considered the W(*o*-tol)_4_ and Ti(*o*-tol)_4_^2−^ complexes, for which there are no experimental reference values; the triplet–singlet energy gap is 0.55 eV (12.7 kcal mol^−1^) for both complexes with tPBE0, 0.65, and 0.56 eV (15.0, 12.9 kcal mol^−1^) respectively with CASPT2, and 0.43, and 0.49 eV respectively with tPBE (Table 2).

Multireference calculations can offer insights that can help rationalize the experimental results on the V(*o*-tol)_4_^−^ complex, which exhibits no measurable emission in the 900–1700 nm range.^31^ It has been suggested^31^ that this lack of emission may be due to a low-lying S_1_ excited state (>1200 nm, ∼1 eV) whose relaxation overlaps spectrally with high-energy C–H stretching overtones. This overlap facilitates a non-radiative decay *via* multi-phonon mediated relaxation pathways. The CASPT2 and tPBE0 singlet–triplet energy gaps of 1.18 eV and 0.85 eV respectively, fall within this energy range, supporting the proposed explanation.

All the DFT functionals predict a triplet–singlet gap larger than the previously reported values^31^ by an average of 0.3 eV. All values are reported in Table 2 and follow the same trend as the MR methods, with the exception of PBE-D3BJ. Indeed, PBE for the Cr(*o*-tol)_4_ complex provides an energy gap of 0.51 eV, which is 0.7 eV lower than the experimental reference. The values obtained for the list of complexes Cr(*o*-tol)_4_, V(*o*-tol)_4_^−^, Mo(*o*-tol)_4_, W(*o*-tol)_4_, and Ti(*o*-tol)_4_^2−^ are, with PBE-D3BJ 0.51, 0.92, 0.87, 0.74, 0.20 eV, while with TPSSh-D3BJ 1.55, 1.36, 1.07 0.91, and 0.41 eV (35.7, 31.4, 24.7, 21.0, 9.5 kcal mol^−1^) respectively (B3LYP-D3BJ, and M06 values are very similar to the ones obtained with TPSSh-D3BJ therefore they are only reported in Table 2). In conclusion, CASPT2, tPBE0, and TPSSh-D3BJ agree better with the experimental value in the Cr(*o*-tol)_4_ case, these methods will be used for the rest of the study. The triplet–singlet gaps follow the trend Cr(*o*-tol)_4_ > V(*o*-tol)_4_^−^ > Mo(*o*-tol)_4_ > W(*o*-tol)_4_ > Ti(*o*-tol)_4_^2−^ with all these three methods (Table 2, and Fig. S2†).

### 2.2 Non-triplet ground state: Fe, Co, and Ni systems

The Fe(*o*-tol)_4_^2−^ and Co(*o*-tol)_4_^2−^ complexes were optimized in their quintet and quartet ground states, respectively, resulting in pseudo-tetrahedral structures. Instead, Ni(*o*-tol)_4_^2−^ has a singlet ground state and a planar structure, however its triplet-optimized geometry is pseudo-tetrahedral; thus for the sake of comparisons with the other complexes, this non-ground state geometry is used for further calculations (even though it is not the lowest energy state for the current ligand environment). The complexes discussed in this section do not have a triplet ground state, and moreover a direct spin-flip first optical transition is missing due to the presence of other states between the ground state and the first excited state (see ESI†). Therefore they are not ideal qubit candidates. However, a brief description is reported next.

For the Ni(*o*-tol)_4_^2−^ species, at the triplet optimized geometry, (Fig. S2†) the CASPT2 and tPBE0 triplet–singlet gaps are 1.10 eV and 0.91 eV (25.4, 21.0 kcal mol^−1^) respectively. Different ligand environments for Ni(ii) could eventually be explored to have a triplet ground state, for qubit design purposes.

The Fe(*o*-tol)_4_^2−^ complex has a quasi doubly-degenerate quintet ground state, and the first excited state is also a quintet state, positioned at about 0.7 eV (16.1 kcal mol^−1^), according to all multireference methods (Fig. S9 and S17†). The lowest triplet excited state lies 1.53 eV, and 1.02 eV higher in energy using the CASPT2 and tPBE0 methods (35.5, 23.5 kcal mol^−1^), respectively. Moreover, this triplet is higher than several quintet states; therefore the qubit initialization cannot take place since there are no close-lying states of other spin multiplicities (see ESI†).

For the Co(*o*-tol)_4_^2−^ complex, similarly to the Fe(*o*-tol)_4_^2−^ complex, both the ground state and the first excited state are quartet states (Fig. S10 and S18†). The first doublet state is located after several quartet excited states and similarly the qubit initialization cannot take place (see ESI† for full details).

To summarize, due to their electronic structures, the Fe(*o*-tol)_4_^2−^, Co(*o*-tol)_4_^2−^, and Ni(*o*-tol)_4_^2−^ complexes are not relevant as potential molecular qubits. In the following we will consider only the Cr(*o*-tol)_4_, V(*o*-tol)_4_^−^, Mo(*o*-tol)_4_, Ti(*o*-tol)_4_^2−^, and W(*o*-tol)_4_ complexes for the zero-field splitting discussion.

### 2.3 Zero-field splitting parameters of the Cr, V, Mo, and Ti complexes

Optically addressable molecular qubits are considered for practical use when the axial ZFS parameter |*D*| falls into the X-band range, thus below 20 GHz (0.67 cm^−1^).^15,27^ The parameter |*D*| reflects the splitting of the triplet ground state *M*_S_ levels, which directly impacts the qubit's initialization and control, and the |*E*| parameter indicates whether a molecular system is uniaxial^59^ (see ESI† for further details). The ZFS depends on relativistic effects and electronic state interactions, both of which are incorporated into our calculations (see Computational details section).

**Fig. 2:**
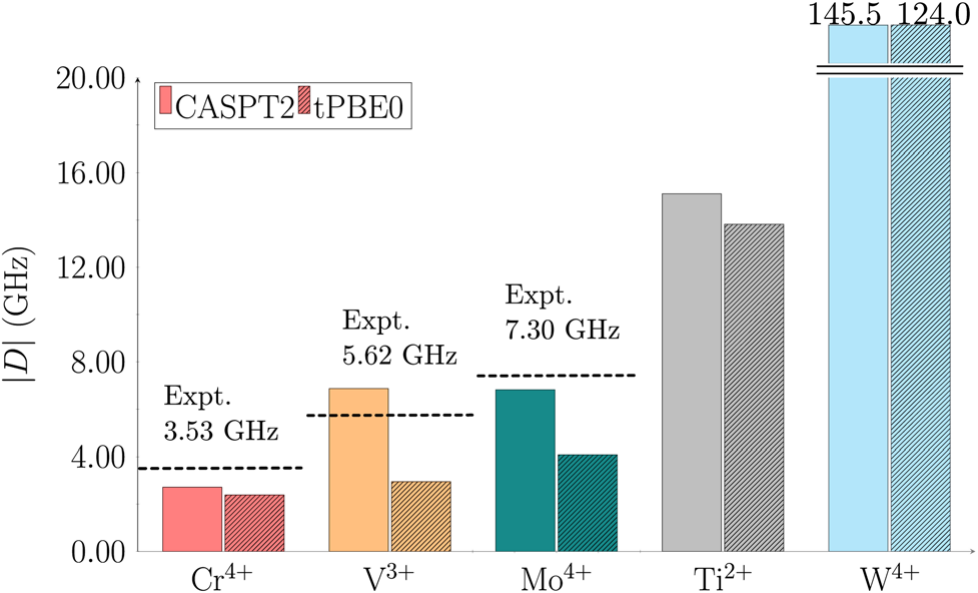
Calculated axial ZFS parameter |*D*| using CASPT2 (solid bars) and tPBE0 (striped bars) for the complexes: Cr(*o*-tol)_4_ red, V(*o*-tol)_4_^−^ orange, Mo(*o*-tol)_4_ teal, Ti(*o*-tol)_4_^2−^ gray, and W(*o*-tol)_4_ cyan. Active spaces of (8,13) for V(*o*-tol)_4_^−^ and (10,15) for Cr(*o*-tol)_4_, Mo(*o*-tol)_4_, Ti(*o*-tol)_4_^2−^, and W(*o*-tol)_4_ were used. Values are in GHz. Dashed lines indicate experimental data from ref. 30 and 31.

The experimentally reported |*D*| values for the Cr(*o*-tol)_4_, V(*o*-tol)_4_^−^, and Mo(*o*-tol)_4_ complexes are 3.53, 5.62, and 7.30 GHz, respectively.^30,31^ We computed ZFS parameters for these complexes as well as for the two unsynthesized species, Ti(*o*-tol)_4_^2−^ and W(*o*-tol)_4_, using multireference methods (see Computational details). The computed CASPT2 and tPBE0 |*D*| values are presented in Fig. 2.

The CASPT2 |*D*| values for the Cr(*o*-tol)_4_, V(*o*-tol)_4_^−^, Mo(*o*-tol)_4_, Ti(*o*-tol)_4_^2−^, and W(*o*-tol)_4_ complexes are 2.72, 6.87, 6.82, 15.11, and 145.50 GHz, respectively and the corresponding tPBE0 values are 2.39, 2.95, 4.09, 13.82, and 124.04 GHz. The W(*o*-tol)_4_ complex exceeds the optimal threshold for |*D*|, disqualifying it as a viable molecular qubit candidate. The tPBE0 method aligns closely with CASPT2 for Cr(*o*-tol)_4_, which exhibits the smallest |*D*| among the studied complexes. For the V(*o*-tol)_4_^−^ and Mo(*o*-tol)_4_ complexes, CASPT2 provides better agreement with experimental data than tPBE0, but both methods reproduce the experimental trend. Finally, we also calculated the rhombic parameter |*E*| which turned out to be 0 GHz in all cases (see ESI in Table S10†).

Although we reported the absolute |*D*| values thus far, the sign of *D* is also critical, but often challenging to determine experimentally. Computationally, we can resolve the sign: the computed axial parameters for Cr(*o*-tol)_4_, V(*o*-tol)_4_^−^, Mo(*o*-tol)_4_, and Ti(*o*-tol)_4_^2−^ are all negative. This agrees with recent literature,^60^ which reports *D* = −3.53 GHz for the Cr(*o*-tol)_4_ complex.

The observed increase in |*D*| values within the Group 6 complexes (Cr(*o*-tol)_4_, Mo(*o*-tol)_4_, W(*o*-tol)_4_) can be rationalized using eqn (S5) in the ESI,† which predicts a trend of increasing |*D*| with the spin–orbit coupling constant (*ζ*) and the atomic number (*Z*). Specifically, |*D*| scales proportionally to *ζ*^2^ and *Z*^4^. The spin–orbit coupling constants for Cr(*o*-tol)_4_, Mo(*o*-tol)_4_, and W(*o*-tol)_4_ are 325, 950, and 2300 cm^−1^, respectively,^61^ illustrating the substantial increase with heavier elements.

This relationship is visualized in Fig. S3,† where panel (a) shows the |*D*| parameters in cm^−1^ for the Cr(*o*-tol)_4_, Mo(*o*-tol)_4_, and W(*o*-tol)_4_ complexes, and panel (b) displays their corresponding squared spin–orbit coupling constants. These results confirm that relativistic effects significantly influence the |*D*| values in heavier metal centers.

The |*D*| values increase from Cr(*o*-tol)_4_ to V(*o*-tol)_4_^−^ and Mo(*o*-tol)_4_ to Ti(*o*-tol)_4_^2−^ (Fig. 2). This trend is explained considering the relative energies shown in Fig. S4–S8.† According to eqn (S5) in the ESI,† when the energy difference between two states is small, the contribution to *D* is large.

The axial parameters for the Cr(*o*-tol)_4_, V(*o*-tol)_4_^−^, Mo(*o*-tol)_4_, and Ti(*o*-tol)_4_^2−^ complexes are all below the 20 GHz threshold, supporting their potential as molecular qubits. Although this is consistent with previous findings for Cr(*o*-tol)_4_, V(*o*-tol)_4_^−^, and Mo(*o*-tol)_4_, the identification of Ti(*o*-tol)_4_^2−^ as a promising candidate is noteworthy. To the best of our knowledge, titanium has not previously been reported as a potential metal center for molecular qubit applications. However, Ti in the oxidation state II occurs in reactive species and catalytic processes. Notably, there are experimentally stable Ti(ii) complexes,^62^ which could serve as valuable inspiration for future qubit materials. Building on these insights, our future work will focus on developing and exploring molecular qubit candidates centered on Ti(ii).

### 2.4 Effect of geometric distortion on the zero-field splitting parameters

The computations discussed so far were performed at equilibrium geometries. The systems, however, are in a host matrix and they are subject to distortions. In previous studies^63,64^ electrostatic embedding approaches have been employed to incorporate the electrostatic influence of the host matrix in multireference calculations. In contrast, our approach focuses on deforming the molecules by following two vibrational modes, the symmetric stretch and scissoring (Fig. S23†), from which we generated eight distorted structures. We applied these distortions to the Cr(*o*-tol)_4_, V(*o*-tol)_4_^−^, and Ti(*o*-tol)_4_^2−^ complexes and analyzed how they affect the energy gaps between the ground state (T_0_) and excited states (S_1_ or T_1_), as well as the zero-field splitting parameter |*D*|. In this section, we discuss only the CASPT2 results, which have a similar trend to the tPBE0 ones.

**Fig. 3:**
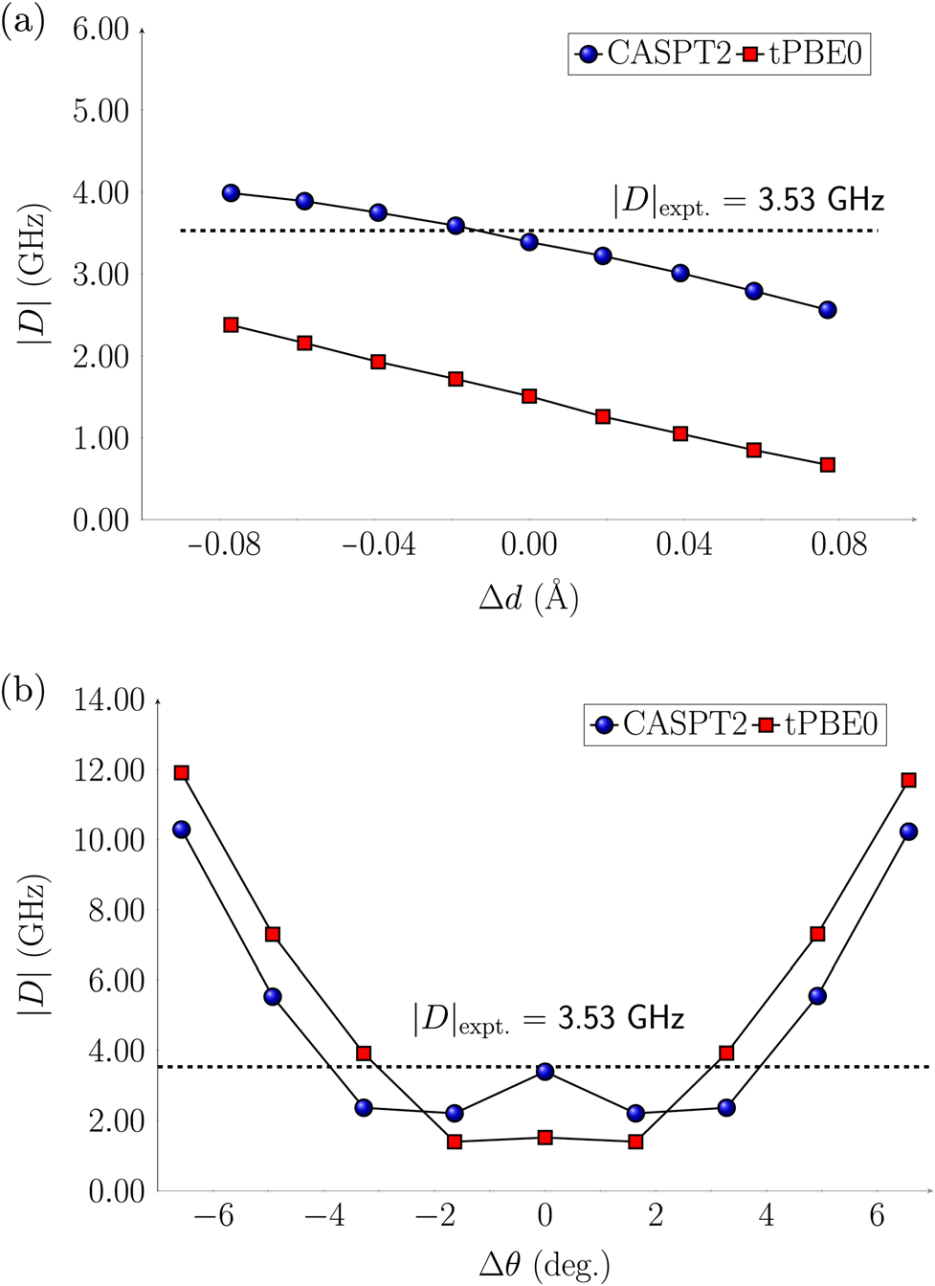
CASPT2 and tPBE0 axial parameter |*D*| of Cr(*o*-tol)_4_ complex for (a) the Cr–C symmetric stretching, and (b) the C–Cr–C scissoring normal modes. The equilibrium bond angle is *θ* = 104.9°, and the equilibrium bond distance is *d* = 1.98 Å.

For the Cr(*o*-tol)_4_ complex, the energy gaps are only slightly affected by stretching/compressing the Cr–C bonds by 0.1 Å and the ZFS parameter |*D*| varies only by approximately 1 GHz (Fig. 3 and Table S77†). However, bond angle variations of ±7° along the scissoring vibration have a more pronounced effect on |*D*| up to about 12 GHz (Fig. 3 and Table S78†). This highlights the higher sensitivity to angular deformations than bond distortion.

For the V(*o*-tol)_4_^−^ complex, variations in the V–C bond length have little effect on the energy of the S_1_ state. The T_1_ state instead lowers in energy with bond elongation and eventually crosses below S_1_, becoming the lowest-energy excited state (Fig. S26†). The T_1_–S_1_ inversion could influence the initialization process in the distorted geometry, potentially leading to fluorescence instead of non-radiative decay. As a result, the system may fail to achieve the required spin polarization, compromising initialization. Such a reversal in singlet–triplet stability warrants experimental validation, perhaps in a ligand environment that allows for a slightly larger V—ligand distance. In contrast, bond angle distortions have little effect on the energy gaps between the excited states and the ground state (Fig. S27†). The ZFS parameter |*D*| varies by approximately ±2 GHz during bond stretching and compression, while bond-angle variations affect |*D*| in a trend similar to the Cr(*o*-tol)_4_ complex: slight deformations up to ±3° reduce |*D*| from 6 to 2 GHz, whereas larger distortions, up to ±6° increase |*D*| to 12 GHz (Fig. S38†).

For the Ti(*o*-tol)_4_^2−^ complex, the change in relative energies of the T_1_ and S_1_ states is reversed to the V(*o*-tol)_4_^−^ complex: variations in the Ti–C bond length have effect on the S_1_ state which increases in energy with bond elongation and eventually crosses above T_1_ which is constant in energy despite elongation (Fig. S28†). Variations in bond angles along the scissoring vibration do not have effects on the relative energies of the excited states (Fig. S29†). For the ZFS parameter |*D*|, when the Ti–C bond is compressed |*D*| remains unchanged, while upon stretching by 0.08 Å, it decreases from its equilibrium value of 14 GHz to 7 GHz. Bond angle modifications affect |*D*| by at most 4 GHz (Fig. S40 and S41†).

In conclusion, the relative energies of the lower electronic states of the Cr(*o*-tol)_4_ complex remain largely unaffected by bond length variations, while bond angle variations significantly influence the ZFS parameter |*D*|, which increases with larger bond angles, consistent with experimental observations.^31^ V(*o*-tol)_4_^−^ shows more pronounced effects on the relative energies of T_1_ and S_1_ compared to the Cr(*o*-tol)_4_ complex, with a potential swap between the two states at larger bond elongation. Finally, the Ti(*o*-tol)_4_^2−^ complex exhibits changes in both excited-state energetics and |*D*| with bond length variations, whereas bond angle modifications have little to no impact. Overall, since molecular deformations in a host matrix are primarily associated with bond angle distortions, our findings suggest that the Ti(*o*-tol)_4_^2−^ complex is less prone to these deformations, and thus small |*D*| variations than the Cr(*o*-tol)_4_, and V(*o*-tol)_4_^−^ complexes.

## 3 Conclusion

We used multiconfigurational CASPT2 and tPBE0 methods to investigate the properties of a series of organometallic complexes as potential candidates for molecular qubits. The features under study were the energy gaps between states with different spin and zero-field splitting parameters |*D*| and |*E*|. The metals considered were first-row and Group 6 transition metals. Our results confirm prior experimental findings for Cr, V, and Mo complexes while extending the scope to new candidates. Notably, the Ti(ii) complex is identified as a promising, novel candidate for molecular qubits.

Through a systematic evaluation of vertical excitation energies and zero-field splitting parameters, we identified key trends across the transition-metal series. The vertical excitation energy of the lowest excited state (of different spin than the ground state) follows the sequence Ti(*o*-tol)_4_^2−^ < W(*o*-tol)_4_ < Mo(*o*-tol)_4_ < V(*o*-tol)_4_^−^ < Cr(*o*-tol)_4_ reflecting the progressive filling of d orbitals and corresponding changes in electronic structure. In contrast, the electronic structures of Fe(*o*-tol)_4_^2−^, Co(*o*-tol)_4_^2−^, and Ni(*o*-tol)_4_^2−^ suggest that they are unsuitable as molecular qubits, due to high-energy and unfavorable different-spin excitations. To explore these transition metals as viable candidates, alternative ligand environments would need to be considered.

Zero-field splitting calculations provided further refinement of qubit suitability. For Cr(*o*-tol)_4_, V(*o*-tol)_4_^−^, and Mo(*o*-tol)_4_ complexes, both CASPT2 and tPBE0 reproduce the experimental trend for |*D*| values. The results also highlight that the W(*o*-tol)_4_ complex does not meet the optimal threshold for |*D*|, because of its high atomic number, thereby eliminating it from consideration. Importantly, the Ti(*o*-tol)_4_^2−^ complex has a predicted |*D*| value below 20 GHz, supporting its candidacy alongside Cr(*o*-tol)_4_, V(*o*-tol)_4_^−^, and Mo(*o*-tol)_4_.

We investigated the effect of geometric distortions induced by the host matrix on the ZFS parameter |*D*| by varying the M–C bond length and C–M–C bond angle. Our analysis shows that the Ti(*o*-tol)_4_^2−^ complex is less sensitive to geometric distortions compared to Cr(*o*-tol)_4_ and V(*o*-tol)_4_^−^, whose |*D*| parameter varies significantly with bond angle. These results highlight the importance of incorporating matrix effects when modeling molecular complexes as potential qubits.

The use of Ti(ii) as a metal center for molecular qubit candidates is a promising avenue of future investigation. Stable Ti(ii) complexes documented in the literature provide a basis for further exploration.^62,65^ Future efforts from our side will focus on computational modeling and synthesizing Ti-based systems, as well as extending computational studies to explore other ligand environments and their influence on qubit properties.

## 4 Computational methods

### 4.1 Geometry optimization

Geometry optimization calculations were performed using Kohn–Sham density functional theory (KS-DFT) with Gaussian 16 rev. A03.^66^ All calculations were carried out for isolated molecules, employing the following functionals: meta-GGA hybrid TPSSh,^67,68^ hybrid B3LYP,^69–71^ meta-GGA M06,^72^ and GGA PBE,^73^ all including the empirical dispersion correction D3BJ based on Becke and Johnson damping, except for M06.^74^ The Ahlrichs basis set def2-TZVP basis set^75^ was used for all atoms, with pseudopotential def2-ECP for Mo, and W. Vibrational calculations were performed at the TPSSh-D3BJ/def2-TZVP level of theory.

### 4.2 SA-CASSCF calculations

The ground-state optimized structures served as inputs for the state-average complete active space self-consistent field (SA-CASSCF)^76^ calculations, which were employed to calculate the electronic excitation energies. These calculations were carried out using OpenMolcas v23.10.^77^

The ANO-RCC-VTZP basis set was employed for the metal centers, while ANO-RCC-VDZ basis sets were assigned to the carbon and hydrogen atoms.^78^ Scalar relativistic effects were incorporated using the second-order Douglas–Kroll–Hess Hamiltonian.^79–83^ To reduce computational costs, the resolution of the identity and Cholesky decomposition techniques were utilized.^84^

**Fig. 4:**
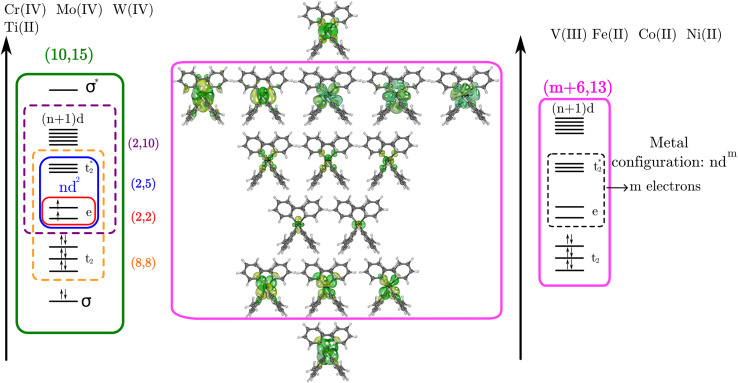
Active spaces used for the multiconfigurational calculations of molecules studied herein. On the left energy scale, it is shown the active spaces used for molecules Ti(*o*-tol)_4_^2−^, Cr(*o*-tol)_4_, Mo(*o*-tol)_4_, and W(*o*-tol)_4_. The right energy scale shows only the largest active space used for complexes V(*o*-tol)_4_^−^, Fe(*o*-tol)_4_^2−^, Co(*o*-tol)_4_^2−^, and Ni(*o*-tol)_4_^2−^.

For the Ti(*o*-tol)_4_^2−^, Cr(*o*-tol)_4_, Mo(*o*-tol)_4_, and W(*o*-tol)_4_ complexes, SA-CASSCF wave functions were computed using the active spaces shown in Fig. 4. The Ti, Cr, Mo and W metal centers have electronic configuration *n*d^2^, where *n* = 3, 3, 4, 5 respectively. The final active spaces were obtained by progressively adding orbitals starting from the smallest active space, consisting of 2 electrons in the corresponding d-shell, (2,5), formed by the *n*d_*z*^2^_ and *n*d_*x*^2^−*y*^2^_ orbitals (we refer to them as e pair implying a tetrahedral symmetry, even if these species are only pseudo tetrahedral) and the *n*d_*xy*_, *n*d_*xz*_, and *n*d_*yz*_ orbitals (collectively referred to as 
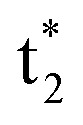
) shown in Fig. 4. A correlating set of d orbitals ((*n* + 1)d) was included to expand the active space, resulting in (2,10). By incorporating three metal–ligand molecular orbitals (*t*_2_) into the active space starting from (2,5), the configuration was extended to (8,8). Finally, the addition of the correlating d-shell along with the metal s bonding (σ) and antibonding (σ*) orbitals expanded the active space to (10,15).

The relative energies with increasing active spaces are reported in Tables S44–S70.† There is a minor active space dependence in the results, a reason for which we presented the results with the largest active space, which is more balanced. In general the composition of the wave function does not vary significantly. For example, in the systems with a triplet ground state, the triplet is dominated by the single configuration (σ)^2^(*t*_2_)^6^(*n*d_*z*^2^_)^↑^(*n*d_*x*^2^−*y*^2^_)^↑^((*n* + 1)d)^0^(σ*)^0^ while the lowest singlet has two dominant configurations, (i) (σ)^2^(*t*_2_)^6^(*n*d_*z*^2^_)^0^(*n*d_*x*^2^−*y*^2^_)^2^((*n* + 1)d)^0^(σ*)^0^ and (ii) (σ)^2^(*t*_2_)^6^(*n*d_*z*^2^_)^2^(*n*d_*x*^2^−*y*^2^_)^0^((*n* + 1)d)^0^(σ*)^0^.

Among the first-row transition metals ions, the electronic configurations are 3d^*m*^, where *m* = 2 for V^3+^, *m* = 6 for Fe^2+^, *m* = 7 for Co^2+^, and *m* = 8 for Ni^2+^. Using SA-CASSCF, the (*m*, 5) and (*m*, 10) active spaces were obtained. On the other hand, the inclusion of the σ and σ* orbitals in the active spaces of the V(*o*-tol)_4_^−^ complex faced some complications related to the inclusion of such orbitals in the active space. Also, due to the unsuitability of the Fe(*o*-tol)_4_^2−^, Co(*o*-tol)_4_^2−^, and Ni(*o*-tol)_4_^2−^ complexes as molecular qubits a larger active space was not explored. Therefore, the SA-CASSCF calculations considered 13 orbitals rather than 15, as depicted in the right part of Fig. 4. The active spaces considered consist of the 3d orbitals (*m*, 5), the inclusion of a second d–shell (*m*,10), the alternative active space where we add three metal-bonding molecular orbitals to the 3d orbitals (*m* + 6,8), and the inclusion of the second d–shell to the latter active space to obtain (*m* + 6,13).

For the Cr(*o*-tol)_4_, Mo(*o*-tol)_4_, and W(*o*-tol)_4_ complexes, 7 triplets and 9 singlets were included in the state-averaged calculations for the (10,15) active spaces. For the first-row transition metal complexes, state-averaged calculations of Ti(*o*-tol)_4_^2−^ and Ni(*o*-tol)_4_^2−^ were performed using 10 triplets and 15 singlets for the corresponding active space. For V(*o*-tol)_4_^−^ complex, 7 triplets and 9 singlets were calculated using the (8,13) active space. In the case of the Fe(*o*-tol)_4_^2−^ complex, 5 quintets, 15 triplets, and 5 singlets were considered for the (12,13) active space. For the Co(*o*-tol)_4_^2−^ complex, state-averaged calculations included 10 quartets and 30 doublets. The full discussion about these choices is in the ESI in the Zero-field splitting parameters section.†

### 4.3 CASPT2 and MC-PDFT calculations of energy gaps

The triplet–singlet energy gaps for the molecules Cr(*o*-tol)_4_, Mo(*o*-tol)_4_, W(*o*-tol)_4_, Ti(*o*-tol)_4_^2−^, V(*o*-tol)_4_^−^, and Ni(*o*-tol)_4_^2−^ were computed as vertical excitations from the triplet ground state geometry. On the other hand, the Fe(*o*-tol)_4_^2−^ and Co(*o*-tol)_4_^2−^ complexes do not have a triplet ground state but instead a quintet and quartet respectively. Therefore in these latter cases the quartet-doublet energy gap was calculated for the Co(*o*-tol)_4_^2−^ complex, and the quintet–triplet gap for the Fe(*o*-tol)_4_^2−^ complex, in both cases as vertical excitation using the ground state geometry. The reported energy gaps represent spin–orbit-free energies obtained through SA-CASSCF and post-CASSCF methods. Additionally, single-state complete active space second-order perturbation theory (CASPT2),^55^ multiconfiguration pair-density functional theory (MC-PDFT) with the tPBE functional,^47^ and hybrid MC-PDFT (HMC-PDFT) with the tPBE0 functional^57^ were employed (see ESI†). The tPBE0 functional incorporates a mix of 25% CASSCF energy with 75% tPBE energy.^57^

For the CASPT2 calculations, an ionization-potential electron affinity (IPEA) value of 0.25 a.u. and an imaginary shift of 0.30 a.u. were applied.

### 4.4 Zero-field splitting calculations

The zero-field splitting (ZFS) parameters were computed by incorporating spin–orbit coupling into the SA-CASSCF, CASPT2, tPBE and tPBE0 methods, utilizing the restricted active space state interaction (RASSI) method as implemented in OpenMolcas. The ZFS parameters were calculated for all the aforementioned active spaces using an effective Hamiltonian in a pseudospin basis approximation,^85^ as implemented in the SINGLE_ANISO module of OpenMolcas. The procedure for computing the ZFS parameters is extensively described in ref. 36, 86 and 87.

## Author contributions

A. S. and A. D. contributed equally to this work.

## Conflicts of interest

Authors declare no conflict of interest.

## Supplementary Material

SC-OLF-D5SC02544C-s001

## Data Availability

The data supporting this article have been included as part of the ESI.† OpenMolcas input files, and Cartesian coordinates of all structures are available in the all_structures.zip file.
