# Supplementary material for: Designing molecular qubits: computational insights into first-row and group 6 transition metal complexes
Source: Chem Sci. 2025 Jun 13;16(28):12896–905. doi: 10.1039/d5sc02544c (PMC12165291; doi:10.1039/d5sc02544c)
Supplement: SC-016-D5SC02544C-s001 [file SC-016-D5SC02544C-s001.pdf]

Supplementary Information (SI) for Chemical Science.
This journal is © The Royal Society of Chemistry 2025

Designing Molecular Qubits: Computational Insights into First-Row and Group 6 Transition Metal Complexes

Supporting Information

Arturo Sauza-de la Vega[§],[†] Andrea Darù[§],[†] Stephanie Nofz,[‡] and Laura Gagliardi[*,†,‡,¶]

†*Department of Chemistry, Chicago Center for Theoretical Chemistry, University of Chicago, Illinois, 60637, USA.*

‡*Pritzker School of Molecular Engineering, University of Chicago, Illinois 60637, USA.*

¶*James Franck Institute, University of Chicago, Illinois 60637, USA.*

E-mail: lgagliardi@uchicago.edu

Contents

1 Molecular Geometries **S3**

1.1 RMSD, Bond Lenghth, and Angles Values of DFT Optimized Complexes . . S3

1.2 DFT Relative Stability Between Spin-States S4

[§]These authors contributed equally to this work

2 Energy Gaps **S5**

3 Zero-Field Splitting Background Theory **S9**

4 Zero-Field Splitting Parameters **S11**

5 Relative Electronic Energies **S14**

5.1 Single State CASPT2 . . . . S14

5.2 Hybrid MC-PDFT (tPBE0) S22

5.3 Relative Energies S30

5.4 Absolute Energies S39

5.5 Absolute Energies for Zero-Field Splitting Calculations S47

6 Active Space Dependency **S53**

6.1 Energy Gaps S53

6.2 Zero-Field Splitting Parameters S55

6.3 Absolute Electronic Energies S58

6.3.1 Cr(*o*-tol)$_4$Complex S58

6.3.2 Mo(*o*-tol)$_4$Complex S61

6.3.3 W(*o*-tol)$_4$Complex S64

6.3.4 V(*o*-tol)$_4^-$Complex S66

6.3.5 Ti(*o*-tol)$_4^{2-}$Complex S69

6.3.6 Fe(*o*-tol)$_4^{2-}$Complex S72

6.3.7 Co(*o*-tol)$_4^{2-}$Complex S78

6.3.8 Ni(*o*-tol)$_4^{2-}$Complex S81

7 Geometrical Distortions Following Lower Energetic Vibrational Modes **S84**

1 Molecular Geometries

1.1 RMSD, Bond Lenghth, and Angles Values of DFT Optimized Complexes

The geometrical features of all optimized metal complexes are reported in Table S1. Specifically, metal-ligand distance, and ligand-metal-ligand angles.

Table S1: Metal-ligand bond distances in Å and angles in degrees for the TPSSh-D3BJ/def2-TZVP optimized structures at the respective spin state reported per column. The metal name is used to refer to the full compound (i.e. Cr is Cr(o-tol)$_4$).

	Ti	V	Cr	Fe	Co	Ni	Mo	W
Spin State								
	Triplet	Triplet	Triplet	Quintet	Quartet	Triplet	Triplet	Triplet
Bond length								
M–C(*o*-tol)	2.16	2.07	1.98	2.06	1.86	2.0	2.07	2.08
Angles								
C1–M–C2	106.0	105.2	104.9	105.3	98.2	100.6	105.3	105.1
C1–M–C3	111.2	111.7	111.8	111.6	115.4	107.0	111.6	111.7
C1–M–C4	110.2	111.7	111.8	111.6	115.5	133.2	111.6	111.7
C2–M–C3	111.2	111.7	111.8	111.6	115.3	106.5	111.6	111.7
C2–M–C4	110.2	111.7	111.8	111.6	115.4	106.9	111.6	111.7
C3–M–C4	106.0	105.2	104.9	105.4	98.1	100.6	105.3	105.1
Spin State								
	Singlet	Singlet	Singlet	Triplet	Doublet	Singlet	Singlet	Singlet
Bond length								
M–C(*o*-tol)	2.13	2.03	1.96	1.90	1.95	1.96	2.06	2.06
Angles								
C1–M–C2	102.8	105.1	97.2	94.8	93.6	93.6	105.5	107.7
C1–M–C3	112.9	111.7	115.9	128.1	144.5	89.2	113.8	113.4
C1–M–C4	112.9	111.7	115.9	117.3	91.6	176.7	114.4	111.4
C2–M–C3	112.9	111.7	115.9	113.3	90.4	176.7	103.5	103.0
C2–M–C4	112.9	111.7	115.9	109.1	162.4	89.2	113.8	113.5
C3–M–C4	102.8	105.1	97.2	94.3	95.0	88.2	105.5	107.7

Table S2: Root mean square deviation (RMSD) of the DFT optimized structures of all considered complexes. The triplet-singlet structures are used for all complexes beside the Fe and Co one where the quintet-triplet and the quartet-doublet are used respectively.

Complex	PBE	TPSSh	B3LYP	M06
$Ti(o\text{-tol})_4^{2-}$	0.44	0.39	0.39	0.32
$Cr(o\text{-tol})_4$	0.03	0.29	0.39	0.40
$V(o\text{-tol})_4^{-}$	0.26	0.18	0.19	0.20
$Fe(o\text{-tol})_4^{2-}$	1.53	1.39	1.43	2.00
$Co(o\text{-tol})_4^{2-}$	1.62	2.43	2.36	2.81
$Ni(o\text{-tol})_4^{2-}$	2.10	2.11	2.12	2.21
$Mo(o\text{-tol})_4$	0.20	0.22	0.21	0.26
$W(o\text{-tol})_4$	0.41	0.17	0.12	0.22

1.2 DFT Relative Stability Between Spin-States

Along group 6, the energy difference between the triplet and singlet optimized geometries decreases with increasing atomic number (Table S3). For $Cr(o\text{-tol})_4$, the energy gap is 1.55 eV and decreases to 1.07 eV for the complex $Mo(o\text{-tol})_4$, and continues decreasing the triplet-singlet gap to 0.91 for the $W(o\text{-tol})_4$ compound.

Table S3: Triplet-Singlet energy difference for the $Cr(o\text{-tol})_4$, $Mo(o\text{-tol})_4$, and $W(o\text{-tol})_4$ complexes computed with TPSSh-D3BJ/def2-TZVP. The energy differences were computed between the triplet and singlet-optimized geometries.

Metal	ΔE_{T-S} (eV)
$Cr(o\text{-tol})_4$	1.55
$Mo(o\text{-tol})_4$	1.07
$W(o\text{-tol})_4$	0.91

Similarly, along the first row, when the atomic number of the metal increases, the triplet-singlet gap increases (See Table S4). The energy gap for $Ti(o\text{-tol})_4^{2-}$ is 0.41 eV, and increases to 1.36 and 1.55 eV for $V(o\text{-tol})_4^{-}$, and $Cr(o\text{-tol})_4$ complexes, respectively. However, for $Ni(o\text{-tol})_4^{2-}$ complex, the optimized singlet molecule is lower in energy than the triplet-optimized structure. Even if the singlet is the ground state, we use the triplet-optimized molecular geometry for the multiconfigurational calculations to compare the same ligand field for all complexes and observe the net effect of the metal substitution.

Table S4: Triplet-Singlet energy differences for the $Ti(o\text{-tol})_4^{2-}$, $V(o\text{-tol})_4^{-}$, $Cr(o\text{-tol})_4$, and $Ni(o\text{-tol})_4^{2-}$ complexes computed with TPSSh-D3BJ/def2-TZVP. The energy differences were computed between the triplet and singlet-optimized geometries.

Molecule	$\Delta E_{\text{T-S}}$ (eV)
$Ti(o\text{-tol})_4^{2-}$	0.41
$V(o\text{-tol})_4^{-}$	1.36
$Cr(o\text{-tol})_4$	1.55
$Ni(o\text{-tol})_4^{2-}$	-1.00

The negative signs in Table S5 indicate that, in the case of $Fe(o\text{-tol})_4^{2-}$ ion, the triplet optimized structure is more stable than the singlet and quintet optimized geometries. However, the triplet geometry is not pseudo-tetrahedral. Therefore, for the purpose of this study, we used the quintet geometry for the multireference calculations. Similarly, for the $Co(o\text{-tol})_4^{2-}$ compound, the doublet geometry resulted to be lower in energy than the quartet structure, but only the quartet-optimized geometry displayed a pseudo-tetrahedral ligand field.

Table S5: Energy differences for the $Fe(o\text{-tol})_4^{2-}$ and $Co(o\text{-tol})_4^{2-}$ complexes computed with TPSSh-D3BJ/def2-TZVP. The energy differences were computed between the quintet, triplet, and singlet-optimized geometries of $Fe(o\text{-tol})_4^{2-}$ complex, and the quartet and doublet geometries of $Co(o\text{-tol})_4^{2-}$ complex.

Molecule	ΔE (eV)
$Fe(o\text{-tol})_4^{2-}$ (quintet-triplet)	-0.12
$Fe(o\text{-tol})_4^{2-}$ (quintet-singelt)	0.59
$Co(o\text{-tol})_4^{2-}$ (quartet-doublet)	-0.18

2 Energy Gaps

In Table S6 are shown the SA-CASSCF, CASPT2, tPBE, and tPBE0 calculated energy gaps when substituting the metal center down the group. For $Cr(o\text{-tol})_4$ complex, the experimental energy gap is 1.20 eV.[1] The SA-CASSCF method overestimates this reference by 0.48–0.70 eV, while CASPT2 computed values differ by $\pm \sim 0.20$ eV from the experiment. The tPBE method is, in all cases, underestimating the energy gap. The tPBE method underestimates the reference by 0.16 eV. However, tPBE0 shows an important agreement with

experimental reference.

The CASPT2 and tPBE0 methods provide energy values close to the experimental reference. Therefore, these two methods are the most reliable for predicting the $\Delta E_{\text{T-S}}$ gaps of the complexes studied herein. Figure S1 shows the CASPT2 and tPBE0 $\Delta E_{\text{T-S}}$ of Cr(o-tol)$_4$, Mo(o-tol)$_4$, and W(o-tol)$_4$ complexes using (10,15) active space. Consistently, we observe a decrease in the $\Delta E_{\text{T-S}}$ when substituting the chromium center for the elements down the periodic table. For example, in Figure S1 (d), the CASPT2 energy gaps for Cr(o-tol)$_4$, Mo(o-tol)$_4$, and W(o-tol)$_4$ complexes are 1.44, 0.82, and 0.65 eV, respectively. Similarly, the tPBE0 method shows a decrease in $\Delta E_{\text{T-S}}$, from 1.20 to 0.67 and 0.55 eV, respectively.

Table S6: Computed triplet-singlet gaps (ΔE_{TS})for the Ti(o-tol)$_4^{2-}$, V(o-tol)$_4^{-}$, Cr(o-tol)$_4$, Mo(o-tol)$_4$, and W(o-tol)$_4$ complexes using the triplet optimized geometries. The ΔE_{TS} were obtained with SA-CASSCF, CASPT2, tPBE, and tPBE0 methods using the (8,13) active space for V(o-tol)$_4^{-}$, and (10,15) for the other molecules. The values reported are in eV.

Complex	SA-CASSCF	CASPT2	tPBE	tPBE0
Ti(o-tol)$_4^{2-}$	0.73	0.56	0.49	0.55
V(o-tol)$_4^{-}$	1.46	1.18	0.65	0.85
Cr(o-tol)$_4$	1.69	1.44	1.04	1.20
Mo(o-tol)$_4$	1.05	0.82	0.54	0.67
W(o-tol)$_4$	0.90	0.65	0.43	0.55

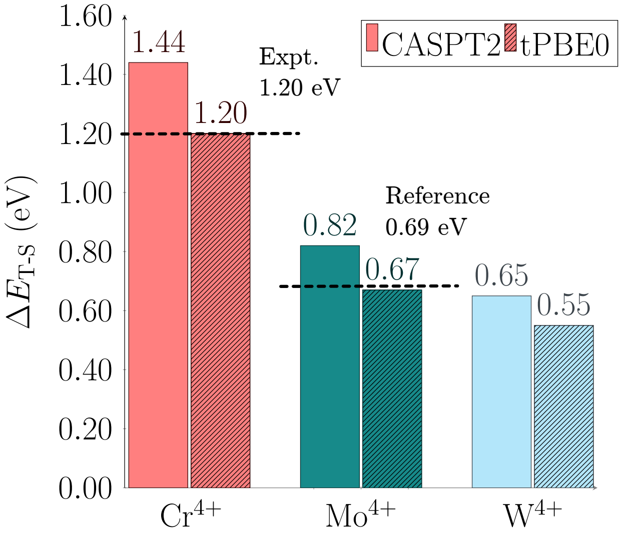

Figure S1: Calculated triplet-singlet gaps ($\Delta E_{\text{T-S}}$) with the CASPT2 (solid bar) and tPBE0 (striped bar) methods for the Cr(o-tol)$_4$ (red), Mo(o-tol)$_4$ (green) and W(o-tol)$_4$ (blue) complexes using (10,15) active space. Dashed lines correspond to reported data from references 1 and 2.

The SA-CASSCF values, like in the group analysis, display the largest values compared to the other methods. Also, the tPBE energy gaps are the smallest values for each complex and active space. This is consistent with what was observed previously for the group analysis.

Table S7: Computed triplet-singlet gaps (ΔE)for the Ni(o-tol)$_4^{2-}$ complex, quintet-triplet for Fe(o-tol)$_4^{2-}$ complex, and quartet-doublet for Co(o-tol)$_4^{2-}$ molecule. The ΔE were obtained with SA-CASSCF, CASPT2, tPBE, and the tPBE0 methods. The (n+6,13) active space was used, where $n = 6, 7$, and 8 for Fe(o-tol)$_4^{2-}$, Co(o-tol)$_4^{2-}$, and Ni(o-tol)$_4^{2-}$, respectively. The values reported are in eV.

Complex	SA-CASSCF	CASPT2	tPBE	tPBE0
Fe(o-tol)$_4^{2-}$	1.59	1.53	0.83	1.02
Co(o-tol)$_4^{2-}$	1.99	1.71	1.38	1.53
Ni(o-tol)$_4^{2-}$	1.64	1.10	0.67	0.91

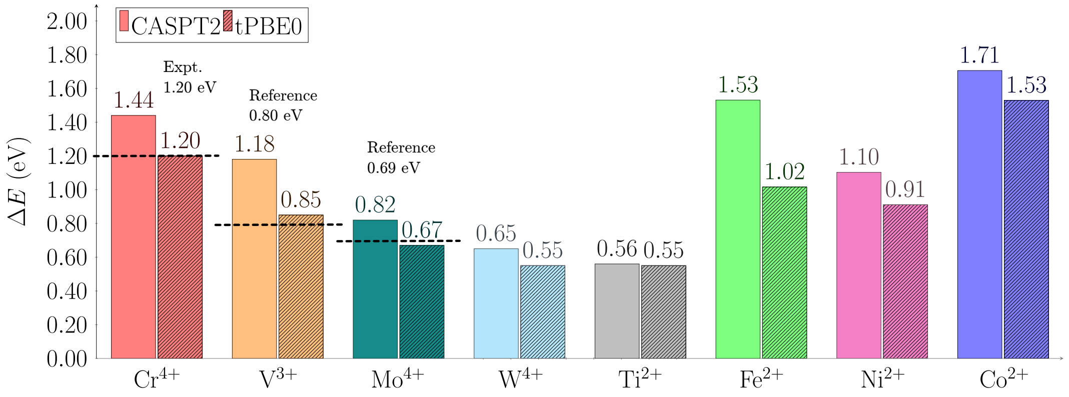

Figure S2: Calculated vertical triplet-singlet gaps for Cr(o-tol)$_4$, Ti(o-tol)$_4^{2-}$, Mo(o-tol)$_4$, W(o-tol)$_4$, and V(o-tol)$_4^-$ complexes with $n = 2$, quintet-triplet gap for Fe(o-tol)$_4^{2-}$ ($n = 6$), triplet-singlet for Ni(o-tol)$_4^{2-}$ ($n = 8$), and quartet-doublet for Co(o-tol)$_4^{2-}$ ($n = 7$). The complexes order is reported as discussed in the main text: d^2 triplet state complexes first followed by non-d^2 complexes. Energy gaps are computed with the CASPT2 (solid bar), and the tPBE0 (striped bar) methods. The red, orange, teal, cyan, gray, green, magenta, and blue colors are used for the Cr(o-tol)$_4$, V(o-tol)$_4^-$, Mo(o-tol)$_4$, W(o-tol)$_4$, Ti(o-tol)$_4^{2-}$, Fe(o-tol)$_4^{2-}$, Ni(o-tol)$_4^{2-}$, and Co(o-tol)$_4^{2-}$ complexes. For Cr(o-tol)$_4$, Mo(o-tol)$_4$, W(o-tol)$_4$, and Ti(o-tol)$_4^{2-}$ the (10,15) active space was used, and for V(o-tol)$_4^-$, Fe(o-tol)$_4^{2-}$, and Co(o-tol)$_4^{2-}$ compounds the ($n + 6$,13) active spaces. Dashed lines correspond to experimental data from references 1 and 2.

The energy differences between the triplet ground state and the first singlet excited state($\Delta E_{\mathrm{T_0-S_1}}$), the triplet ground state and first triplet excited state ($\Delta E_{\mathrm{T_0-T_1}}$), and between the triplet and singlet excited states (($\Delta E_{\mathrm{S_1-T_1}}$)) are reported in Table S8.

Table S8: Energy differences in eV between ground state T_0 and first excited state S_1, between the two triplet excited states T_0-T_1, and between the singlet and triplet excited states S_1-T_1.

	$\Delta E_{T_0-S_1}$		$\Delta E_{T_0-T_1}$		$\Delta E_{S_1-T_1}$	
Complex	CASPT2	tPBE0	CASPT2	tPBE0	CASPT2	tPBE0
Cr(o-tol)$_4$	1.44	1.20	2.10	2.23	0.66	1.03
V(o-tol)$_4^-$	1.18	0.85	1.30	1.30	0.12	0.45
Mo(o-tol)$_4$	0.82	0.67	2.57	2.57	1.75	1.90
W(o-tol)$_4$	0.65	0.55	2.31	2.21	1.66	1.66
Ti(o-tol)$_4^{2-}$	0.56	0.55	0.61	0.65	0.05	0.10

3 Zero-Field Splitting Background Theory

The ZFS parameters are obtained by solving the Hamiltonian:

$$\hat{H}_{\text{ZFS}} = \hat{\mathbf{S}} \cdot \mathbf{D} \cdot \hat{\mathbf{S}} \tag{S1}$$

where $\hat{\mathbf{S}}$ is the spin operator, and $\mathbf{D}$ is a second rank tensor that describes the ZFS.[3] By standard convention, the D-tensor is traceless and diagonalized, thus

$$D_{\text{xx}} + D_{\text{yy}} + D_{\text{zz}} = 0 \tag{S2}$$

We define the axial (D) and rhombic (E) parameters as

$$D = \frac{3}{2} D_{\text{zz}}, \quad E = \frac{1}{2}(D_{\text{xx}} - D_{\text{yy}}) \tag{S3}$$

When considering the relation between the total spin and the spatial coordinate components, $\hat{S}^2 = \hat{S}_{\text{x}}^2 + \hat{S}_{\text{y}}^2 + \hat{S}_{\text{z}}^2$, the spin Hamiltonian that describes the ZFS is given by

$$\hat{H}_{\text{ZFS}} = \frac{3}{2} D \left[\hat{S}_{\text{z}}^2 - \frac{1}{3} S(S+1) \right] + E \left(\hat{S}_{\text{x}}^2 - \hat{S}_{\text{y}}^2 \right) \tag{S4}$$

Theoretical computation of the ZFS parameters can be performed using two methodolo-

gies. In the first approach, the second-order perturbation equation is employed:

$$D_{ij} = -\frac{\zeta^2}{4S^2} \sum_{p,q} \frac{\langle \Psi_p | \hat{\ell}_i | \Psi_q \rangle \langle \Psi_q | \hat{\ell}_j | \Psi_p \rangle}{\varepsilon_q - \varepsilon_p}, \tag{S5}$$

where i and j represent the spatial components x, y, and z, and ζ, denotes the effective spin-orbit coupling constant of the metal ion. The wave functions Ψ_p and Ψ_q correspond to the ground-state p and excited states q, respectively, with energies ε_p and ε_q.[4,5] This equation is applicable only within a single spin-state manifold. To account for other manifolds, additional spin-flip terms must be included.[4,5]

In the second methodology, a pseudospin basis is constructed using selected spin states. The effective spin Hamiltonian is then used to diagonalize this basis, yielding the diagonal elements of the D-tensor. Detailed descriptions of this approach are provided in references 6–8.

It has previously been reported that density functional approximations are not the most accurate for computing zero-field splitting parameters in molecular spin qubits.[9] Consequently, active space-based methods were employed to compute such parameters. The reference multiconfigurational wave functions were obtained through state-averaged complete active space self-consistent field (SA-CASSCF)[10] calculations. The dynamic correlation was incorporated into the SA-CASSCF reference wave functions via post-CASSCF calculations. State-specific complete active space second-order perturbation theory (CASPT2),[11,12] multiconfigurational pair-density functional theory (MC-PDFT),[13] and hybrid MC-PDFT (HMC-PDFT)[14] were employed, given their favorable performance for computing magnetic properties and their accuracy comparable to the CASPT2 method.[9,15–18]

4 Zero-Field Splitting Parameters

We can compute the parameters with perturbation theory using the equation:

$$D_{ij} = -\frac{\zeta^2}{4S^2} \sum_{p,q} \frac{\left\langle \Psi_p | \hat{l}_i | \Psi_q \right\rangle \left\langle \Psi_q | \hat{l}_j | \Psi_p \right\rangle}{\epsilon_q - \epsilon_p}. \tag{S6}$$

Figure S3 (a) shows the axial parameters of Cr(o-tol)$_4$, Mo(o-tol)$_4$, and W(o-tol)$_4$ complexes in cm^{-1} units. It is observed that there is a significant trend of increasing the $|D|$ parameter with the atomic number.

A close relationship exists between the axial parameter D and the spin-orbit coupling constant ζ as highlighted in red in equation S6. Also, it is known that the spin-orbit coupling constant increases with the atomic number as Z^4.[19]

Figure S3 (a) shows the computed $|D|$ values for the Cr(o-tol)$_4$, Mo(o-tol)$_4$, and W(o-tol)$_4$ complexes. The increasing trend with the metal substitution is similar to the increasing values of the atomic number as Z^4 (Figure S3 (b)). In the literature are reported the spin-orbit coupling constants for the ions Cr(o-tol)$_4$, Mo(o-tol)$_4$, and W(o-tol)$_4$ are 325, 950, and 2,300 cm^{-1},[20] respectively. The squared of such constants (Figure S3 (c)) also present the same increasing trend as the computed $|D|$ values. Therefore, we can conclude the observed trend is due to the increase in the relativistic effects when substituting the chromium center for heavier elements.

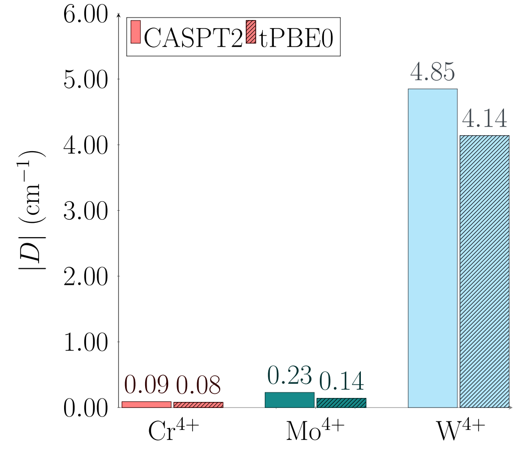

(a) Axial parameter ($|D|$)

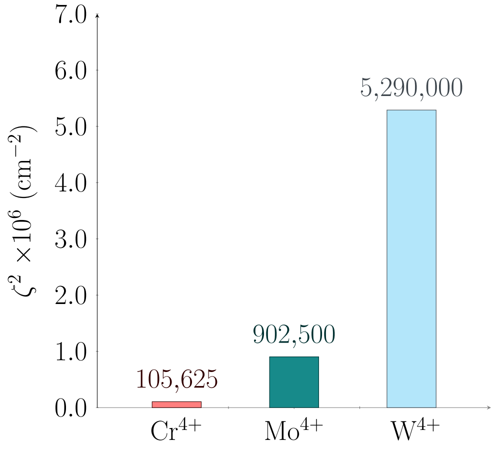

(b) Spin-orbit coupling constant (ζ^2)

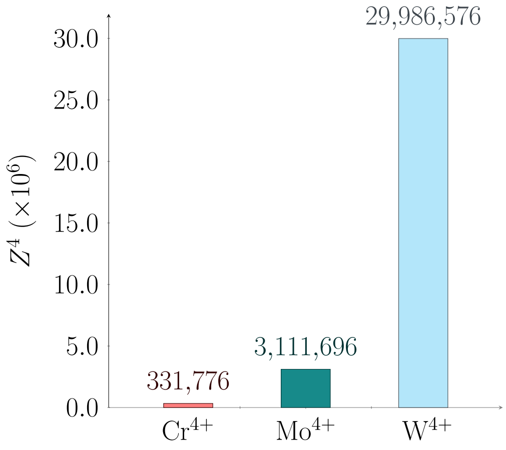

(c) Atomic number (Z^4)

Figure S3: (a) The axial parameter $|D|$ computed using (10,15) active space, (b) Spin-orbit coupling constants squared, and (c) Increasing values for the atomic numbers as Z^4. The ζ constants were obtained from reference 20.

To compute the ZFS parameters of the molecules across the period, we selected different numbers of spin-states to be included in the state-interaction step of each calculation. The description is given below.

When computing $|D|$ for the Cr(o-tol)$_4$, Mo(o-tol)$_4$, and W(o-tol)$_4$ complexes, 7 triplets, and 9 singlets were mixed with spin-orbit coupling which are the state below the 3.5, eV cutoff. This cutoff was chosen *ad-hoc* following the natural energy gap found in the relative energies of the complexes studied. The 16 spin-orbit-free states are in a range of 0.0–3.5

eV (Figures S4–S6 and S12–S14); in the V(o-tol)$_4^-$ complex, 7 triplets and 9 singlets below the 3.0 eV cutoff were used (Figures S8 and S16). Differently, for the Ti(o-tol)$_4^{2-}$ complex, 10 triplets and 15 singlets were present below the $\sim$ 2.5 eV cutoff, thus these ones were considered (Figure S7 and S15).

Increasing the number of triplets and singlets for the computation of the ZFS parameters leads to larger values, and in the case of Mo(o-tol)$_4$ complex, the increasing trend for $|D|$ of Cr(o-tol)$_4$<V(o-tol)$_4^-$<Mo(o-tol)$_4$ is broken (See Table S9). The number of triplets (T) and singlets (S) were selected based on the relative energy plots shown in Figures S4–S8 and S12–S16.

Table S9: Computed ZFS axial parameters $|D|$ for the Ti(o-tol)$_4^{2-}$, V(o-tol)$_4^-$, Cr(o-tol)$_4$, Mo(o-tol)$_4$, and W(o-tol)$_4$ complexes using the triplet optimized geometries. The $|D|$ parameters were obtained with SA-CASSCF, CASPT2, tPBE, and tPBE0 methods using the (10,15) active space for all complexes, except for V(o-tol)$_4^-$, for which the (8,13) active space was used. The letters T and S stand for Triplets and Singlets, respectively. The values reported are in GHz.

Molecule	No. states	SA-CASSCF	CASPT2	tPBE	tPBE0
Ti(o-tol)$_4^{2-}$	14T, 19S	17.28	16.15	13.15	14.49
V(o-tol)$_4^-$	10T, 15S	6.98	5.96	4.07	5.56
Cr(o-tol)$_4$	10T, 15S	4.98	3.99	2.76	3.66
Mo(o-tol)$_4$	15T, 18S	3.65	4.90	7.76	4.26
W(o-tol)$_4$	16T, 19S	817.08	946.23	816.29	823.98

Table S10: Computed rhombic parameters $|E|$ for the Ti(o-tol)$_4^{2-}$, V(o-tol)$_4^-$, Cr(o-tol)$_4$, Mo(o-tol)$_4$, and W(o-tol)$_4$ complexes using the triplet optimized geometries. The $|E|$ parameters were obtained with SA-CASSCF, CASPT2, tPBE, and tPBE0 methods using the (10,15) active space for all complexes, except for V(o-tol)$_4^-$, for which the (8,13) active space was used. The letters T and S stand for Triplets and Singlets, respectively. The values reported are in GHz.

Molecule	No. states	SA-CASSCF	CASPT2	tPBE	tPBE0
Ti(o-tol)$_4^{2-}$	14T, 19S	0.01	0.01	0.01	0.01
V(o-tol)$_4^-$	10T, 15S	0.00	0.00	0.00	0.00
Cr(o-tol)$_4$	10T, 15S	0.00	0.00	0.00	0.00
Mo(o-tol)$_4$	15T, 18S	0.10	0.58	2.19	2.10
W(o-tol)$_4$	16T, 19S	147.16	181.54	159.38	155.98

5 Relative Electronic Energies

5.1 Single State CASPT2

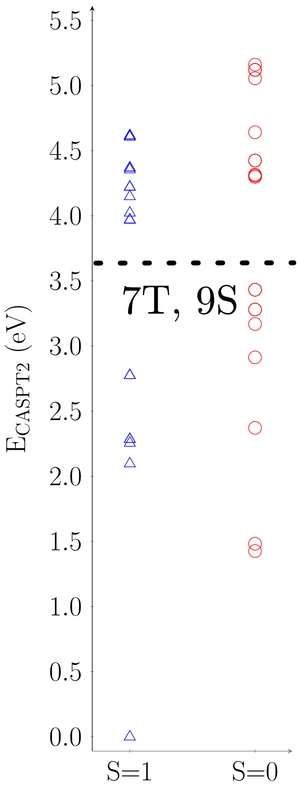

Figure S4: Relative energies of Cr(o-tol)$_4$ molecule computed with the CASPT2 method for the (10,15) active space. The blue triangles correspond to triplet states and the red circles to singlet states.

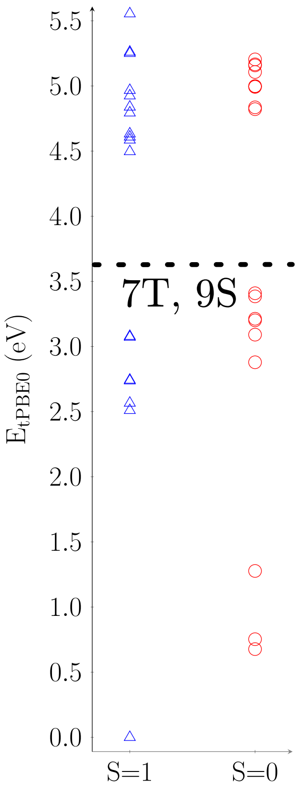

Figure S5: Relative energies of Mo(o-tol)$_4$ molecule computed with the CASPT2 method for the (10,15) active space. The blue triangles correspond to triplet states and the red circles to singlet states.

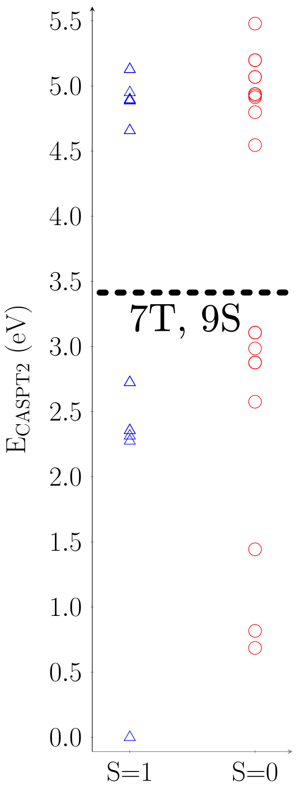

Figure S6: Relative energies of W(o-tol)$_4$ molecule computed with the CASPT2 method for the (10,15) active space. The blue triangles correspond to triplet states and the red circles to singlet states.

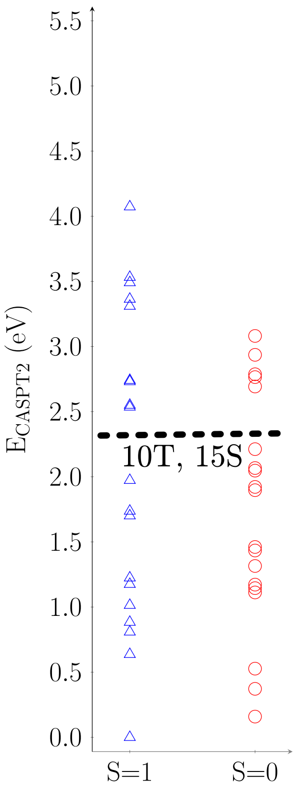

Figure S7: Relative energies of $Ti(o\text{-tol})_4^{2-}$ molecule computed with the CASPT2 method for the (10,15) active space. The blue triangles correspond to triplet states and the red circles to singlet states.

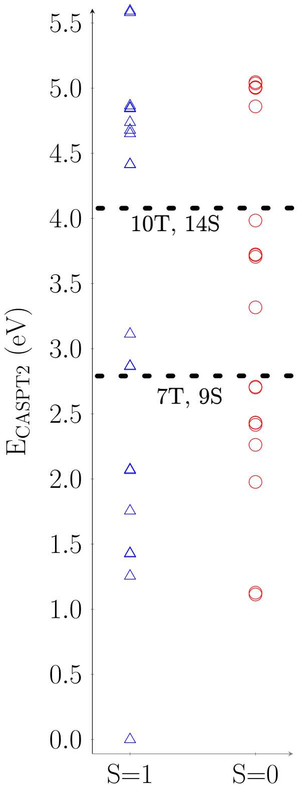

Figure S8: Relative energies of $V(o\text{-tol})_4^-$ molecule computed with the CASPT2 method for the (8,13) active space. The blue triangles correspond to triplet states and the red circles to singlet states.

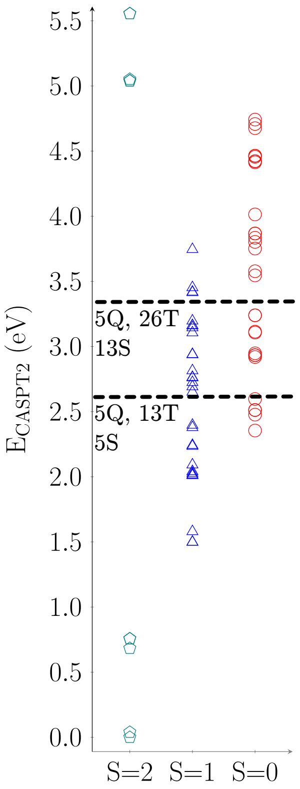

Figure S9: Relative energies of Fe(o-tol)$_4^{2-}$ molecule computed with the CASPT2 method for the (12,13) active space. The green pentagons correspond to quintet states, the blue triangles to triplet states, and the red circles to singlet states.

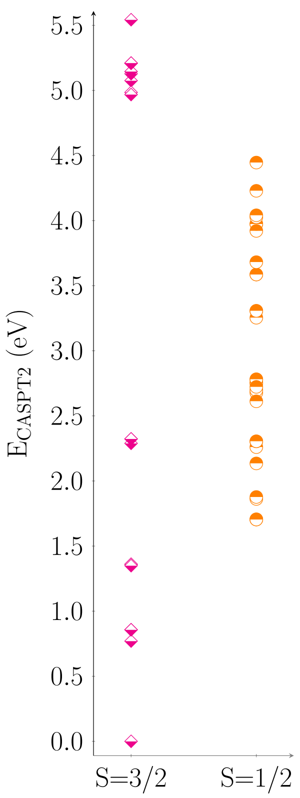

Figure S10: Relative energies of $Co(o\text{-tol})_4^{2-}$ molecule computed with the CASPT2 method for the (13,13) active space. The magenta half-filled rhombus corresponds to quartet states and the orange half-filled circles to doublet states.

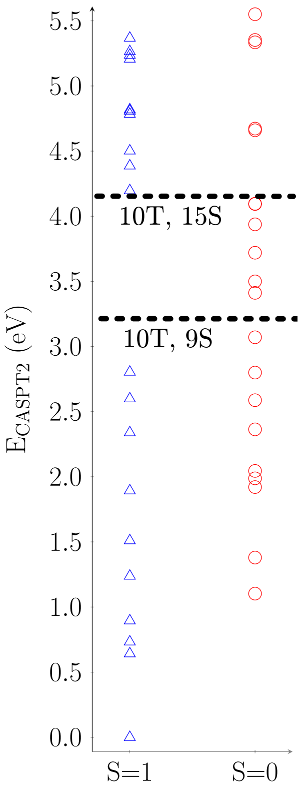

Figure S11: Relative energies of $Ni(o\text{-tol})_4^{2-}$ molecule computed with the CASPT2 method for the (14,13) active spaces. The blue triangles correspond to triplet states and the red circles to singlet states.

5.2 Hybrid MC-PDFT (tPBE0)

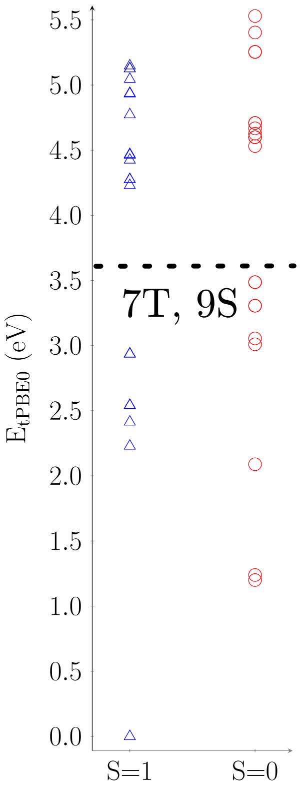

Figure S12: Relative energies of Cr(o-tol)$_4$ molecule computed with the tPBE0 method for the (10,15) active spaces. The blue triangles correspond to triplet states and the red circles to singlet states.

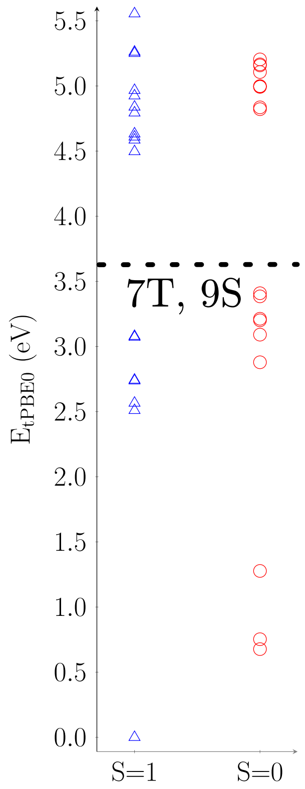

Figure S13: Relative energies of Mo(o-tol)$_4$ molecule computed with the tPBE0 method for the (10,15) active spaces. The blue triangles correspond to triplet states and the red circles to singlet states.

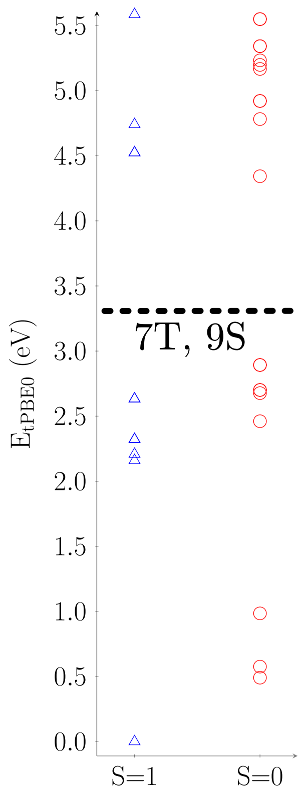

Figure S14: Relative energies of W(o-tol)$_4$ molecule computed with the tPBE0 method for the (10,15) active spaces. The blue triangles correspond to triplet states and the red circles to singlet states.

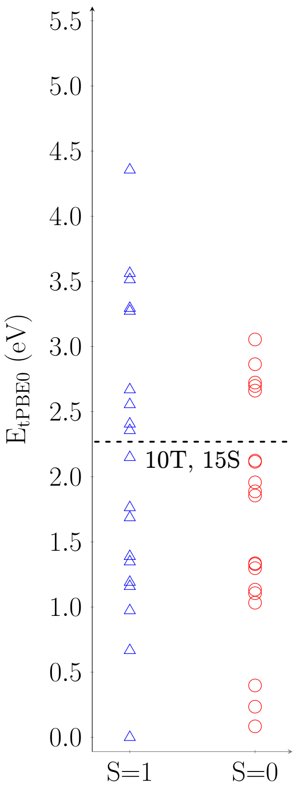

Figure S15: Relative energies of Ti(o-tol)$_4^{2-}$ molecule computed with the tPBE0 method for the (10,15) active spaces. The blue triangles correspond to triplet states and the red circles to singlet states.

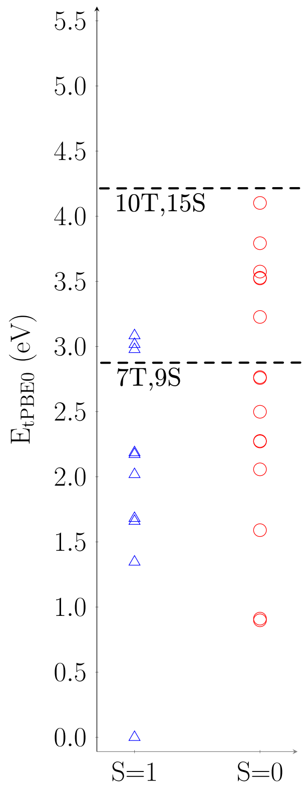

Figure S16: Relative energies of $\mathrm{V}(o\text{-tol})_4^-$ molecule computed with the tPBE0 method for the (8,13) active spaces. The blue triangles correspond to triplet states and the red circles to singlet states.

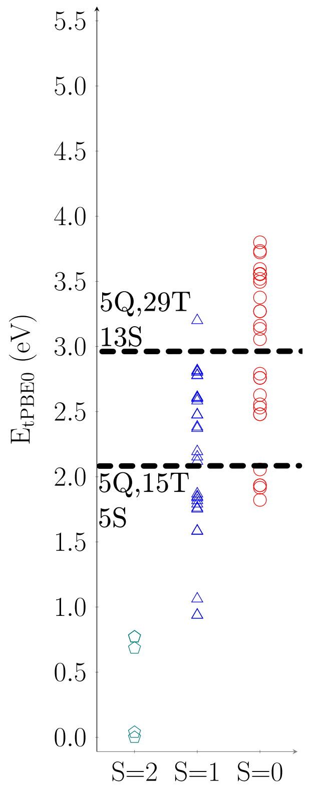

Figure S17: Relative energies of $Fe(o\text{-tol})_4^{2-}$ molecule computed with the tPBE0 method for the (12,13) active spaces. The green pentagons correspond to quintet states, the blue triangles to triplet states, and the red circles to singlet states.

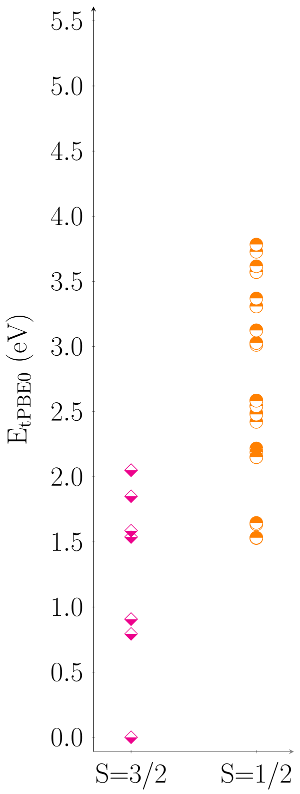

Figure S18: Relative energies of $Co(o\text{-tol})_4^{2-}$ molecule computed with the tPBE0 method for the (13,13) active spaces. The magenta half-filled rhombus corresponds to quartet states and the orange half-filled circles to doublet states.

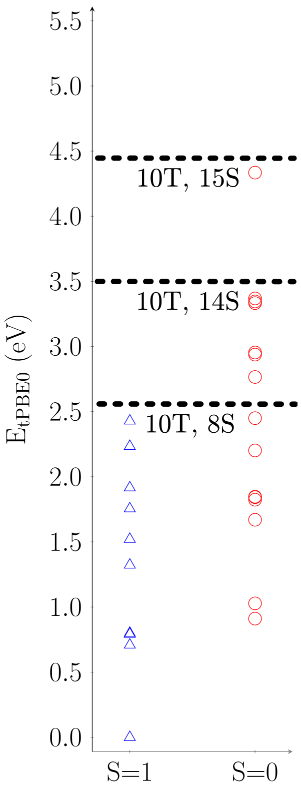

Figure S19: Relative energies of Ni(o-tol)$_4^{2-}$ molecule computed with the tPBE0 method for the (14,13) active spaces. The blue triangles correspond to triplet states and the red circles to singlet states.

5.3 Relative Energies

Table S11: Computed relative energies with the SA-CASSCF, CASPT2, tPBE, and tPBE0 methods for the relative energies of Cr(o-tol)$_4$ molecule when using the (10,15) active space. The values reported are in eV.

	SA-CASSCF		CASPT2		tPBE		tPBE0	
No.	Triplet	Singlet	Triplet	Singlet	Triplet	Singlet	Triplet	Singlet
1	0.000	1.701	0.000	1.426	0.000	1.033	0.000	1.200
2	2.496	1.764	2.097	1.482	2.140	1.064	2.229	1.239
3	2.740	2.843	2.255	2.372	2.305	1.837	2.414	2.089
4	2.768	3.495	2.284	2.913	2.465	2.909	2.541	3.055
5	2.769	3.735	2.284	3.169	2.466	2.768	2.542	3.010
6	3.342	3.933	2.775	3.280	2.799	3.097	2.935	3.306
7	3.344	3.933	2.775	3.280	2.801	3.097	2.937	3.306
8	4.387	4.101	3.969	3.432	4.238	3.282	4.275	3.487
9	4.389	4.101	3.967	3.432	4.237	3.282	4.275	3.487
10	4.484	4.846	4.022	4.300	4.145	4.517	4.230	4.600
11	4.685	4.846	4.148	4.300	4.339	4.517	4.425	4.600
12	4.690	4.922	4.220	4.315	4.387	4.529	4.463	4.627
13	4.692	4.928	4.221	4.310	4.394	4.401	4.468	4.532
14	4.940	5.016	4.368	4.424	4.931	4.606	4.933	4.709
15	4.944	5.016	4.368	4.424	4.934	4.606	4.937	4.709
16	5.046	5.447	4.354	4.641	4.682	4.408	4.773	4.668
17	5.265	5.837	4.604	5.055	4.972	5.260	5.045	5.404
18	5.290	5.925	4.615	5.160	5.071	5.399	5.126	5.530
19	5.292	6.030	4.614	5.120	5.070	4.996	5.126	5.254
20	5.309	6.030	4.614	5.120	5.094	4.996	5.148	5.255

Table S12: Computed relative energies with the SA-CASSCF, CASPT2, tPBE, and tPBE0 methods for the relative energies of Mo(o-tol)$_4$ molecule when using the (10,15) active space. The values reported are in eV.

	SA-CASSCF		CASPT2		tPBE		tPBE0	
No.	Triplet	Singlet	Triplet	Singlet	Triplet	Singlet	Triplet	Singlet
1	0.000	1.033	0.000	0.816	0.000	0.558	0.000	0.677
2	2.852	1.154	2.571	0.938	2.471	0.620	2.566	0.753
3	2.876	1.998	2.499	1.629	2.388	1.037	2.510	1.277
4	2.982	3.345	2.638	2.813	2.655	2.724	2.737	2.879
5	2.988	3.761	2.637	3.262	2.661	2.868	2.743	3.091
6	3.446	3.769	3.042	3.181	2.947	3.011	3.072	3.201
7	3.449	3.782	3.040	3.180	2.955	3.023	3.079	3.213
8	4.897	4.014	4.149	3.410	4.512	3.176	4.609	3.385
9	4.961	4.024	4.088	3.419	4.342	3.204	4.497	3.409
10	4.962	5.287	4.142	4.410	4.524	4.686	4.633	4.836
11	5.060	5.371	4.179	4.539	4.428	4.866	4.586	4.993
12	5.247	5.376	4.397	4.538	4.643	4.871	4.794	4.997
13	5.312	5.412	4.444	4.451	4.796	4.626	4.925	4.822
14	5.361	5.522	4.444	4.636	4.665	5.037	4.839	5.158
15	5.540	5.529	4.583	4.636	4.776	5.045	4.967	5.166
16	5.843	5.631	5.179	4.636	5.053	4.788	5.251	4.999
17	5.854	5.843	5.182	5.049	5.064	4.861	5.261	5.107
18	6.194	5.921	5.505	4.873	5.342	4.965	5.555	5.204
19	6.471	6.393	5.424	5.547	5.816	5.404	5.979	5.651
20	6.580	6.541	5.621	5.690	6.160	5.385	6.265	5.674

Table S13: Computed relative energies with the SA-CASSCF, CASPT2, tPBE, and tPBE0 methods for the relative energies of W(o-tol)$_4$ molecule when using the (10,15) active space. The values reported are in eV.

	SA-CASSCF		CASPT2		tPBE		tPBE0	
No.	Triplet	Singlet	Triplet	Singlet	Triplet	Singlet	Triplet	Singlet
1	0.000	0.966	0.000	0.686	0.000	0.332	0.000	0.491
2	2.493	1.099	2.314	0.817	2.112	0.401	2.208	0.576
3	2.545	1.851	2.275	1.443	2.032	0.696	2.160	0.985
4	2.576	3.100	2.356	2.576	2.237	2.248	2.322	2.461
5	2.577	3.440	2.356	2.877	2.237	2.455	2.322	2.701
6	3.025	3.440	2.724	2.877	2.503	2.455	2.633	2.701
7	3.025	3.522	2.725	2.985	2.503	2.395	2.633	2.677
8	5.108	3.718	4.658	3.107	4.330	2.617	4.524	2.893
9	5.108	3.718	4.658	3.107	4.330	2.617	4.524	2.893
10	5.388	5.230	4.896	4.546	4.525	4.047	4.741	4.343
11	6.083	5.660	4.891	4.926	5.524	4.490	5.664	4.782
12	6.083	5.877	4.891	4.799	5.524	4.931	5.664	5.167
13	6.160	5.947	4.886	4.938	5.395	5.140	5.586	5.342
14	6.229	5.947	4.950	4.938	5.429	5.140	5.629	5.342
15	6.437	6.029	5.128	5.198	5.611	4.552	5.818	4.921
16	6.437	6.029	5.128	5.198	5.611	4.552	5.818	4.921
17	7.126	6.068	5.819	4.914	6.441	4.954	6.613	5.233
18	7.294	6.162	5.968	5.069	6.589	5.346	6.765	5.550
19	7.591	6.162	6.295	5.069	6.851	5.346	7.036	5.550
20	7.591	6.335	6.295	5.479	6.851	4.820	7.036	5.198

Table S14: Computed relative energies with the SA-CASSCF, CASPT2, tPBE, and tPBE0 methods for the relative energies of Ti(o-tol)$_4^{2-}$ molecule when using the (10,15) active space. The values reported are in eV.

	SA-CASSCF		CASPT2		tPBE		tPBE0	
No.	Triplet	Singlet	Triplet	Singlet	Triplet	Singlet	Triplet	Singlet
1	0.000	0.721	0.000	0.570	0.000	0.495	0.000	0.552
2	0.657	0.959	0.605	0.712	0.641	0.500	0.645	0.615
3	1.130	1.426	0.929	0.990	1.013	0.761	1.042	0.927
4	1.131	1.752	0.928	1.399	1.013	1.169	1.042	1.315
5	1.426	2.114	0.972	1.599	1.028	1.567	1.128	1.703
6	1.560	2.185	1.152	1.635	1.347	1.638	1.400	1.775
7	1.561	2.185	1.153	1.635	1.347	1.638	1.401	1.775
8	2.167	2.419	1.694	1.800	1.623	1.636	1.759	1.832
9	2.168	2.419	1.694	1.800	1.624	1.636	1.760	1.832
10	2.306	2.699	1.852	2.199	1.804	2.095	1.930	2.246
11	5.247	2.997	4.109	2.322	4.182	2.201	4.448	2.400
12	5.292	3.165	4.184	2.475	4.379	2.249	4.607	2.478
13	5.977	3.165	4.268	2.475	5.054	2.249	5.285	2.478
14	6.549	3.464	4.993	2.741	5.169	2.492	5.514	2.735
15	6.615	4.658	4.378	3.342	5.489	2.604	5.771	3.118
16	6.628	5.347	4.377	4.120	5.500	4.465	5.782	4.686
17	6.670	6.716	4.464	5.088	5.260	5.514	5.612	5.814
18	6.747	6.735	4.950	4.476	5.281	5.608	5.647	5.889
19	6.748	6.736	4.949	4.476	5.281	5.608	5.648	5.890
20	7.050	7.161	4.669	4.754	5.618	5.695	5.976	6.061

Table S15: Computed relative energies with the SA-CASSCF, CASPT2, tPBE, and tPBE0 methods for the relative energies of V(o-tol)$_4^-$ molecule when using the (8,13) active space. The values reported are in eV.

	SA-CASSCF		CASPT2		tPBE		tPBE0	
No.	Triplet	Singlet	Triplet	Singlet	Triplet	Singlet	Triplet	Singlet
1	0.000	1.417	0.000	1.185	0.000	0.695	0.000	0.876
2	1.313	1.444	1.302	1.212	1.298	0.710	1.302	0.894
3	1.491	2.413	1.479	1.994	1.519	1.215	1.512	1.515
4	1.496	2.629	1.483	2.362	1.524	1.803	1.517	2.010
5	2.009	2.742	1.783	2.403	1.901	2.271	1.928	2.389
6	2.227	2.800	2.045	2.543	2.072	2.043	2.110	2.232
7	2.232	2.803	2.051	2.547	2.077	2.046	2.116	2.236
8	3.259	3.044	2.897	2.665	2.468	2.487	2.666	2.626
9	3.262	3.049	2.903	2.671	2.475	2.494	2.672	2.633
10	3.426	3.695	3.182	3.320	2.847	2.900	2.992	3.099
11	6.378	4.032	4.699	3.675	5.908	3.238	6.026	3.436
12	6.382	4.117	4.699	3.777	5.908	3.199	6.026	3.429
13	6.464	4.121	4.745	3.782	5.535	3.204	5.767	3.433
14	6.506	4.369	4.758	4.047	5.509	3.472	5.758	3.696
15	6.519	5.731	4.812	4.698	5.592	3.579	5.824	4.117
16	6.720	6.742	4.870	4.971	5.508	5.725	5.811	5.979
17	6.722	6.746	4.956	4.974	5.560	5.729	5.851	5.983
18	6.725	6.889	4.948	5.092	5.534	5.850	5.832	6.110
19	6.755	6.892	4.971	5.094	5.606	5.851	5.893	6.112
20	6.759	6.906	4.976	4.977	5.600	5.378	5.890	5.760

Table S16: Computed relative energies with the SA-CASSCF, CASPT2, tPBE, and tPBE0 methods for the relative energies of Fe(o-tol)$_4^{2-}$ molecule when using the (12,13) active space. The values reported are in eV.

	SA-CASSCF			CASPT2			tPBE			tPBE0		
No.	Quintet	Triplet	Singlet	Quintet	Triplet	Singlet	Quintet	Triplet	Singlet	Quintet	Triplet	Singlet
1	0.000	1.446	2.567	0.000	1.499	2.355	0.000	0.770	1.574	0.000	0.939	1.822
2	0.033	1.447	2.662	0.039	1.499	2.477	0.041	0.768	1.667	0.039	0.938	1.916
3	0.679	1.532	2.672	0.683	1.580	2.513	0.687	0.906	1.688	0.685	1.063	1.934
4	0.751	2.029	2.674	0.756	2.010	2.512	0.778	1.433	1.689	0.771	1.582	1.935
5	0.751	2.032	2.742	0.757	2.016	2.597	0.779	1.437	1.828	0.772	1.585	2.056
6	7.599	2.080	3.151	5.039	2.012	2.933	6.275	1.760	2.256	6.606	1.840	2.480
7	7.600	2.091	3.151	5.040	2.028	2.934	6.276	1.767	2.256	6.607	1.848	2.480
8	7.674	2.093	3.168	5.052	2.092	2.919	5.641	1.726	2.352	6.149	1.818	2.556
9	8.099	2.150	3.242	5.555	2.041	2.948	6.702	1.673	2.294	7.052	1.792	2.531
10	8.100	2.226	3.283	5.556	2.235	3.107	6.703	1.595	2.409	7.053	1.753	2.627
11	8.186	2.231	3.373	5.634	2.242	3.114	6.342	1.605	2.597	6.803	1.761	2.791
12	8.199	2.537	3.422	5.675	2.379	3.239	6.482	1.981	2.537	6.911	2.120	2.759
13	8.247	2.590	3.422	5.810	2.397	3.240	6.338	1.628	2.538	6.815	1.869	2.759
14	8.311	2.742	3.872	5.681	2.692	3.578	6.226	2.263	2.890	6.747	2.383	3.135
15	8.316	2.815	3.881	5.695	2.761	3.752	6.198	2.361	2.920	6.728	2.475	3.160
16	8.316	2.817	3.919	5.695	2.764	3.546	6.197	2.363	2.768	6.726	2.476	3.056
17	8.410	2.833	3.973	5.857	2.651	3.867	6.463	1.926	3.036	6.950	2.153	3.270
18	8.445	2.859	3.974	5.863	2.815	3.868	6.429	2.514	3.037	6.933	2.600	3.271
19	8.548	2.896	4.150	6.056	2.725	3.835	6.555	1.959	3.309	7.053	2.194	3.519
20	8.565	2.944	4.170	5.977	2.938	3.803	6.702	2.496	3.112	7.168	2.608	3.376
21	8.566	2.947	4.308	5.978	2.940	4.014	6.702	2.499	3.305	7.168	2.611	3.556
22	9.030	3.125	4.578	6.306	3.145	4.422	6.585	2.402	3.214	7.197	2.583	3.555
23	9.097	3.200	4.578	6.331	3.160	4.423	6.631	2.635	3.215	7.247	2.777	3.556
24	9.107	3.202	4.635	6.308	3.162	4.456	6.673	2.639	3.249	7.281	2.780	3.595
25	9.173	3.541	4.933	6.398	3.106	4.414	6.767	1.982	3.016	7.368	2.372	3.496
26	9.173	3.549	4.989	6.399	3.415	4.464	6.768	2.566	3.079	7.369	2.812	3.556
27	9.877	3.552	4.991	6.863	3.419	4.465	6.612	2.571	3.081	7.428	2.816	3.558
28	9.934	3.564	5.156	6.853	3.453	4.742	7.046	2.550	3.260	7.768	2.803	3.734
29	9.935	3.615	5.221	6.853	3.197	4.676	7.045	2.096	3.222	7.768	2.476	3.722
30	9.951	3.664	5.253	6.842	3.746	4.708	6.613	3.046	3.315	7.447	3.200	3.800

Table S17: Computed relative energies with the SA-CASSCF, CASPT2, tPBE, and tPBE0 methods for the relative energies of $Co(o\text{-tol})_4^{2-}$ molecule when using the (13,13) active space. The values reported are in eV.

	SA-CASSCF		CASPT2		tPBE		tPBE0	
No.	Quartet	Doublet	Quartet	Doublet	Quartet	Doublet	Quartet	Doublet
1	0.000	1.991	0.000	1.706	0.000	1.376	0.000	1.529
2	0.759	1.994	0.770	1.704	0.806	1.378	0.794	1.532
3	0.835	1.994	0.857	1.704	0.931	1.378	0.907	1.532
4	0.835	2.091	0.857	1.862	0.931	1.483	0.907	1.635
5	1.379	2.105	1.360	1.877	1.591	1.492	1.538	1.646
6	1.379	2.380	1.360	2.135	1.591	2.164	1.538	2.218
7	1.395	2.616	1.349	2.260	1.649	2.045	1.586	2.188
8	2.735	2.650	2.289	2.304	1.555	1.984	1.850	2.150
9	2.745	2.650	2.323	2.305	1.819	1.984	2.050	2.150
10	2.745	2.879	2.323	2.678	1.819	2.359	2.050	2.489
11	7.670	2.937	5.075	2.752	6.044	2.469	6.451	2.586
12	7.674	2.937	4.985	2.753	5.967	2.469	6.394	2.586
13	7.674	2.959	4.968	2.697	5.991	2.242	6.412	2.421
14	7.691	3.005	5.128	2.612	6.041	2.303	6.454	2.478
15	7.701	3.035	5.145	2.781	6.032	2.378	6.450	2.542
16	7.917	3.035	5.208	2.781	6.054	2.378	6.519	2.542
17	7.947	3.106	5.210	2.723	6.009	2.409	6.493	2.583
18	7.947	3.560	5.210	3.254	6.009	2.829	6.493	3.012
19	8.217	3.571	5.543	3.307	6.397	2.846	6.852	3.027
20	8.286	3.571	5.622	3.307	6.394	2.846	6.867	3.027
21	8.416	3.766	5.699	3.586	6.336	3.154	6.856	3.307
22	8.545	3.766	5.902	3.586	6.354	3.154	6.902	3.307
23	8.615	3.831	5.924	3.681	6.453	3.215	6.994	3.369
24	8.615	4.073	5.924	3.974	6.453	3.403	6.994	3.571
25	8.676	4.074	5.935	3.974	6.444	3.403	7.002	3.571
26	8.746	4.102	5.871	4.026	6.279	3.453	6.896	3.616
27	8.771	4.513	6.046	4.229	6.614	3.464	7.153	3.727
28	8.771	4.537	6.047	4.446	6.614	3.532	7.153	3.783
29	8.985	4.606	6.310	3.921	6.853	2.631	7.386	3.125
30	8.985	4.870	6.310	4.041	6.852	2.540	7.386	3.123

Table S18: Computed relative energies with the SA-CASSCF, CASPT2, tPBE, and tPBE0 methods for the relative energies of Ni(o-tol)$_4^{2-}$ molecule when using the (14,13) active space. The values reported are in eV.

	SA-CASSCF		CASPT2		tPBE		tPBE0	
No.	Triplet	Singlet	Triplet	Singlet	Triplet	Singlet	Triplet	Singlet
1	0.000	1.500	0.000	1.034	0.000	0.597	0.000	0.823
2	0.630	1.782	0.691	1.372	0.819	0.667	0.772	0.946
3	0.694	2.319	0.768	1.981	0.889	1.406	0.840	1.635
4	0.836	2.404	0.951	1.991	0.941	1.504	0.915	1.729
5	1.194	2.414	1.287	2.078	1.543	1.598	1.456	1.802
6	1.424	2.943	1.572	2.396	1.779	1.430	1.690	1.809
7	1.799	3.076	1.965	2.637	1.979	2.209	1.934	2.426
8	2.747	3.226	2.397	2.836	1.791	1.837	2.030	2.185
9	2.983	3.478	2.626	3.083	2.093	2.736	2.316	2.922
10	3.144	3.776	2.809	3.543	2.312	2.603	2.520	2.896
11	7.500	3.787	4.555	3.555	6.189	2.603	6.517	2.899
12	7.527	4.000	4.491	3.779	5.778	3.140	6.216	3.355
13	7.648	4.255	4.619	4.257	6.349	3.217	6.674	3.476
14	8.046	4.284	5.056	4.249	6.171	3.167	6.640	3.446
15	8.120	6.830	4.960	5.501	6.008	3.627	6.536	4.428
16	8.156	7.469	4.978	4.243	6.098	5.827	6.613	6.237
17	8.356	8.118	5.331	4.854	6.303	6.096	6.816	6.602
18	8.420	8.242	5.328	4.870	6.458	6.377	6.948	6.843
19	8.548	8.756	5.535	5.733	6.693	6.196	7.156	6.836
20	8.686	8.774	5.590	5.669	6.457	6.182	7.014	6.830

5.4 Absolute Energies

Table S19: Computed absolute energies with the SA-CASSCF, CASPT2, tPBE, and tPBE0 methods for the relative energies in Figures S4 and S12 of Cr(o-tol)$_4$ molecule when using the (10,15) active space.

	SA-CASSCF		CASPT2		tPBE		tPBE0	
No.	Triplet	Singlet	Triplet	Singlet	Triplet	Singlet	Triplet	Singlet
1	-2126.47618830	-2126.41356374	-2129.40563868	-2129.35271166	-2133.28573804	-2133.24721356	-2131.58335061	-2131.53880111
2	-2126.38844137	-2126.41186793	-2129.32870869	-2129.35105113	-2133.20851813	-2133.24636305	-2131.50349894	-2131.53773927
3	-2126.37985386	-2126.37138764	-2129.32357574	-2129.31780887	-2133.19893847	-2133.21789096	-2131.49416732	-2131.50626513
4	-2126.37985244	-2126.34718574	-2129.32357631	-2129.29849510	-2133.19893792	-2133.17773266	-2131.49416655	-2131.47009593
5	-2126.37737144	-2126.33987031	-2129.32184779	-2129.29063913	-2133.20136262	-2133.18460486	-2131.49536483	-2131.47342122
6	-2126.35914341	-2126.33521348	-2129.30557911	-2129.28699005	-2133.18644301	-2133.17508987	-2131.47961811	-2131.46512077
7	-2126.35914250	-2126.33521330	-2129.30557905	-2129.28698995	-2133.18644173	-2133.17508967	-2131.47961692	-2131.46512058
8	-2126.30885914	-2126.32992037	-2129.25806800	-2129.28327992	-2133.12474874	-2133.16516573	-2131.42077634	-2131.45635439
9	-2126.30885700	-2126.32992011	-2129.25807101	-2129.28327955	-2133.12475187	-2133.16516512	-2131.42077815	-2131.45635387
10	-2126.30473836	-2126.29574305	-2129.25620875	-2129.24880493	-2133.12734398	-2133.12104870	-2131.42169258	-2131.41472229
11	-2126.29893248	-2126.29574304	-2129.25008163	-2129.24880485	-2133.12015229	-2133.12104862	-2131.41484734	-2131.41472223
12	-2126.29893054	-2126.29247010	-2129.25008121	-2129.24723648	-2133.12014448	-2133.12075467	-2131.41484100	-2131.41368353
13	-2126.29592867	-2126.29074238	-2129.24854909	-2129.24391650	-2133.11780763	-2133.11627091	-2131.41233789	-2131.40988878
14	-2126.29300141	-2126.29074228	-2129.24538615	-2129.24391636	-2133.10105876	-2133.11627045	-2131.39904442	-2131.40988841
15	-2126.29299749	-2126.29055431	-2129.24538610	-2129.24451464	-2133.10105421	-2133.11422472	-2131.39904003	-2131.40830712
16	-2126.28671722	-2126.27636513	-2129.24430592	-2129.23695798	-2133.10852511	-2133.12412141	-2131.40307314	-2131.41218234
17	-2126.28577367	-2126.25937556	-2129.24273209	-2129.22435950	-2133.12331582	-2133.08951273	-2131.41393028	-2131.38197844
18	-2126.28577173	-2126.25746186	-2129.24273153	-2129.21984837	-2133.12331487	-2133.10419175	-2131.41392909	-2131.39250928
19	-2126.28224922	-2126.25746162	-2129.23841297	-2129.21984808	-2133.09864713	-2133.10419158	-2131.39454765	-2131.39250909
20	-2126.28177455	-2126.25707948	-2129.23806713	-2129.22099506	-2133.10126953	-2133.10526669	-2131.39639579	-2131.39321989

Table S20: Computed absolute energies with the SA-CASSCF, CASPT2, tPBE, and tPBE0 methods for the relative energies in Figures S5 and S13 of Mo(o-tol)$_4$ molecule when using the (10,15) active space.

	SA-CASSCF		CASPT2		tPBE		tPBE0	
No.	Triplet	Singlet	Triplet	Singlet	Triplet	Singlet	Triplet	Singlet
1	-5122.57076289	-5122.53210926	-5125.47422173	-5125.44351824	-5130.30002591	-5130.27914473	-5128.36771016	-5128.34238586
2	-5122.46343769	-5122.52836194	-5125.37718974	-5125.43975048	-5130.20711656	-5130.27711836	-5128.27119684	-5128.33992926
3	-5122.46110829	-5122.49682362	-5125.37849058	-5125.41393963	-5130.20884746	-5130.26191469	-5128.27191267	-5128.32064192
4	-5122.46086774	-5122.44373694	-5125.37693655	-5125.36675695	-5130.20244798	-5130.19651515	-5128.26705292	-5128.25832060
5	-5122.46063466	-5122.43169598	-5125.37693649	-5125.35688377	-5130.20220507	-5130.18938837	-5128.26681247	-5128.24996527
6	-5122.44448387	-5122.43131110	-5125.36285203	-5125.35696322	-5130.19260459	-5130.18898167	-5128.25557441	-5128.24956403
7	-5122.44434501	-5122.42948579	-5125.36289290	-5125.35142474	-5130.19226851	-5130.19232468	-5128.25528764	-5128.25161496
8	-5122.39165678	-5122.42326739	-5125.32262592	-5125.34900389	-5130.13531485	-5130.18446805	-5128.19940033	-5128.24416789
9	-5122.38938431	-5122.42298464	-5125.32294649	-5125.34874110	-5130.13508463	-5130.18348868	-5128.19865955	-5128.24336267
10	-5122.38907082	-5122.37671881	-5125.32430843	-5125.31221457	-5130.14086848	-5130.12808800	-5128.20291907	-5128.19024570
11	-5122.38494837	-5122.37431640	-5125.32058843	-5125.30817713	-5130.13736510	-5130.12196221	-5128.19926092	-5128.18505076
12	-5122.37909415	-5122.37412925	-5125.31370286	-5125.30820795	-5130.13085866	-5130.12179999	-5128.19291753	-5128.18488231
13	-5122.37678767	-5122.37269095	-5125.31202470	-5125.31115507	-5130.12516064	-5130.13066363	-5128.18806740	-5128.19117046
14	-5122.37451738	-5122.36912189	-5125.31164430	-5125.30480292	-5130.12937367	-5130.11597039	-5128.19065960	-5128.17925827
15	-5122.36853566	-5122.36884837	-5125.30694314	-5125.30480461	-5130.12569016	-5130.11561700	-5128.18640154	-5128.17892484
16	-5122.35327080	-5122.36434593	-5125.28094000	-5125.30411283	-5130.11222569	-5130.12464516	-5128.17248697	-5128.18457035
17	-5122.35283766	-5122.35522096	-5125.28078532	-5125.29654202	-5130.11182644	-5130.11928385	-5128.17207925	-5128.17826813
18	-5122.34351639	-5122.35058729	-5125.27205506	-5125.28229481	-5130.10478870	-5130.11673353	-5128.16447062	-5128.17519697
19	-5122.33229885	-5122.33609025	-5125.27369191	-5125.27023645	-5130.08501474	-5130.10224960	-5128.14683577	-5128.16070976
20	-5122.32744796	-5122.32741735	-5125.26600346	-5125.26200585	-5130.07260451	-5130.09993434	-5128.13631537	-5128.15680509

Table S21: Computed absolute energies with the SA-CASSCF, CASPT2, tPBE, and tPBE0 methods for the relative energies in Figures S6 and S14 of W(o-tol)$_4$ molecule when using the (10,15) active space.

	SA-CASSCF		CASPT2		tPBE		tPBE0	
No.	Triplet	Singlet	Triplet	Singlet	Triplet	Singlet	Triplet	Singlet
1	-17204.30088392	-17204.26866065	-17207.19989114	-17207.17294089	-17215.61304871	-17215.59622405	-17212.78500751	-17212.76433320
2	-17204.21364958	-17204.26430606	-17207.11374566	-17207.16881359	-17215.53548029	-17215.59420088	-17212.70502261	-17212.76172718
3	-17204.21353512	-17204.23732965	-17207.11501596	-17207.14514343	-17215.53433839	-17215.58384122	-17212.70413757	-17212.74721333
4	-17204.21346835	-17204.19459055	-17207.11503187	-17207.10228776	-17215.53430277	-17215.52455553	-17212.70409417	-17212.69206429
5	-17204.21076954	-17204.18506371	-17207.11613637	-17207.09474342	-17215.53881852	-17215.52072046	-17212.70680628	-17212.68680627
6	-17204.19613594	-17204.18498499	-17207.10187860	-17207.09470994	-17215.52471914	-17215.52073321	-17212.69257334	-17212.68679616
7	-17204.19610145	-17204.18034268	-17207.10185327	-17207.08772683	-17215.52478786	-17215.52008027	-17212.69261626	-17212.68514587
8	-17204.12348610	-17204.17654511	-17207.02929311	-17207.08578866	-17215.45715511	-17215.51443880	-17212.62373786	-17212.67996538
9	-17204.12343171	-17204.17647825	-17207.02928811	-17207.08575534	-17215.45713891	-17215.51422850	-17212.62371211	-17212.67979094
10	-17204.11674097	-17204.11966351	-17207.02320608	-17207.02918922	-17215.45247158	-17215.45813132	-17212.61853893	-17212.62351437
11	-17204.09730144	-17204.10956386	-17207.03512082	-17207.01969439	-17215.42858242	-17215.44696701	-17212.59576218	-17212.61261622
12	-17204.09146068	-17204.09342890	-17207.03709824	-17207.00664829	-17215.43341892	-17215.44115199	-17212.59792936	-17212.60422122
13	-17204.08729694	-17204.09329109	-17207.03418622	-17207.00652624	-17215.43234242	-17215.44095423	-17212.59608105	-17212.60403845
14	-17204.08397213	-17204.08444955	-17207.03014949	-17206.99849457	-17215.42912738	-17215.43521048	-17212.59283857	-17212.59752025
15	-17204.07935767	-17204.08179197	-17207.02501305	-17207.02160853	-17215.42066072	-17215.41237493	-17212.58533496	-17212.57972919
16	-17204.07621026	-17204.07426325	-17207.02320797	-17207.01978768	-17215.41561177	-17215.41356116	-17212.58076139	-17212.57873668
17	-17204.07478982	-17204.07212258	-17207.01286620	-17207.01328291	-17215.42773040	-17215.42288217	-17212.58949526	-17212.58519227
18	-17204.05660618	-17204.07188866	-17206.99642981	-17207.01997236	-17215.40941578	-17215.41122426	-17212.57121338	-17212.57639036
19	-17204.05375623	-17204.07076673	-17207.00002886	-17207.02004677	-17215.39505086	-17215.41367094	-17212.55972720	-17212.57794489
20	-17204.04749954	-17204.06596411	-17206.99415183	-17206.98771797	-17215.38955392	-17215.43057034	-17212.55404033	-17212.58941878

Table S22: Computed absolute energies with the SA-CASSCF, CASPT2, tPBE, and tPBE0 methods for the relative energies in Figures S7 and S15 of Ti(o-tol)$_4^{2-}$ molecule when using the (10,15) active space.

	SA-CASSCF		CASPT2		tPBE		tPBE0	
No.	Triplet	Singlet	Triplet	Singlet	Triplet	Singlet	Triplet	Singlet
1	-1929.56112982	-1929.53464811	-1932.38605538	-1932.36510569	-1936.26308029	-1936.24487492	-1934.58759267	-1934.56731822
2	-1929.53697293	-1929.52589171	-1932.36381450	-1932.35987600	-1936.23953148	-1936.24470474	-1934.56389184	-1934.56500148
3	-1929.51960472	-1929.50870890	-1932.35193110	-1932.34965843	-1936.22584283	-1936.23512528	-1934.54928330	-1934.55352119
4	-1929.51957750	-1929.49675110	-1932.35195099	-1932.33465072	-1936.22586204	-1936.22010511	-1934.54929091	-1934.53926661
5	-1929.50872030	-1929.48346010	-1932.35033910	-1932.32730178	-1936.22529815	-1936.20550045	-1934.54615369	-1934.52499036
6	-1929.50380912	-1929.48082208	-1932.34370507	-1932.32598100	-1936.21359065	-1936.20286899	-1934.53614527	-1934.52235726
7	-1929.50377894	-1929.48082135	-1932.34369587	-1932.32598113	-1936.21357097	-1936.20286835	-1934.53612296	-1934.52235660
8	-1929.48149118	-1929.47223213	-1932.32380707	-1932.31989720	-1936.20344495	-1936.20297044	-1934.52295651	-1934.52028586
9	-1929.48146038	-1929.47223169	-1932.32378377	-1932.31989762	-1936.20339655	-1936.20297109	-1934.52291251	-1934.52028624
10	-1929.47638415	-1929.46195008	-1932.31800663	-1932.30523415	-1936.19677999	-1936.18609340	-1934.51668103	-1934.50505757
11	-1929.36831968	-1929.45098772	-1932.23504435	-1932.30072129	-1936.10940352	-1936.18218060	-1934.42413256	-1934.49938238
12	-1929.36665352	-1929.44480404	-1932.23230500	-1932.29508490	-1936.10215310	-1936.18041432	-1934.41827821	-1934.49651175
13	-1929.34148216	-1929.44480377	-1932.22920718	-1932.29508412	-1936.07733611	-1936.18041566	-1934.39337262	-1934.49651269
14	-1929.32046783	-1929.43383217	-1932.20256012	-1932.28532798	-1936.07313653	-1936.17151905	-1934.38496936	-1934.48709733
15	-1929.31803658	-1929.38993502	-1932.22516586	-1932.26323510	-1936.06135554	-1936.16738309	-1934.37552580	-1934.47302107
16	-1929.31754532	-1929.36462225	-1932.22521031	-1932.23465436	-1936.06097709	-1936.09898674	-1934.37511915	-1934.41539562
17	-1929.31600851	-1929.31430716	-1932.22200024	-1932.19906273	-1936.06979051	-1936.06045511	-1934.38134501	-1934.37391812
18	-1929.31319042	-1929.31361451	-1932.20415746	-1932.22155196	-1936.06902411	-1936.05700761	-1934.38006569	-1934.37115934
19	-1929.31314297	-1929.31360474	-1932.20419646	-1932.22155436	-1936.06900524	-1936.05699960	-1934.38003967	-1934.37115089
20	-1929.30204140	-1929.29798478	-1932.21446019	-1932.21133777	-1936.05663611	-1936.05380743	-1934.36798743	-1934.36485177

Table S23: Computed absolute energies with the SA-CASSCF, CASPT2, tPBE, and tPBE0 methods for the relative energies in Figures S8 and S16 of $V(o\text{-tol})_4^-$ molecule when using the (8,13) active space.

	SA-CASSCF		CASPT2		tPBE		tPBE0	
No.	Triplet	Singlet	Triplet	Singlet	Triplet	Singlet	Triplet	Singlet
1	-2025.05164243	-2024.99796795	-2027.94286453	-2027.89856034	-2031.82275164	-2031.79731936	-2030.12997434	-2030.09748151
2	-2025.00399064	-2024.99703302	-2027.89523609	-2027.89758846	-2031.77595598	-2031.79683609	-2030.08296465	-2030.09688532
3	-2024.99862166	-2024.96103323	-2027.89066537	-2027.86857128	-2031.76833393	-2031.77823360	-2030.07590586	-2030.07393351
4	-2024.99861744	-2024.95355120	-2027.89065870	-2027.85504006	-2031.76833149	-2031.75669304	-2030.07590298	-2030.05590758
5	-2024.97828660	-2024.94860533	-2027.87733696	-2027.85295156	-2031.75308376	-2031.73857075	-2030.05938447	-2030.04107940
6	-2024.97139227	-2024.94853896	-2027.86950908	-2027.85157426	-2031.74451050	-2031.74642336	-2030.05123094	-2030.04695226
7	-2024.97138784	-2024.94853828	-2027.86950692	-2027.85156489	-2031.74450607	-2031.74642286	-2030.05122651	-2030.04695172
8	-2024.93336872	-2024.93882666	-2027.83619126	-2027.84462330	-2031.72685464	-2031.73117995	-2030.02848316	-2030.03309163
9	-2024.93336680	-2024.93882365	-2027.83618801	-2027.84461877	-2031.72685324	-2031.73117259	-2030.02848163	-2030.03308536
10	-2024.92850517	-2024.91438090	-2027.82887337	-2027.81967596	-2031.72339266	-2031.71684034	-2030.02467079	-2030.01622548
11	-2024.80274933	-2024.90399882	-2027.77161509	-2027.80899817	-2031.59152007	-2031.70676777	-2029.89432739	-2030.00607553
12	-2024.80271363	-2024.89981022	-2027.77162146	-2027.80433002	-2031.59151154	-2031.70503802	-2029.89431206	-2030.00373107
13	-2024.80044114	-2024.89980927	-2027.77289371	-2027.80432969	-2031.60969416	-2031.70503747	-2029.90738091	-2030.00373042
14	-2024.79913864	-2024.89194892	-2027.77067673	-2027.79603682	-2031.60965644	-2031.69869482	-2029.90702699	-2029.99700835
15	-2024.79880771	-2024.83839995	-2027.77058247	-2027.76895903	-2031.60805457	-2031.69520167	-2029.90574286	-2029.98100124
16	-2024.79267958	-2024.79368743	-2027.76706903	-2027.76001840	-2031.61034777	-2031.60286842	-2029.90593072	-2029.90057317
17	-2024.79169763	-2024.79368306	-2027.76261075	-2027.76001509	-2031.60956521	-2031.60286604	-2029.90509832	-2029.90057030
18	-2024.79169350	-2024.78901780	-2027.76260504	-2027.75883522	-2031.60956172	-2031.61660942	-2029.90509467	-2029.90971152
19	-2024.78865833	-2024.78901482	-2027.75799905	-2027.75883697	-2031.60248493	-2031.61660472	-2029.89902828	-2029.90970725
20	-2024.78862283	-2024.78889670	-2027.75802810	-2027.75960405	-2031.60266081	-2031.61886229	-2029.89915132	-2029.91137089

Table S24: Computed absolute energies with the SA-CASSCF, CASPT2, tPBE, and tPBE0 methods for the relative energies in Figures S9 and S17 of Fe(o-tol)$_4^{2-}$ molecule when using the (12,13) active space.

	SA-CASSCF			CASPT2			tPBE			tPBE0		
No.	Quintet	Triplet	Singlet	Quintet	Triplet	Singlet	Quintet	Triplet	Singlet	Quintet	Triplet	Singlet
1	-2348.1112424	-2348.0580983	-2348.0168974	-2351.1050636	-2351.0499631	-2351.0185024	-2355.0305745	-2355.0022956	-2354.9727318	-2353.3007415	-2353.2662463	-2353.2337732
2	-2348.1100255	-2348.0580676	-2348.0134111	-2351.1036275	-2351.0499674	-2351.0140527	-2355.0290796	-2355.0023501	-2354.9693245	-2353.2993161	-2353.2662795	-2353.2303461
3	-2348.0862805	-2348.0549480	-2348.0130416	-2351.0799657	-2351.0469981	-2351.0127263	-2355.0053400	-2354.9972687	-2354.9685455	-2353.2755751	-2353.2616885	-2353.2296695
4	-2348.0836525	-2348.0366860	-2348.0129862	-2351.0772726	-2351.0311835	-2351.0127459	-2355.0019803	-2354.9779256	-2354.9684913	-2353.2723983	-2353.2426157	-2353.2296150
5	-2348.0836347	-2348.0365696	-2348.0104640	-2351.0772556	-2351.0309669	-2351.0096137	-2355.0019630	-2354.9777818	-2354.9634036	-2353.2723810	-2353.2424787	-2353.2251687
6	-2347.8319916	-2348.0348038	-2347.9954566	-2350.9198791	-2351.0311064	-2350.9972891	-2354.7999548	-2354.9659042	-2354.9476553	-2353.0579640	-2353.2331291	-2353.2096056
7	-2347.8319503	-2348.0343928	-2347.9954317	-2350.9198377	-2351.0305416	-2350.9972428	-2354.7999245	-2354.9656256	-2354.9476732	-2353.0579309	-2353.2328174	-2353.2096129
8	-2347.8292441	-2348.0343275	-2347.9948096	-2350.9194188	-2351.0281880	-2350.9977817	-2354.8232698	-2354.9671533	-2354.9441490	-2353.0747634	-2353.2339469	-2353.2068141
9	-2347.8136171	-2348.0322248	-2347.9921125	-2350.9009306	-2351.0300760	-2350.9967219	-2354.7842628	-2354.9690959	-2354.9462618	-2353.0416014	-2353.2348781	-2353.2077245
10	-2347.8135715	-2348.0294353	-2347.9906003	-2350.9008854	-2351.0229322	-2350.9908890	-2354.7842295	-2354.9719422	-2354.9420517	-2353.0415650	-2353.2363154	-2353.2041888
11	-2347.8104081	-2348.0292589	-2347.9872843	-2350.8980234	-2351.0226544	-2350.9906184	-2354.7975065	-2354.9715980	-2354.9351257	-2353.0507319	-2353.2360132	-2353.1981654
12	-2347.8099245	-2348.0179952	-2347.9854962	-2350.8965022	-2351.0176515	-2350.9860227	-2354.7923612	-2354.9577885	-2354.9373244	-2353.0467520	-2353.2228402	-2353.1993674
13	-2347.8081677	-2348.0160530	-2347.9854784	-2350.8915382	-2351.0169628	-2350.9860115	-2354.7976633	-2354.9707417	-2354.9373196	-2353.0502894	-2353.2320695	-2353.1993593
14	-2347.8058188	-2348.0104733	-2347.9689672	-2350.8963051	-2351.0061528	-2350.9735890	-2354.8017855	-2354.9474121	-2354.9243653	-2353.0527938	-2353.2131774	-2353.1855158
15	-2347.8056454	-2348.0077850	-2347.9686347	-2350.8957907	-2351.0035835	-2350.9671661	-2354.8027903	-2354.9438010	-2354.9232836	-2353.0535041	-2353.2097970	-2353.1846214
16	-2347.8056387	-2348.0077187	-2347.9672320	-2350.8957595	-2351.0034753	-2350.9747331	-2354.8028541	-2354.9437374	-2354.9288342	-2353.0535502	-2353.2097327	-2353.1884337
17	-2347.8021850	-2348.0071358	-2347.9652446	-2350.8898170	-2351.0076454	-2350.9629493	-2354.7930466	-2354.9597975	-2354.9190190	-2353.0453312	-2353.2216321	-2353.1805754
18	-2347.8008877	-2348.0061703	-2347.9652008	-2350.8896177	-2351.0016211	-2350.9629229	-2354.7943064	-2354.9381925	-2354.9189729	-2353.0459517	-2353.2051869	-2353.1805299
19	-2347.7971080	-2348.0048160	-2347.9587382	-2350.8824985	-2351.0049327	-2350.9641306	-2354.7896742	-2354.9585658	-2354.9089691	-2353.0415327	-2353.2201283	-2353.1714114
20	-2347.7964931	-2348.0030396	-2347.9579915	-2350.8854032	-2350.9970994	-2350.9652951	-2354.7842840	-2354.9388421	-2354.9162259	-2353.0373363	-2353.2048915	-2353.1766673
21	-2347.7964645	-2348.0029581	-2347.9529234	-2350.8853885	-2350.9970183	-2350.9575661	-2354.7842891	-2354.9387343	-2354.9091305	-2353.0373330	-2353.2047902	-2353.1700787
22	-2347.7793780	-2347.9964036	-2347.9430161	-2350.8733311	-2350.9894774	-2350.9425414	-2354.7885693	-2354.9423019	-2354.9124636	-2353.0362715	-2353.2058273	-2353.1701017
23	-2347.7769233	-2347.9936290	-2347.9430093	-2350.8724115	-2350.9889285	-2350.9425316	-2354.7868974	-2354.9337286	-2354.9124333	-2353.0344039	-2353.1987037	-2353.1700773
24	-2347.7765656	-2347.9935553	-2347.9409121	-2350.8732363	-2350.9888471	-2350.9413133	-2354.7853477	-2354.9335851	-2354.9111784	-2353.0331522	-2353.1985777	-2353.1686118
25	-2347.7741591	-2347.9810983	-2347.9299439	-2350.8699375	-2350.9909363	-2350.9428623	-2354.7818972	-2354.9577500	-2354.9197255	-2353.0299627	-2353.2135871	-2353.1722801
26	-2347.7741282	-2347.9808107	-2347.9278890	-2350.8699101	-2350.9795677	-2350.9410233	-2354.7818491	-2354.9362752	-2354.9174285	-2353.0299189	-2353.1974090	-2353.1700437
27	-2347.7482840	-2347.9807033	-2347.9278385	-2350.8528681	-2350.9794193	-2350.9409861	-2354.7875989	-2354.9360909	-2354.9173465	-2353.0277702	-2353.1972440	-2353.1699695
28	-2347.7461674	-2347.9802813	-2347.9217630	-2350.8532355	-2350.9781546	-2350.9307947	-2354.7716370	-2354.9368753	-2354.9107679	-2353.0152696	-2353.1977268	-2353.1635167
29	-2347.7461295	-2347.9783772	-2347.9193716	-2350.8532124	-2350.9875699	-2350.9332251	-2354.7716586	-2354.9535499	-2354.9121694	-2353.0152763	-2353.2097567	-2353.1639699
30	-2347.7455576	-2347.9766001	-2347.9182022	-2350.8536428	-2350.9673899	-2350.9320510	-2354.7875597	-2354.9186439	-2354.9087491	-2353.0270592	-2353.1831330	-2353.1611123

Table S25: Computed absolute energies with the SA-CASSCF, CASPT2, tPBE, and tPBE0 methods for the relative energies in Figures S10 and S18 of Co(o-tol)$_4^{2-}$ molecule when using the (13,13) active space.

	SA-CASSCF		CASPT2		tPBE		tPBE0	
No.	Quartet	Doublet	Quartet	Doublet	Quartet	Doublet	Quartet	Doublet
1	-2468.67917357	-2468.60599668	-2471.67914107	-2471.61646211	-2475.66229034	-2475.61173898	-2473.91651115	-2473.86030341
2	-2468.65128763	-2468.60587897	-2471.65085325	-2471.61652917	-2475.63267012	-2475.61165219	-2473.88732450	-2473.86020889
3	-2468.64850296	-2468.60587748	-2471.64765744	-2471.61652738	-2475.62808849	-2475.61165134	-2473.88319211	-2473.86020788
4	-2468.64850035	-2468.60231760	-2471.64765497	-2471.61073218	-2475.62808604	-2475.60778672	-2473.88318962	-2473.85641944
5	-2468.62848521	-2468.60180491	-2471.62916605	-2471.61014797	-2475.60383035	-2475.60744235	-2473.85999407	-2473.85603299
6	-2468.62848379	-2468.59169234	-2471.62916462	-2471.60067112	-2475.60382955	-2475.58275382	-2473.85999311	-2473.83498845
7	-2468.62789027	-2468.58301965	-2471.62957586	-2471.59610565	-2475.60168720	-2475.58715379	-2473.85823797	-2473.83612026
8	-2468.57865370	-2468.58180094	-2471.59502065	-2471.59445450	-2475.60513306	-2475.58939468	-2473.84851322	-2473.83749625
9	-2468.57830324	-2468.58179842	-2471.59378685	-2471.59445137	-2475.59545084	-2475.58939337	-2473.84116394	-2473.83749463
10	-2468.57830030	-2468.57335570	-2471.59378415	-2471.58072342	-2475.59544529	-2475.57559048	-2473.84115904	-2473.82503179
11	-2468.39730326	-2468.57123724	-2471.49263478	-2471.57799146	-2475.44017739	-2475.57155998	-2473.67945886	-2473.82147930
12	-2468.39714496	-2468.57123450	-2471.49593832	-2471.57798845	-2475.44301328	-2475.57155644	-2473.68154620	-2473.82147596
13	-2468.39714211	-2468.57044505	-2471.49658503	-2471.58001575	-2475.44213912	-2475.57990614	-2473.68088987	-2473.82754087
14	-2468.39651989	-2468.56875867	-2471.49070500	-2471.58315585	-2475.44027502	-2475.57767494	-2473.67933624	-2473.82544587
15	-2468.39616126	-2468.56763271	-2471.49005554	-2471.57695097	-2475.44060220	-2475.57490614	-2473.67949197	-2473.82308778
16	-2468.38823630	-2468.56762968	-2471.48776263	-2471.57694792	-2475.43982393	-2475.57490324	-2473.67692702	-2473.82308485
17	-2468.38713937	-2468.56502496	-2471.48769377	-2471.57906601	-2475.44146568	-2475.57375923	-2473.67788410	-2473.82157566
18	-2468.38713766	-2468.54833200	-2471.48769184	-2471.55955415	-2475.44146419	-2475.55833470	-2473.67788256	-2473.80583403
19	-2468.37721418	-2468.54793522	-2471.47542350	-2471.55762554	-2475.42720423	-2475.55770259	-2473.66470672	-2473.80526075
20	-2468.37468537	-2468.54793398	-2471.47254729	-2471.55762518	-2475.42731923	-2475.55770105	-2473.66416077	-2473.80525928
21	-2468.36987613	-2468.54078396	-2471.46970738	-2471.54736020	-2475.42943069	-2475.54637291	-2473.66454205	-2473.79497567
22	-2468.36515365	-2468.54078172	-2471.46225300	-2471.54735634	-2475.42877096	-2475.54636784	-2473.66286663	-2473.79497131
23	-2468.36257505	-2468.53837888	-2471.46142118	-2471.54386800	-2475.42514451	-2475.54412413	-2473.65950215	-2473.79268782
24	-2468.36257440	-2468.52947536	-2471.46142720	-2471.53310183	-2475.42514273	-2475.53722468	-2473.65950065	-2473.78528735
25	-2468.36033701	-2468.52947275	-2471.46104647	-2471.53309853	-2475.42549328	-2475.53722225	-2473.65920421	-2473.78528488
26	-2468.35775008	-2468.52841158	-2471.46338298	-2471.53120200	-2475.43153822	-2475.53538253	-2473.66309119	-2473.78363979
27	-2468.35685719	-2468.51333048	-2471.45695847	-2471.52371399	-2475.41923068	-2475.53497374	-2473.65363731	-2473.77956293
28	-2468.35685454	-2468.51242425	-2471.45693082	-2471.51575458	-2475.41922866	-2475.53248995	-2473.65363513	-2473.77747353
29	-2468.34899255	-2468.50990074	-2471.44726911	-2471.53504932	-2475.41044492	-2475.56559396	-2473.64508183	-2473.80167066
30	-2468.34898914	-2468.50021261	-2471.44727065	-2471.53064503	-2475.41046598	-2475.56892936	-2473.64509677	-2473.80175017

Table S26: Computed absolute energies with the SA-CASSCF, CASPT2, tPBE, and tPBE0 methods for the relative energies in Figures S11 and S19 of Ni(o-tol)$_4^{2-}$ molecule when using the (14,13) active space.

	SA-CASSCF		CASPT2		tPBE		tPBE0	
No.	Triplet	Singlet	Triplet	Singlet	Triplet	Singlet	Triplet	Singlet
1	-2595.90860311	-2595.85346906	-2598.97746637	-2598.93946168	-2602.94031575	-2602.91836788	-2601.18238759	-2601.15214318
2	-2595.88543343	-2595.84310586	-2598.95208014	-2598.92703257	-2602.91023545	-2602.91581356	-2601.15403495	-2601.14763664
3	-2595.88311501	-2595.82336403	-2598.94925318	-2598.90468013	-2602.90764227	-2602.88863888	-2601.15151046	-2601.12232017
4	-2595.87787624	-2595.82024141	-2598.94252221	-2598.90428476	-2602.90573752	-2602.88505620	-2601.14877220	-2601.11885250
5	-2595.86473408	-2595.81990678	-2598.93018752	-2598.90109732	-2602.88360080	-2602.88159083	-2601.12888412	-2601.11616982
6	-2595.85627311	-2595.80043907	-2598.91970254	-2598.88941210	-2602.87493434	-2602.88775208	-2601.12026903	-2601.11592383
7	-2595.84248994	-2595.79554470	-2598.90524643	-2598.88057510	-2602.86760038	-2602.85914575	-2601.11132277	-2601.09324549
8	-2595.80764482	-2595.79004252	-2598.88936057	-2598.87322790	-2602.87448371	-2602.87279393	-2601.10777399	-2601.10210608
9	-2595.79898935	-2595.78078744	-2598.88097566	-2598.86415473	-2602.86339026	-2602.83976701	-2601.09729003	-2601.07502212
10	-2595.79305487	-2595.76983141	-2598.87423122	-2598.84726541	-2602.85535233	-2602.84465403	-2601.08977797	-2601.07594838
11	-2595.63299400	-2595.76944428	-2598.81007390	-2598.84682342	-2602.71287123	-2602.84464088	-2600.94290192	-2601.07584173
12	-2595.63197335	-2595.76162378	-2598.81241925	-2598.83857732	-2602.72796093	-2602.82492747	-2600.95396404	-2601.05910155
13	-2595.62753205	-2595.75225219	-2598.80771420	-2598.82102062	-2602.70700605	-2602.82210778	-2600.93713755	-2601.05464388
14	-2595.61293606	-2595.75115464	-2598.79166782	-2598.82130285	-2602.71353333	-2602.82392662	-2600.93838401	-2601.05573363
15	-2595.61021022	-2595.65758732	-2598.79519406	-2598.77530076	-2602.71953864	-2602.80704270	-2600.94220654	-2601.01967886
16	-2595.60887550	-2595.63412107	-2598.79452740	-2598.82155166	-2602.71621460	-2602.72619010	-2600.93937983	-2600.95317284
17	-2595.60151620	-2595.61028034	-2598.78156690	-2598.79909148	-2602.70868981	-2602.71628632	-2600.93189641	-2600.93978483
18	-2595.59919075	-2595.60571583	-2598.78166268	-2598.79850183	-2602.70300572	-2602.70595510	-2600.92705198	-2600.93089528
19	-2595.59446052	-2595.58683855	-2598.77407719	-2598.76677988	-2602.69436923	-2602.71260791	-2600.91939205	-2600.93116557
20	-2595.58938057	-2595.58618228	-2598.77202108	-2598.76914631	-2602.70304327	-2602.71313475	-2600.92462760	-2600.93139663

5.5 Absolute Energies for Zero-Field Splitting Calculations

The following tables include the energies obtained after the multireference calculations performed for the computation of ZFS parameters.

Summary of spin-states used for the computation of $|D|$:

- Ti(o-tol)$_4^{2-}$ (10,15): 10 triplets and 15 singlets
- V(o-tol)$_4^{-}$ (8,13): 7 triplets and 9 singlets
- Cr(o-tol)$_4$ (10,15): 7 triplets and 9 singlets
- Mo(o-tol)$_4$ (10,15): 7 triplets and 9 singlets
- W(o-tol)$_4$ (10,15): 7 triplets and 9 singlets

Table S27: Computed absolute energies with the SA-CASSCF, CASPT2, tPBE, and tPBE0 methods of Cr(o-tol)$_4$ molecule when using the (10,15) active space to obtain the ZFS.

	SA-CASSCF		CASPT2		tPBE		tPBE0	
No.	Triplet	Singlet	Triplet	Singlet	Triplet	Singlet	Triplet	Singlet
1	-2126.47551516	-2126.41345938	-2129.40514835	-2129.35234568	-2133.28494190	-2133.24672773	-2131.58258522	-2131.53841064
2	-2126.38914476	-2126.41175298	-2129.32794308	-2129.35069372	-2133.20786826	-2133.24589269	-2131.50318739	-2131.53735776
3	-2126.38075988	-2126.37129253	-2129.32278386	-2129.31746819	-2133.19815642	-2133.21740519	-2131.49380729	-2131.50587703
4	-2126.38075970	-2126.34734606	-2129.32278372	-2129.29802713	-2133.19815625	-2133.17720456	-2131.49380711	-2131.46973994
5	-2126.37798317	-2126.34001935	-2129.32103216	-2129.29020764	-2133.20048660	-2133.18415866	-2131.49486074	-2131.47312383
6	-2126.35992718	-2126.33549251	-2129.30499517	-2129.28647136	-2133.18649032	-2133.17473772	-2131.47984954	-2131.46492642
7	-2126.35992686	-2126.33549232	-2129.30499482	-2129.28647125	-2133.18648993	-2133.17473753	-2131.47984916	-2131.46492623
8		-2126.33011658		-2129.28274231		-2133.16461980		-2131.45599400
9		-2126.33011632		-2129.28274196		-2133.16461918		-2131.45599347

Table S28: Computed absolute energies with the SA-CASSCF, CASPT2, tPBE, and tPBE0 methods of Mo(o-tol)$_4$ molecule when using the (10,15) active space to obtain the ZFS.

	SA-CASSCF		CASPT2		tPBE		tPBE0	
No.	Triplet	Singlet	Triplet	Singlet	Triplet	Singlet	Triplet	Singlet
1	-5122.57232500	-5122.53364475	-5125.47208168	-5125.44193198	-5130.29700364	-5130.27705127	-5128.36583398	-5128.34119964
2	-5122.46799929	-5122.52981070	-5125.37456213	-5125.43805069	-5130.20486252	-5130.27493877	-5128.27064671	-5128.33865675
3	-5122.46531335	-5122.49772244	-5125.37560031	-5125.41181070	-5130.20258851	-5130.25973453	-5128.26826972	-5128.31923151
4	-5122.46530941	-5122.44663801	-5125.37559770	-5125.36571622	-5130.20258716	-5130.19600770	-5128.26826772	-5128.25866528
5	-5122.46474139	-5122.43510870	-5125.37641914	-5125.35472987	-5130.20727361	-5130.18676997	-5128.27164056	-5128.24885465
6	-5122.44868565	-5122.43510456	-5125.36060539	-5125.35472603	-5130.19108799	-5130.18676926	-5128.25548741	-5128.24885309
7	-5122.44868130	-5122.43267228	-5125.36060119	-5125.35005424	-5130.19108358	-5130.19154195	-5128.25548301	-5128.25182453
8		-5122.42707509		-5125.34755451		-5130.18348066		-5128.24437927
9		-5122.42707286		-5125.34755130		-5130.18347727		-5128.24437617

Table S29: Computed absolute energies with the SA-CASSCF, CASPT2, tPBE, and tPBE0 methods of W(o-tol)$_4$ molecule when using the (10,15) active space to obtain the ZFS.

	SA-CASSCF		CASPT2		tPBE		tPBE0	
No.	Triplet	Singlet	Triplet	Singlet	Triplet	Singlet	Triplet	Singlet
1	-17204.30179508	-17204.26932854	-17207.19880830	-17207.17232645	-17215.60945088	-17215.59430235	-17212.78253693	-17212.76305890
2	-17204.21498800	-17204.26489458	-17207.11448438	-17207.16812124	-17215.53010149	-17215.59226278	-17212.70132312	-17212.76042073
3	-17204.21489737	-17204.23773251	-17207.11447723	-17207.14443487	-17215.53006733	-17215.58172665	-17212.70127484	-17212.74572812
4	-17204.21465639	-17204.19497693	-17207.11303172	-17207.10147742	-17215.53141264	-17215.52255934	-17212.70222358	-17212.69066374
5	-17204.21197286	-17204.18529444	-17207.11542909	-17207.09290100	-17215.53502442	-17215.51506983	-17212.70426153	-17212.68262598
6	-17204.19709499	-17204.18525849	-17207.10099989	-17207.09296056	-17215.52042655	-17215.51512515	-17212.68959366	-17212.68265849
7	-17204.19706572	-17204.18037866	-17207.10103167	-17207.08675123	-17215.52045991	-17215.51775984	-17212.68961136	-17212.68341455
8		-17204.17674489		-17207.08377144		-17215.51156463		-17212.67785970
9		-17204.17667574		-17207.08379289		-17215.51157476		-17212.67785001

Table S30: Computed absolute energies with the SA-CASSCF, CASPT2, tPBE, and tPBE0 methods of Ti(o-tol)$_4^{2-}$ molecule when using the (10,15) active space to obtain the ZFS.

	SA-CASSCF		CASPT2		tPBE		tPBE0	
No.	Triplet	Singlet	Triplet	Singlet	Triplet	Singlet	Triplet	Singlet
1	-1929.56107248	-1929.53422236	-1932.38520810	-1932.36454340	-1936.26200191	-1936.24403485	-1934.58676955	-1934.56658173
2	-1929.53735394	-1929.52549097	-1932.36273281	-1932.35939393	-1936.23852696	-1936.24388016	-1934.56323371	-1934.56428286
3	-1929.52019277	-1929.50825080	-1932.35082107	-1932.34905714	-1936.22485065	-1936.23415098	-1934.54868618	-1934.55267594
4	-1929.52019271	-1929.49682023	-1932.35082093	-1932.33392515	-1936.22485046	-1936.21935933	-1934.54868602	-1934.53872456
5	-1929.50904801	-1929.48348570	-1932.34930727	-1932.32661958	-1936.22386989	-1936.20457429	-1934.54516442	-1934.52430214
6	-1929.50435387	-1929.48093581	-1932.34255449	-1932.32574379	-1936.21196383	-1936.20305215	-1934.53506134	-1934.52252307
7	-1929.50435383	-1929.48093572	-1932.34255456	-1932.32574366	-1936.21196346	-1936.20305204	-1934.53506105	-1934.52252296
8	-1929.48212891	-1929.47241397	-1932.32278093	-1932.31938699	-1936.20275347	-1936.20112668	-1934.52259733	-1934.51894850
9	-1929.48212884	-1929.47241389	-1932.32278080	-1932.31938687	-1936.20275345	-1936.20112628	-1934.52259730	-1934.51894818
10	-1929.47716644	-1929.46256743	-1932.31699463	-1932.30444599	-1936.19619992	-1936.18558980	-1934.51644155	-1934.50483421
11		-1929.45159284		-1932.29996563		-1936.18156759		-1934.49907390
12		-1929.44534064		-1932.29449528		-1936.18155552		-1934.49750180
13		-1929.44534056		-1932.29449494		-1936.18155543		-1934.49750171
14		-1929.43447537		-1932.28452777		-1936.17093477		-1934.48681992
15		-1929.39042708		-1932.26249793		-1936.16687645		-1934.47276411

Table S31: Computed absolute energies with the SA-CASSCF, CASPT2, tPBE, and tPBE0 methods of $V(o\text{-tol})_4^-$ molecule when using the (8,13) active space to obtain the ZFS.

	SA-CASSCF		CASPT2		tPBE		tPBE0	
No.	Triplet	Singlet	Triplet	Singlet	Triplet	Singlet	Triplet	Singlet
1	-2025.05200481	-2024.99823961	-2027.94025349	-2027.89707284	-2031.81888592	-2031.79496485	-2030.12716564	-2030.09578354
2	-2025.00579861	-2024.99728412	-2027.89287475	-2027.89606643	-2031.77288973	-2031.79448166	-2030.08111695	-2030.09518228
3	-2025.00063901	-2024.96130532	-2027.88754099	-2027.86721940	-2031.76718562	-2031.77601271	-2030.07554897	-2030.07233586
4	-2025.00063573	-2024.95382997	-2027.88753749	-2027.85362702	-2031.76718307	-2031.75451552	-2030.07554624	-2030.05434413
5	-2024.98035598	-2024.94896023	-2027.87488149	-2027.85058297	-2031.74965942	-2031.74310576	-2030.05733356	-2030.04456938
6	-2024.97367743	-2024.94895970	-2027.86715898	-2027.85058333	-2031.74030518	-2031.74310514	-2030.04864824	-2030.04456878
7	-2024.97367382	-2024.94890915	-2027.86715640	-2027.85152663	-2031.74029982	-2031.73635417	-2030.04864332	-2030.03949292
8		-2024.93909397		-2027.84318909		-2031.72906957		-2030.03157567
9		-2024.93909137		-2027.84318455		-2031.72906277		-2030.03156992

6 Active Space Dependency

6.1 Energy Gaps

Table S32: Computed triplet-singlet gaps for the Cr(o-tol)$_4$ complex. The energy values were obtained with SA-CASSCF, CASPT2, tPBE, and tPBE0 methods. The values reported are in eV. Percentage of the dominant configurations for the triplet ground state (T_0) and the lowest singlet state (S_1).

Active Space	SA-CASSCF	CASPT2	tPBE	tPBE0	T_0 (%)	S_1 (%)
(2,5)	1.91	0.98	0.48	0.84	83.42	70.41
(2,10)	1.84	1.02	0.52	0.85	84.10	61.26
(8,8)	1.90	1.32	0.82	1.09	88.99	57.56
(10,15)	1.69	1.44	1.04	1.20	84.17	50.83

Table S33: Computed triplet-singlet gaps for the Mo(o-tol)$_4$ complex. The energy values were obtained with SA-CASSCF, CASPT2, tPBE, and tPBE0 methods. The values reported are in eV. Percentage of the dominant conigurations for the triplet ground state (T_0) and the lowest singlet state (S_1).

Active Space	SA-CASSCF	CASPT2	tPBE	tPBE0	T_0 (%)	S_1 (%)
(2,5)	1.19	0.73	0.28	0.50	63.89	40.61
(2,10)	1.15	0.68	0.30	0.51	92.84	37.88
(8,8)	1.15	0.76	0.42	0.60	96.78	63.60
(10,15)	1.05	0.82	0.54	0.67	90.89	52.95

Table S34: Computed triplet-singlet gaps for the W(o-tol)$_4$ complex. The energy values were obtained with SA-CASSCF, CASPT2, tPBE, and tPBE0 methods. The values reported are in eV. Percentage of the dominant configurations for the triplet ground state (T_0) and the lowest singlet state (S_1).

Active Space	SA-CASSCF	CASPT2	tPBE	tPBE0	T_0 (%)	S_1 (%)
(2,5)	1.04	0.64	0.23	0.43	56.13	30.71
(2,10)	1.01	0.60	0.24	0.43	94.74	38.74
(8,8)	1.00	0.69	0.36	0.52	97.90	61.82
(10,15)	0.88	0.72	0.41	0.55	95.76	54.32

Table S35: Computed triplet-singlet gaps for the Ti(o-tol)$_4^{2-}$ complex. The energy values were obtained with SA-CASSCF, CASPT2, tPBE, and tPBE0 methods. The values reported are in eV. Percentage of the dominant configurations for the triplet ground state (T_0) and the lowest singlet state (S_1).

Active Space	SA-CASSCF	CASPT2	tPBE	tPBE0	T_0 (%)	S_1 (%)
(2,5)	1.12	0.51	0.13	0.38	26.04	71.88
(2,10)	0.87	0.41	0.03	0.24	74.38	63.65
(8,8)	1.17	0.80	0.55	0.70	96.49	56.98
(10,15)	0.73	0.56	0.49	0.55	67.72	71.45

Table S36: Computed triplet-singlet gaps for the V(o-tol)$_4^-$ complex. The energy values were obtained with SA-CASSCF, CASPT2, tPBE, and tPBE0 methods. The values reported are in eV. Percentage of the dominant configurations for the triplet ground state (T_0) and the lowest singlet state (S_1).

Active Space	SA-CASSCF	CASPT2	tPBE	tPBE0	T_0 (%)	S_1 (%)
(2,5)	1.70	1.06	0.47	0.78	57.25	20.38
(2,10)	1.62	1.07	0.48	0.76	74.79	41.16
(8,8)	1.64	1.17	0.63	0.89	97.86	55.56
(8,13)	1.46	1.18	0.65	0.85	96.28	51.86

Table S37: Computed quintet-triplet ($\Delta E_{\text{Q-T}}$) and quintet-triplet ($\Delta E_{\text{Q-S}}$) gaps for the Fe(o-tol)$_4^{2-}$ complex. The energy values were obtained with SA-CASSCF, CASPT2, tPBE, and tPBE0 methods. The values reported are in eV.

Active Space	SA-CASSCF		CASPT2		tPBE		tPBE0	
	$\Delta E_{\text{Q-T}}$	$\Delta E_{\text{Q-S}}$	$\Delta E_{\text{Q-T}}$	$\Delta E_{\text{Q-S}}$	$\Delta E_{\text{Q-T}}$	$\Delta E_{\text{Q-S}}$	$\Delta E_{\text{Q-T}}$	$\Delta E_{\text{Q-S}}$
(6,5)	2.12	3.40	1.73	2.41	1.02	1.72	1.29	2.14
(6,10)	2.01	3.22	1.73	2.55	1.01	1.82	1.26	2.17
(12,8)	2.06	3.36	2.00	3.06	1.29	2.38	1.48	2.62
(12,13)	1.59	2.70	1.53	2.41	0.83	1.67	1.02	1.93

Table S38: Computed quartet-doublet gaps for the Co(o-tol)$_4^{2-}$ complex. The energy values were obtained with SA-CASSCF, CASPT2, tPBE, and tPBE0 methods. The values reported are in eV.

Active Space	SA-CASSCF	CASPT2	tPBE	tPBE0
(7,5)	2.41	1.85	1.53	1.75
(7,10)	2.34	1.95	1.60	1.79
(13,8)	1.95	2.03	1.34	1.49
(13,13)	1.99	1.71	1.38	1.53

Table S39: Computed triplet-singlet gaps for the Ni(o-tol)$_4^{2-}$ complex. The energy values were obtained with SA-CASSCF, CASPT2, tPBE, and tPBE0 methods. The values reported are in eV.

Active Space	SA-CASSCF	CASPT2	tPBE	tPBE0
(8,5)	1.95	1.17	0.61	0.95
(8,10)	1.89	1.41	0.84	1.10
(14,8)	1.96	1.46	0.85	1.12
(14,13)	1.64	1.10	0.67	0.91

6.2 Zero-Field Splitting Parameters

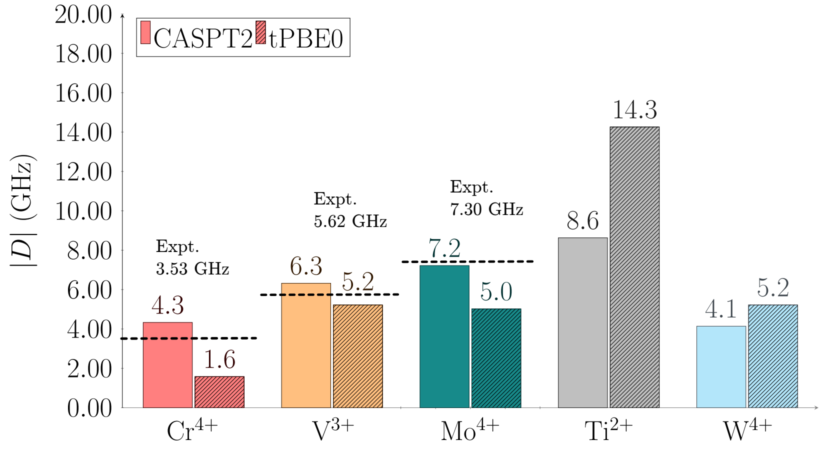

Figure S20: Calculated axial parameter ($|D|$) with the CASPT2 (solid bar)and tPBE0 (striped bar) methods for the Cr(o-tol)$_4$ (red), V(o-tol)$_4^-$ (orange), Mo(o-tol)$_4$ (teal), Ti(o-tol)$_4^{2-}$ (gray), and W(o-tol)$_4$ (cyan) complexes using (2,5) active space. The values are in GHz. Dashed lines correspond to experimental data from references 1 and 2.

Table S40: Computed axial parameter ($|D|$) using the (2,5) active space for Cr(o-tol)$_4$, V(o-tol)$_4^-$, Mo(o-tol)$_4$, Ti(o-tol)$_4^{2-}$, and W(o-tol)$_4$ complexes. The energy values were obtained with SA-CASSCF, CASPT2, tPBE, and tPBE0 methods. The values reported are in GHz.

Complex	SA-CASSCF	CASPT2	tPBE	tPBE0
Cr(o-tol)$_4$	10.05	4.33	1.26	1.58
V(o-tol)$_4^-$	8.11	6.32	2.95	5.22
Mo(o-tol)$_4$	10.72	7.22	12.74	5.02
Ti(o-tol)$_4^{2-}$	8.98	8.63	11.90	14.26
W(o-tol)$_4$	169.21	4.14	120.73	5.22

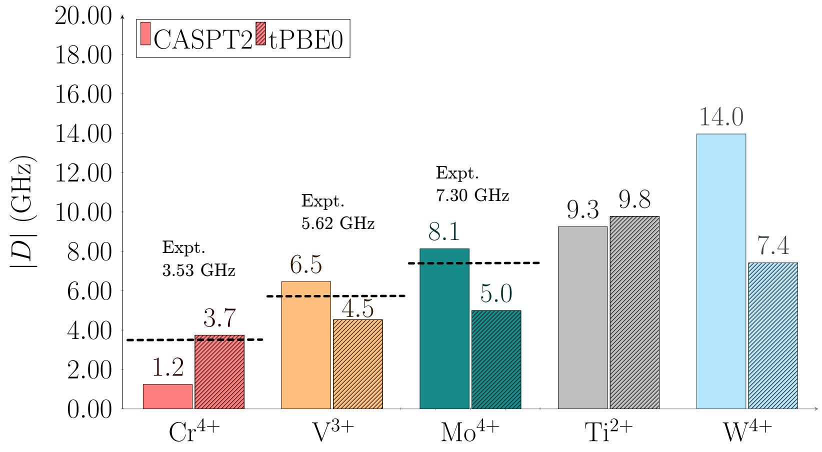

Figure S21: Calculated axial parameter ($|D|$) with the CASPT2 (solid bar)and tPBE0 (striped bar) methods for the Cr(o-tol)$_4$ (red), V(o-tol)$_4^-$ (orange), Mo(o-tol)$_4$ (teal), Ti(o-tol)$_4^{2-}$ (gray), and W(o-tol)$_4$ (cyan) complexes using (2,10) active space. The values are in GHz. Dashed lines correspond to experimental data from references 1 and 2.

Table S41: Computed axial parameter ($|D|$) using the (2,10) active space for Cr(o-tol)$_4$, V(o-tol)$_4^-$, Mo(o-tol)$_4$, Ti(o-tol)$_4^{2-}$, and W(o-tol)$_4$ complexes. The energy values were obtained with SA-CASSCF, CASPT2, tPBE, and tPBE0 methods. The values reported are in GHz.

Complex	SA-CASSCF	CASPT2	tPBE	tPBE0
Cr(o-tol)$_4$	9.23	1.24	1.43	3.74
V(o-tol)$_4^-$	7.90	6.46	2.59	4.53
Mo(o-tol)$_4$	10.82	8.13	11.93	4.99
Ti(o-tol)$_4^{2-}$	10.63	9.25	5.83	9.77
W(o-tol)$_4$	201.07	13.96	95.64	7.42

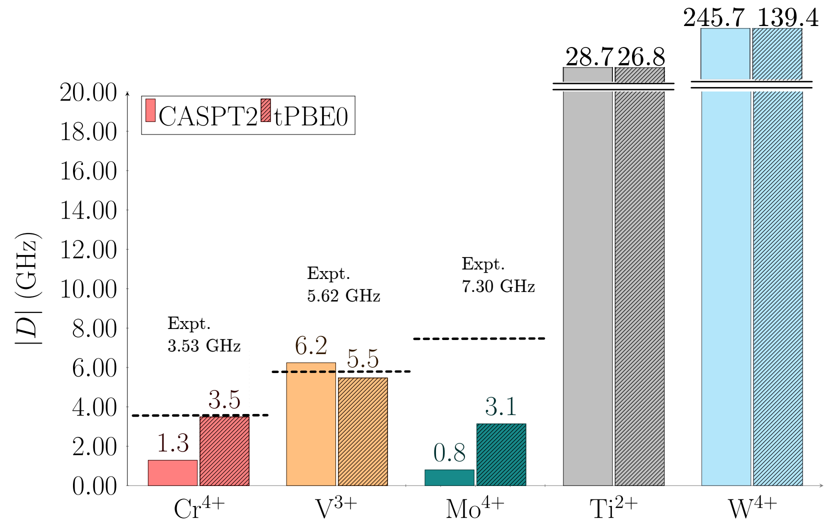

Figure S22: Calculated axial parameter ($|D|$) with the CASPT2 (solid bar)and tPBE0 (striped bar) methods for the Cr(o-tol)$_4$ (red), V(o-tol)$_4^-$ (orange), Mo(o-tol)$_4$ (teal), Ti(o-tol)$_4^{2-}$ (gray), and W(o-tol)$_4$ (cyan) complexes using (8,8) active space. The values are in GHz. Dashed lines correspond to experimental data from references 1 and 2.

Table S42: Computed axial parameter ($|D|$) using the (8,8) active space for Cr(o-tol)$_4$, V(o-tol)$_4^-$, Mo(o-tol)$_4$, Ti(o-tol)$_4^{2-}$, and W(o-tol)$_4$ complexes. The energy values were obtained with SA-CASSCF, CASPT2, tPBE, and tPBE0 methods. The values reported are in GHz.

Complex	SA-CASSCF	CASPT2	tPBE	tPBE0
Cr(o-tol)$_4$	5.94	1.29	1.49	3.49
V(o-tol)$_4^-$	7.63	6.24	3.23	5.47
Mo(o-tol)$_4$	8.03	0.80	11.32	3.14
Ti(o-tol)$_4^{2-}$	4.32	28.72	37.54	26.77
W(o-tol)$_4$	201.36	185.62	12.29	102.96

Table S43: Computed axial parameter ($|D|$) using the (8,13) active space for V(o-tol)$_4^-$ and (10,15) active space for Cr(o-tol)$_4$, Mo(o-tol)$_4$, Ti(o-tol)$_4^{2-}$, and W(o-tol)$_4$ complexes. The energy values were obtained with SA-CASSCF, CASPT2, tPBE, and tPBE0 methods. The values reported are in GHz.

Complex	SA-CASSCF	CASPT2	tPBE	tPBE0
Cr(o-tol)$_4$	4.06	2.72	1.35	2.39
V(o-tol)$_4^-$	6.96	6.87	0.34	2.95
Mo(o-tol)$_4$	14.01	6.82	1.21	4.09
Ti(o-tol)$_4^{2-}$	17.21	15.11	12.21	13.82
W(o-tol)$_4$	219.85	145.50	68.10	124.04

6.3 Absolute Electronic Energies

6.3.1 Cr(o-tol)$_4$Complex

Table S44: Computed absolute energies with the SA-CASSCF, CASPT2, tPBE, and tPBE0 methods for the Cr(o-tol)$_4$ complex when using the (2,5) active space.

	SA-CASSCF		CASPT2		tPBE		tPBE0	
No.	Triplet	Singlet	Triplet	Singlet	Triplet	Singlet	Triplet	Singlet
1	-2126.30624935	-2126.23614609	-2129.41767400	-2129.38157702	-2133.29414582	-2133.27635417	-2131.54717170	-2131.51630215
2	-2126.24391589	-2126.23480260	-2129.32575277	-2129.37993967	-2133.20650494	-2133.27707479	-2131.46585768	-2131.51650674
3	-2126.23827510	-2126.18619333	-2129.31814130	-2129.30027388	-2133.19944397	-2133.18454955	-2131.45915175	-2131.43496050
4	-2126.23827495	-2126.18607161	-2129.31814068	-2129.29578829	-2133.19944335	-2133.19589975	-2131.45915125	-2131.44344272
5	-2126.21845775	-2126.18441698	-2129.31059048	-2129.33659313	-2133.18614053	-2133.24727830	-2131.44421984	-2131.48156297
6	-2126.20091935	-2126.18187156	-2129.28355144	-2129.29073043	-2133.16431175	-2133.18462200	-2131.42346365	-2131.43393439
7	-2126.20091891	-2126.18187144	-2129.28354961	-2129.29072897	-2133.16430994	-2133.18462043	-2131.42346218	-2131.43393318
8	-2126.15193413	-2126.16489564	-2129.21796830	-2129.27412270	-2133.10475889	-2133.16260354	-2131.36655270	-2131.41317657
9	-2126.15193383	-2126.16489526	-2129.21796711	-2129.27412061	-2133.10475757	-2133.16260136	-2131.36655164	-2131.41317484
10	-2126.13988435	-2126.13439739	-2129.19667564	-2129.21544694	-2133.08854322	-2133.11027670	-2131.35137850	-2131.36630687
11		-2126.11458258		-2129.18927648		-2133.08099412		-2131.33939124
12		-2126.10913139		-2129.19617145		-2133.08987593		-2131.34468980
13		-2126.10913042		-2129.19617378		-2133.08987368		-2131.34468787
14		-2126.09316917		-2129.18010502		-2133.07566352		-2131.33003993
15		-2126.02876163		-2129.16257970		-2133.10295594		-2131.33440736

Table S45: Computed absolute energies with the SA-CASSCF, CASPT2, tPBE, and tPBE0 methods for the Cr(o-tol)$_4$ complex when using the (2,10) active space.

	SA-CASSCF		CASPT2		tPBE		tPBE0	
No.	Triplet	Singlet	Triplet	Singlet	Triplet	Singlet	Triplet	Singlet
1	-2126.31047778	-2126.24297430	-2129.41808546	-2129.38045551	-2133.29316590	-2133.27408198	-2131.54749387	-2131.51630506
2	-2126.24752839	-2126.24156349	-2129.32437172	-2129.37794840	-2133.20497655	-2133.27367894	-2131.46561451	-2131.51565008
3	-2126.24189988	-2126.19409480	-2129.32093170	-2129.33588959	-2133.19322891	-2133.24209559	-2131.45539665	-2131.48009539
4	-2126.24189964	-2126.19344450	-2129.32093159	-2129.30266740	-2133.19322861	-2133.18850714	-2131.45539637	-2131.43974148
5	-2126.22382039	-2126.19249984	-2129.31098551	-2129.29330827	-2133.18478333	-2133.19348590	-2131.44454260	-2131.44323939
6	-2126.20560174	-2126.18859698	-2129.28172786	-2129.28918377	-2133.16316725	-2133.18122479	-2131.42377587	-2131.43306784
7	-2126.20560130	-2126.18859677	-2129.28172697	-2129.28918212	-2133.16316693	-2133.18122429	-2131.42377552	-2131.43306741
8	-2126.15619785	-2126.17210725	-2129.21840991	-2129.27265856	-2133.10799913	-2133.15998060	-2131.37004881	-2131.41301226
9	-2126.15619772	-2126.17210688	-2129.21840976	-2129.27265761	-2133.10799878	-2133.15997920	-2131.37004852	-2131.41301112
10	-2126.14375943	-2126.13991628	-2129.19499208	-2129.21311510	-2133.08534418	-2133.10664531	-2131.34994799	-2131.36496305
11		-2126.11946414		-2129.18701579		-2133.07865934		-2131.33886054
12		-2126.11519283		-2129.19413148		-2133.09017065		-2131.34642620
13		-2126.11519224		-2129.19413060		-2133.09016943		-2131.34642513
14		-2126.09909505		-2129.17794082		-2133.07280097		-2131.32937449
15		-2126.04396023		-2129.15669759		-2133.08690018		-2131.32616519

Table S46: Computed absolute energies with the SA-CASSCF, CASPT2, tPBE, and tPBE0 methods for the Cr(o-tol)$_4$ complex when using the (8,8) active space.

	SA-CASSCF		CASPT2		tPBE		tPBE0	
No.	Triplet	Singlet	Triplet	Singlet	Triplet	Singlet	Triplet	Singlet
1	-2126.39283017	-2126.32312728	-2129.40002333	-2129.35163756	-2133.29073235	-2133.26069317	-2131.56625681	-2131.52630170
2	-2126.31226633	-2126.32180143	-2129.31996490	-2129.34971401	-2133.21279139	-2133.26111149	-2131.48766013	-2131.52628398
3	-2126.30524081	-2126.27560169	-2129.31725876	-2129.30948942	-2133.20273259	-2133.22958347	-2131.47835965	-2131.49108803
4	-2126.30524062	-2126.25793795	-2129.31725868	-2129.29067318	-2133.20273247	-2133.18592383	-2131.47835951	-2131.45392736
5	-2126.29487008	-2126.25447415	-2129.31163303	-2129.28522386	-2133.20250257	-2133.19711103	-2131.47559445	-2131.46145181
6	-2126.27551209	-2126.24903450	-2129.29347114	-2129.28128005	-2133.18768219	-2133.18644678	-2131.45963967	-2131.45209371
7	-2126.27551195	-2126.24903413	-2129.29347107	-2129.28127966	-2133.18768213	-2133.18644630	-2131.45963959	-2131.45209326
8		-2126.23869904		-2129.27294908		-2133.17071092		-2131.43770795
9		-2126.23869853		-2129.27294845		-2133.17071017		-2131.43770726

6.3.2 Mo(o-tol)$_4$Complex

Table S47: Computed absolute energies with the SA-CASSCF, CASPT2, tPBE, and tPBE0 methods for the Mo(o-tol)$_4$ complex when using the (2,5) active space.

	SA-CASSCF		CASPT2		tPBE		tPBE0	
No.	Triplet	Singlet	Triplet	Singlet	Triplet	Singlet	Triplet	Singlet
1	-5122.47095431	-5122.42719758	-5125.48079269	-5125.45384713	-5130.31301118	-5130.30290423	-5128.35249696	-5128.33397757
2	-5122.38010025	-5122.42375388	-5125.37679076	-5125.45035629	-5130.21341881	-5130.30194237	-5128.25508917	-5128.33239525
3	-5122.38009503	-5122.38678537	-5125.37679412	-5125.41835129	-5130.21341467	-5130.28765131	-5128.25508476	-5128.31243483
4	-5122.37996090	-5122.35315874	-5125.37228680	-5125.36550296	-5130.21512704	-5130.21010975	-5128.25633551	-5128.24587200
5	-5122.37192381	-5122.34322961	-5125.37202310	-5125.35608043	-5130.21430296	-5130.20670511	-5128.25370817	-5128.24083624
6	-5122.35368005	-5122.34322423	-5125.35224673	-5125.35607654	-5130.19617664	-5130.20670346	-5128.23555249	-5128.24083365
7	-5122.35367399	-5122.34143394	-5125.35223925	-5125.35219376	-5130.19616891	-5130.20914552	-5128.23554518	-5128.24221763
8	-5122.28427546	-5122.33382533	-5125.26755218	-5125.34481709	-5130.11188844	-5130.19114248	-5128.15498520	-5128.22681319
9	-5122.28426643	-5122.33381911	-5125.26755313	-5125.34480453	-5130.11188115	-5130.19113596	-5128.15497747	-5128.22680675
10	-5122.27436034	-5122.27674343	-5125.25918189	-5125.27034009	-5130.10627447	-5130.12116640	-5128.14829594	-5128.16006066
11		-5122.26285903		-5125.25810123		-5130.10757939		-5128.14639930
12		-5122.24678080		-5125.25175329		-5130.10759554		-5128.14239186
13		-5122.24677453		-5125.25199221		-5130.10758978		-5128.14238597
14		-5122.23632315		-5125.24118825		-5130.10130151		-5128.13505692
15		-5122.20387534		-5125.22869522		-5130.11075929		-5128.13403830

Table S48: Computed absolute energies with the SA-CASSCF, CASPT2, tPBE, and tPBE0 methods for the Mo(o-tol)$_4$ complex when using the (2,10) active space.

	SA-CASSCF		CASPT2		tPBE		tPBE0	
No.	Triplet	Singlet	Triplet	Singlet	Triplet	Singlet	Triplet	Singlet
1	-5122.47315347	-5122.43075908	-5125.47959477	-5125.45467733	-5130.31355106	-5130.30269889	-5128.35345166	-5128.33471394
2	-5122.38197876	-5122.42740667	-5125.37853012	-5125.45029085	-5130.21318928	-5130.30136041	-5128.25538665	-5128.33287198
3	-5122.38197405	-5122.39344413	-5125.37852706	-5125.41988095	-5130.21318562	-5130.28680994	-5128.25538273	-5128.31346849
4	-5122.38184027	-5122.35761822	-5125.37280977	-5125.36767141	-5130.21448182	-5130.21120113	-5128.25632143	-5128.24780540
5	-5122.37504959	-5122.34758549	-5125.37395132	-5125.35720767	-5130.21496522	-5130.20654628	-5128.25498631	-5128.24180608
6	-5122.35665116	-5122.34758016	-5125.35349929	-5125.35720537	-5130.19529058	-5130.20654566	-5128.23563073	-5128.24180429
7	-5122.35664590	-5122.34552988	-5125.35349149	-5125.35111081	-5130.19528299	-5130.20772810	-5128.23562372	-5128.24217855
8	-5122.28623907	-5122.33821435	-5125.26791181	-5125.34473521	-5130.11068996	-5130.19065479	-5128.15457724	-5128.22754468
9	-5122.28623059	-5122.33820811	-5125.26790820	-5125.34472587	-5130.11068328	-5130.19064949	-5128.15457011	-5128.22753915
10	-5122.27638412	-5122.27909025	-5125.25827457	-5125.27071734	-5130.10497898	-5130.12064225	-5128.14783027	-5128.16025425
11		-5122.26511470		-5125.25864036		-5130.10689049		-5128.14644654
12		-5122.25117065		-5125.25073943		-5130.10552276		-5128.14193473
13		-5122.25116475		-5125.25074869		-5130.10551510		-5128.14192751
14		-5122.24080049		-5125.24008267		-5130.09917043		-5128.13457795
15		-5122.21455873		-5125.22661173		-5130.10239682		-5128.13043730

Table S49: Computed absolute energies with the SA-CASSCF, CASPT2, tPBE, and tPBE0 methods for the Mo(o-tol)$_4$ complex when using the (8,8) active space.

	SA-CASSCF		CASPT2		tPBE		tPBE0	
No.	Triplet	Singlet	Triplet	Singlet	Triplet	Singlet	Triplet	Singlet
1	-5122.49778580	-5122.45535200	-5125.46883993	-5125.44080257	-5130.30694133	-5130.29149614	-5128.35465245	-5128.33246011
2	-5122.39866650	-5122.45131556	-5125.37447894	-5125.43700025	-5130.21331829	-5130.29131078	-5128.25965534	-5128.33131198
3	-5122.39866080	-5122.41640010	-5125.37447552	-5125.40789913	-5130.21331561	-5130.27431796	-5128.25965191	-5128.30983850
4	-5122.39839483	-5122.37223423	-5125.37254160	-5125.36389670	-5130.21651185	-5130.20874868	-5128.26198260	-5128.24962007
5	-5122.39309032	-5122.36165061	-5125.37349103	-5125.35453364	-5130.21670266	-5130.20584479	-5128.26079958	-5128.24479625
6	-5122.37520644	-5122.36164494	-5125.35750984	-5125.35453209	-5130.20192130	-5130.20584524	-5128.24524259	-5128.24479517
7	-5122.37520060	-5122.35998562	-5125.35750455	-5125.35172728	-5130.20191522	-5130.20952825	-5128.24523657	-5128.24714259
8		-5122.35320047		-5125.34748262		-5130.19482424		-5128.23441830
9		-5122.35319450		-5125.34747884		-5130.19481895		-5128.23441284

6.3.3 W(o-tol)$_4$Complex

Table S50: Computed absolute energies with the SA-CASSCF, CASPT2, tPBE, and tPBE0 methods for the W(o-tol)$_4$ complex when using the (2,5) active space.

	SA-CASSCF		CASPT2		tPBE		tPBE0	
No.	Triplet	Singlet	Triplet	Singlet	Triplet	Singlet	Triplet	Singlet
1	-17204.25781351	-17204.21904708	-17207.20022358	-17207.17567348	-17215.61416345	-17215.60569174	-17212.77507597	-17212.75903058
2	-17204.17331501	-17204.21447094	-17207.10851957	-17207.17134643	-17215.52766349	-17215.60444291	-17212.68907637	-17212.75694992
3	-17204.17329369	-17204.18233924	-17207.10851801	-17207.14303111	-17215.52765241	-17215.59337429	-17212.68906273	-17212.74061553
4	-17204.17202038	-17204.14805246	-17207.10503791	-17207.09660081	-17215.52964719	-17215.52526033	-17212.69024049	-17212.68095836
5	-17204.16577870	-17204.13820272	-17207.10460776	-17207.08886212	-17215.53003307	-17215.52230215	-17212.68896948	-17212.67627729
6	-17204.14881368	-17204.13817721	-17207.08753360	-17207.08885350	-17215.51355487	-17215.52230866	-17212.67236957	-17212.67627580
7	-17204.14879518	-17204.13371282	-17207.08753295	-17207.08358149	-17215.51353793	-17215.52435048	-17212.67235224	-17212.67669107
8	-17204.08555784	-17204.12951412	-17207.01436138	-17207.07957260	-17215.44178143	-17215.51261618	-17212.60272553	-17212.66684067
9	-17204.08551618	-17204.12950694	-17207.01432674	-17207.07955458	-17215.44176189	-17215.51258346	-17212.60270046	-17212.66681433
10	-17204.07719765	-17204.07836009	-17207.00767911	-17207.01663409	-17215.43765060	-17215.45075459	-17212.59753736	-17212.60765597
11		-17204.06693316		-17207.00647808		-17215.43957052		-17212.59641118
12		-17204.04631977		-17206.99489516		-17215.43873947		-17212.59063455
13		-17204.04630607		-17206.99494617		-17215.43873907		-17212.59063082
14		-17204.03725542		-17206.98556800		-17215.43231715		-17212.58355172
15		-17204.00606036		-17206.97208534		-17215.44097668		-17212.58224760

Table S51: Computed absolute energies with the SA-CASSCF, CASPT2, tPBE, and tPBE0 methods for the W(o-tol)$_4$ complex when using the (2,10) active space.

	SA-CASSCF		CASPT2		tPBE		tPBE0	
No.	Triplet	Singlet	Triplet	Singlet	Triplet	Singlet	Triplet	Singlet
1	-17204.25967576	-17204.22225549	-17207.19923325	-17207.17609909	-17215.61548421	-17215.60648230	-17212.77653210	-17212.76042560
2	-17204.17495059	-17204.21801930	-17207.10983175	-17207.17113379	-17215.52801035	-17215.60524989	-17212.68974541	-17212.75844224
3	-17204.17492905	-17204.18883727	-17207.10982216	-17207.14425749	-17215.52800036	-17215.59449329	-17212.68973253	-17212.74307929
4	-17204.17370638	-17204.15245747	-17207.10500074	-17207.09875202	-17215.52897412	-17215.52736390	-17212.69015719	-17212.68363729
5	-17204.16885565	-17204.14256759	-17207.10672822	-17207.09008830	-17215.53203130	-17215.52283969	-17212.69123739	-17212.67777167
6	-17204.15171008	-17204.14254224	-17207.08814750	-17207.09007943	-17215.51331350	-17215.52284922	-17212.67291265	-17212.67777248
7	-17204.15169179	-17204.13793927	-17207.08813763	-17207.08218347	-17215.51329612	-17215.52244818	-17212.67289504	-17212.67632095
8	-17204.08760244	-17204.13384859	-17207.01442911	-17207.07938227	-17215.44025318	-17215.51140431	-17212.60209050	-17212.66701538
9	-17204.08756001	-17204.13383936	-17207.01439642	-17207.07936510	-17215.44023463	-17215.51137543	-17212.60206598	-17212.66699141
10	-17204.07933028	-17204.08054211	-17207.00670555	-17207.01722393	-17215.43596914	-17215.45035417	-17212.59680943	-17212.60790116
11		-17204.06912822		-17207.00703657		-17215.43877366		-17212.59636230
12		-17204.05095540		-17206.99333038		-17215.43543249		-17212.58931322
13		-17204.05094326		-17206.99334615		-17215.43544111		-17212.58931665
14		-17204.04183774		-17206.98435369		-17215.42949000		-17212.58257694
15		-17204.01657563		-17206.96975081		-17215.43340608		-17212.57919847

Table S52: Computed absolute energies with the SA-CASSCF, CASPT2, tPBE, and tPBE0 methods for the W(o-tol)$_4$ complex when using the (8,8) active space.

	SA-CASSCF		CASPT2		tPBE		tPBE0	
No.	Triplet	Singlet	Triplet	Singlet	Triplet	Singlet	Triplet	Singlet
1	-17204.27601110	-17204.23883388	-17207.18442988	-17207.15853693	-17215.60777713	-17215.59440294	-17212.77483562	-17212.75551068
2	-17204.18532289	-17204.23334516	-17207.09737108	-17207.15427607	-17215.52282197	-17215.59421946	-17212.68844720	-17212.75400089
3	-17204.18530068	-17204.20186107	-17207.09734763	-17207.12813104	-17215.52280853	-17215.58098399	-17212.68843157	-17212.73620326
4	-17204.18423429	-17204.15990286	-17207.09720086	-17207.08395008	-17215.52763978	-17215.51697471	-17212.69178841	-17212.67770675
5	-17204.17892788	-17204.14974634	-17207.09610635	-17207.07525712	-17215.52609599	-17215.51413859	-17212.68930396	-17212.67304053
6	-17204.16233739	-17204.14972708	-17207.08290156	-17207.07522683	-17215.51326072	-17215.51411115	-17212.67552989	-17212.67301513
7	-17204.16232073	-17204.14643344	-17207.08288835	-17207.07403657	-17215.51324672	-17215.51995254	-17212.67551522	-17212.67657277
8		-17204.14173161		-17207.07067605		-17215.50652858		-17212.66532934
9		-17204.14169613		-17207.07063471		-17215.50645359		-17212.66526423

6.3.4 V(o-tol)$_4^-$ Complex

Table S53: Computed absolute energies with the SA-CASSCF, CASPT2, tPBE, and tPBE0 methods for the V(o-tol)$_4^-$ complex when using the (2,5) active space.

	SA-CASSCF		CASPT2		tPBE		tPBE0	
No.	Triplet	Singlet	Triplet	Singlet	Triplet	Singlet	Triplet	Singlet
1	-2025.01428063	-2024.95196111	-2027.93360146	-2027.89447567	-2031.81621252	-2031.79878126	-2030.11572955	-2030.08707622
2	-2024.97109524	-2024.95071759	-2027.88502307	-2027.89454200	-2031.76624205	-2031.80005021	-2030.06745535	-2030.08771706
3	-2024.96683916	-2024.91199892	-2027.87966467	-2027.84885058	-2031.75970495	-2031.75208081	-2030.06148850	-2030.04206034
4	-2024.96683897	-2024.91011933	-2027.87966449	-2027.86032646	-2031.75970473	-2031.77880275	-2030.06148829	-2030.06163190
5	-2024.94379239	-2024.90871430	-2027.86407360	-2027.84405327	-2031.73506875	-2031.74439795	-2030.03724966	-2030.03547704
6	-2024.93896122	-2024.90871416	-2027.85298510	-2027.84405329	-2031.73127542	-2031.74439779	-2030.03319687	-2030.03547688
7	-2024.93896105	-2024.90397114	-2027.85298487	-2027.83986827	-2031.73127525	-2031.72890960	-2030.03319670	-2030.02267499
8	-2024.89590319	-2024.89250341	-2027.81920382	-2027.82919528	-2031.71555429	-2031.71953468	-2030.01064152	-2030.01277686
9	-2024.89590318	-2024.89250320	-2027.81920374	-2027.82919498	-2031.71555417	-2031.71953440	-2030.01064142	-2030.01277660
10	-2024.89430963	-2024.87378941	-2027.81316951	-2027.80245823	-2031.71283056	-2031.70357311	-2030.00820033	-2029.99612719
11		-2024.86354306		-2027.78924052		-2031.68922973		-2029.98280806
12		-2024.85813251		-2027.78938476		-2031.69431462		-2029.98526909
13		-2024.85813233		-2027.78939980		-2031.69431439		-2029.98526888
14		-2024.85023432		-2027.78020542		-2031.68516860		-2029.97643503
15		-2024.76792655		-2027.73599903		-2031.70091911		-2029.96767097

Table S54: Computed absolute energies with the SA-CASSCF, CASPT2, tPBE, and tPBE0 methods for the $V(o\text{-tol})_4^-$ complex when using the (2,10) active space.

	SA-CASSCF		CASPT2		tPBE		tPBE0	
No.	Triplet	Singlet	Triplet	Singlet	Triplet	Singlet	Triplet	Singlet
1	-2025.01826830	-2024.95890167	-2027.93295644	-2027.89371807	-2031.81434295	-2031.79686236	-2030.11532429	-2030.08737219
2	-2024.97503115	-2024.95778032	-2027.88403168	-2027.89306344	-2031.76417295	-2031.79693151	-2030.06688750	-2030.08714371
3	-2024.97070638	-2024.91944228	-2027.87844130	-2027.86060075	-2031.75851183	-2031.77575173	-2030.06156047	-2030.06167437
4	-2024.97070624	-2024.91932728	-2027.87844078	-2027.84688369	-2031.75851142	-2031.74875190	-2030.06156013	-2030.04139575
5	-2024.94884364	-2024.91583298	-2027.86523388	-2027.84214457	-2031.73319074	-2031.74220032	-2030.03710397	-2030.03560849
6	-2024.94346681	-2024.91583283	-2027.85284382	-2027.84214682	-2031.72963986	-2031.74220012	-2030.03309660	-2030.03560830
7	-2024.94346630	-2024.91207891	-2027.85284316	-2027.84047153	-2031.72963938	-2031.72665602	-2030.03309611	-2030.02301174
8	-2024.90179058	-2024.90072972	-2027.81752947	-2027.82975829	-2031.70596585	-2031.71744634	-2030.00492203	-2030.01326719
9	-2024.90178994	-2024.90072947	-2027.81752833	-2027.82975907	-2031.70596459	-2031.71744627	-2030.00492093	-2030.01326707
10	-2024.89962134	-2024.88083138	-2027.81387778	-2027.80274678	-2031.70854428	-2031.70026057	-2030.00631355	-2029.99540327
11		-2024.87028137		-2027.78955576		-2031.68649422		-2029.98244101
12		-2024.86602314		-2027.78858223		-2031.69174867		-2029.98531729
13		-2024.86602285		-2027.78858236		-2031.69174848		-2029.98531707
14		-2024.85809008		-2027.77922232		-2031.68138994		-2029.97556498
15		-2024.78873256		-2027.73546176		-2031.68464802		-2029.96066916

Table S55: Computed absolute energies with the SA-CASSCF, CASPT2, tPBE, and tPBE0 methods for the V(o-tol)$_4^-$ complex when using the (8,8) active space.

	SA-CASSCF		CASPT2		tPBE		tPBE0	
No.	Triplet	Singlet	Triplet	Singlet	Triplet	Singlet	Triplet	Singlet
1	-2025.03011643	-2024.96977105	-2027.93571277	-2027.89264443	-2031.82240985	-2031.79911074	-2030.12433650	-2030.09177582
2	-2024.98358708	-2024.96870007	-2027.88903782	-2027.89198584	-2031.77538484	-2031.80006985	-2030.07743540	-2030.09222741
3	-2024.97872334	-2024.92932564	-2027.88444910	-2027.86070400	-2031.76867497	-2031.77959686	-2030.07118706	-2030.06702906
4	-2024.97872314	-2024.92468303	-2027.88444895	-2027.85047724	-2031.76867479	-2031.75871977	-2030.07118688	-2030.05021059
5	-2024.95667829	-2024.92078733	-2027.87070060	-2027.84665198	-2031.74976624	-2031.74989477	-2030.05149425	-2030.04261791
6	-2024.94996404	-2024.92078702	-2027.86164026	-2027.84665180	-2031.74448456	-2031.74989501	-2030.04585443	-2030.04261801
7	-2024.94996386	-2024.91883692	-2027.86164003	-2027.84454696	-2031.74448438	-2031.73759605	-2030.04585425	-2030.03290627
8		-2024.90751580		-2027.83783413		-2031.73950682		-2030.03150907
9		-2024.90751506		-2027.83783296		-2031.73950522		-2030.03150768

6.3.5 Ti(o-tol)$_4^{2-}$ Complex

Table S56: Computed absolute energies with the SA-CASSCF, CASPT2, tPBE, and tPBE0 methods for the Ti(o-tol)$_4^{2-}$ complex when using the (2,5) active space.

	SA-CASSCF		CASPT2		tPBE		tPBE0	
No.	Triplet	Singlet	Triplet	Singlet	Triplet	Singlet	Triplet	Singlet
1	-1929.50041525	-1929.45750872	-1932.37205812	-1932.35194936	-1936.25242554	-1936.24507187	-1934.56442297	-1934.54818108
2	-1929.47389194	-1929.45483303	-1932.34883341	-1932.34994960	-1936.22998380	-1936.24261968	-1934.54096084	-1934.54567302
3	-1929.46669264	-1929.42865738	-1932.34200050	-1932.32898484	-1936.21185476	-1936.22888741	-1934.52556423	-1934.52882990
4	-1929.46669261	-1929.42663210	-1932.34200049	-1932.32649672	-1936.21185470	-1936.22241669	-1934.52556418	-1934.52347054
5	-1929.44960655	-1929.41954829	-1932.33010639	-1932.31890264	-1936.20092865	-1936.20811950	-1934.51309813	-1934.51097670
6	-1929.44791290	-1929.41954825	-1932.32703718	-1932.31893242	-1936.20227073	-1936.20811945	-1934.51368127	-1934.51097665
7	-1929.44791285	-1929.41811778	-1932.32697384	-1932.31305517	-1936.20227068	-1936.19680688	-1934.51368122	-1934.50213461
8	-1929.42080477	-1929.41527673	-1932.30329727	-1932.31080853	-1936.19147904	-1936.19376377	-1934.49881047	-1934.49914201
9	-1929.42080475	-1929.41527671	-1932.30329726	-1932.31107223	-1936.19147900	-1936.19376380	-1934.49881044	-1934.49914203
10	-1929.41915417	-1929.40556658	-1932.29926996	-1932.29941199	-1936.19188158	-1936.19178391	-1934.49869973	-1934.49522958
11		-1929.39676958		-1932.28959680		-1936.18081937		-1934.48480692
12		-1929.38652123		-1932.28670229		-1936.18029388		-1934.48185072
13		-1929.38652121		-1932.28670216		-1936.18029385		-1934.48185069
14		-1929.38002529		-1932.27630422		-1936.17420661		-1934.47566128
15		-1929.31815906		-1932.24960191		-1936.18351581		-1934.46717662

Table S57: Computed absolute energies with the SA-CASSCF, CASPT2, tPBE, and tPBE0 methods for the $Ti(o\text{-tol})_4^{2-}$ complex when using the (2,10) active space.

	SA-CASSCF		CASPT2		tPBE		tPBE0	
No.	Triplet	Singlet	Triplet	Singlet	Triplet	Singlet	Triplet	Singlet
1	-1929.50718353	-1929.47203876	-1932.37226889	-1932.35409808	-1936.25425309	-1936.25017876	-1934.56748570	-1934.55564376
2	-1929.48164926	-1929.46866455	-1932.34794901	-1932.35103638	-1936.23038736	-1936.24873470	-1934.54320284	-1934.55371716
3	-1929.47336851	-1929.45100045	-1932.34013201	-1932.33580331	-1936.21599607	-1936.23522937	-1934.53033918	-1934.53917214
4	-1929.47336846	-1929.44361514	-1932.34013190	-1932.32508417	-1936.21599598	-1936.22401847	-1934.53033910	-1934.52891764
5	-1929.45976635	-1929.43587165	-1932.33168666	-1932.31687054	-1936.20393467	-1936.20711002	-1934.51789259	-1934.51430043
6	-1929.45722759	-1929.43587156	-1932.32664694	-1932.31687038	-1936.20222790	-1936.20710986	-1934.51597782	-1934.51430029
7	-1929.45722754	-1929.43329357	-1932.32664692	-1932.31578248	-1936.20222787	-1936.20068632	-1934.51597779	-1934.50883813
8	-1929.43348598	-1929.43288063	-1932.30666136	-1932.31445111	-1936.19026431	-1936.20441896	-1934.50106973	-1934.51153438
9	-1929.43348596	-1929.43288058	-1932.30666128	-1932.31444702	-1936.19026426	-1936.20441898	-1934.50106969	-1934.51153438
10	-1929.43060094	-1929.42113297	-1932.30284174	-1932.30388304	-1936.18950279	-1936.19381058	-1934.49977733	-1934.50064118
11		-1929.41249097		-1932.29305383		-1936.18322266		-1934.49053974
12		-1929.40590685		-1932.28797009		-1936.18416110		-1934.48959754
13		-1929.40590680		-1932.28796581		-1936.18416103		-1934.48959747
14		-1929.39821964		-1932.27920000		-1936.17616447		-1934.48167826
15		-1929.36585472		-1932.25727086		-1936.16767249		-1934.46721805

Table S58: Computed absolute energies with the SA-CASSCF, CASPT2, tPBE, and tPBE0 methods for the Ti(o-tol)$_4^{2-}$ complex when using the (8,8) active space.

	SA-CASSCF		CASPT2		tPBE		tPBE0	
No.	Triplet	Singlet	Triplet	Singlet	Triplet	Singlet	Triplet	Singlet
1	-1929.49649523	-1929.45419262	-1932.38163742	-1932.36471766	-1936.26639420	-1936.25914637	-1934.57391946	-1934.55790793
2	-1929.46755567	-1929.45417048	-1932.36643590	-1932.35500206	-1936.25345462	-1936.24782881	-1934.55697988	-1934.54941423
3	-1929.46433900	-1929.43271052	-1932.34963064	-1932.34337446	-1936.22640157	-1936.24127643	-1934.53588593	-1934.53913495
4	-1929.46433895	-1929.41903132	-1932.34963056	-1932.34515551	-1936.22640144	-1936.23964274	-1934.53588582	-1934.53448989
5	-1929.44922483	-1929.41750841	-1932.34315537	-1932.32792064	-1936.22229535	-1936.21211520	-1934.52902772	-1934.51346350
6	-1929.44320367	-1929.41750837	-1932.34229447	-1932.32792223	-1936.22223741	-1936.21211559	-1934.52747898	-1934.51346379
7	-1929.44320356	-1929.41553396	-1932.34229441	-1932.31843737	-1936.22223738	-1936.20491676	-1934.52747893	-1934.50757106
8	-1929.42306574	-1929.41553393	-1932.32207584	-1932.31843763	-1936.21652129	-1936.20491716	-1934.51815740	-1934.50757135
9	-1929.42306570	-1929.41264912	-1932.32207586	-1932.31761473	-1936.21652136	-1936.20158228	-1934.51815745	-1934.50434899
10	-1929.42193916	-1929.40483064	-1932.31278757	-1932.32122912	-1936.20495242	-1936.21230804	-1934.50919911	-1934.51043869
11		-1929.39826176		-1932.29805684		-1936.18903744		-1934.49134352
12		-1929.38605796		-1932.29851077		-1936.19413927		-1934.49211894
13		-1929.38605787		-1932.29850920		-1936.19413942		-1934.49211903
14		-1929.38187269		-1932.28274549		-1936.17974842		-1934.48027949
15		-1929.32285776		-1932.26424568		-1936.19292734		-1934.47540995

6.3.6 $Fe(o\text{-tol})_4^{2-}$ Complex

Table S59: Computed absolute energies with the SA-CASSCF, CASPT2, and tPBE methods for the $Fe(o\text{-tol})_4^{2-}$ complex when using the (6,5) active space.

	SA-CASSCF			CASPT2			tPBE		
No.	Quintet	Triplet	Singlet	Quintet	Triplet	Singlet	Quintet	Triplet	Singlet
1	-2348.03353430	-2347.95545424	-2347.90868802	-2351.12740928	-2351.06384979	-2351.03897428	-2355.05414078	-2355.01671931	-2354.99094483
2	-2348.03341646	-2347.95544011	-2347.90624556	-2351.12699850	-2351.06383449	-2351.03415896	-2355.05396763	-2355.01670044	-2354.99279581
3	-2348.01312218	-2347.95305698	-2347.90623454	-2351.10327757	-2351.06190568	-2351.03397334	-2355.03155777	-2355.01168657	-2354.99253440
4	-2348.01063671	-2347.93818860	-2347.90619589	-2351.10029320	-2351.05054689	-2351.03522716	-2355.02833566	-2354.98767838	-2354.98880524
5	-2348.01062167	-2347.93803178	-2347.90423677	-2351.10027635	-2351.05065474	-2351.03379565	-2355.02831963	-2354.98732758	-2354.98172323
6		-2347.93633446	-2347.89251390		-2351.04441864	-2351.02175944		-2354.98947596	-2354.96745806
7		-2347.93632521	-2347.89250447		-2351.04441086	-2351.02172642		-2354.98946263	-2354.96744384
8		-2347.93510870	-2347.89179225		-2351.05207838	-2351.02323944		-2354.98658930	-2354.96694404
9		-2347.93339467	-2347.88995943		-2351.04900482	-2351.01979726		-2354.98386752	-2354.96967194
10		-2347.93146151	-2347.88792303		-2351.03701743	-2351.02377100		-2354.98654152	-2354.96845674
11		-2347.93145208	-2347.88567151		-2351.03701208	-2351.01749978		-2354.98652604	-2354.96011940
12		-2347.92030149	-2347.88435856		-2351.03994208	-2351.00993965		-2354.97645554	-2354.95919163
13		-2347.91542282	-2347.88434600		-2351.02583084	-2351.00991208		-2354.96291142	-2354.95917706
14		-2347.91515828	-2347.87184153		-2351.03540627	-2350.99216089		-2354.98600724	-2354.94745672
15		-2347.91323292	-2347.86948623		-2351.02192392	-2350.99557636		-2354.95478657	-2354.94390156
16		-2347.91321841	-2347.86927681		-2351.02190462	-2350.98944121		-2354.95476333	-2354.94150264
17		-2347.91189357	-2347.86926434		-2351.02184118	-2350.98931230		-2354.95442461	-2354.94148740
18		-2347.90930155	-2347.86748009		-2351.01704861	-2350.99544090		-2354.95573800	-2354.93963262
19		-2347.90929232	-2347.85621308		-2351.01702928	-2350.99176986		-2354.95573093	-2354.94171515
20		-2347.90842660	-2347.85434604		-2351.02585047	-2350.98726327		-2354.97700010	-2354.94258848
21		-2347.90682568	-2347.85367811		-2351.02389067	-2350.99233852		-2354.97564844	-2354.94980862
22		-2347.90195592	-2347.84903277		-2351.01250042	-2350.96262933		-2354.96227091	-2354.92138666
23		-2347.90032911	-2347.84902945		-2351.01241985	-2350.96262409		-2354.95693632	-2354.92138341
24		-2347.90032516	-2347.84732966		-2351.01241031	-2350.95571189		-2354.95692353	-2354.92730570
25		-2347.88811829	-2347.82719098		-2350.98894377	-2350.96145315		-2354.93713841	-2354.94434629
26		-2347.88811579	-2347.82564935		-2350.98896631	-2350.93736987		-2354.93714724	-2354.90971476
27		-2347.88722173	-2347.82554500		-2350.99891853	-2350.95711648		-2354.95709328	-2354.93461538
28		-2347.88719686	-2347.82554294		-2350.99746929	-2350.95709381		-2354.95175022	-2354.93456779
29		-2347.88719620	-2347.81329155		-2350.99761473	-2350.95570495		-2354.95188163	-2354.94473283
30		-2347.88716134	-2347.81216389		-2350.98477155	-2350.95179448		-2354.93820214	-2354.93372825
31		-2347.87709185	-2347.81215644		-2351.00476664	-2350.95178954		-2354.97226954	-2354.93373742
32		-2347.87494419	-2347.80890757		-2351.00122977	-2350.94869184		-2354.96834511	-2354.94031207
33		-2347.86783003	-2347.80832631		-2350.99554506	-2350.94889763		-2354.96391724	-2354.93338963
34		-2347.86724768	-2347.80831623		-2350.99373334	-2350.94888715		-2354.96375011	-2354.93338009
35		-2347.86724664	-2347.80812841		-2350.99372917	-2350.94972455		-2354.96375216	-2354.94253834
36			-2347.75506879			-2350.91357829			-2354.94406755
37			-2347.75047206			-2350.90897638			-2354.94656174
38			-2347.74977764			-2350.90721251			-2354.94506785
39			-2347.74977417			-2350.90720634			-2354.94505725
40			-2347.74444814			-2350.89877323			-2354.93811749
41			-2347.74291449			-2350.89500947			-2354.93654286
42			-2347.72326354			-2350.87012036			-2354.91801753
43			-2347.72326132			-2350.87012394			-2354.91801762
44			-2347.72274007			-2350.86863378			-2354.91651254
45			-2347.65157530			-2350.82702597			-2354.92619789

Table S60: Computed absolute energies with the tPBE0 method for the Fe(o-tol)$_4^{2-}$ complex when using the (6,5) active space.

	tPBE0		
No.	Quintet	Triplet	Singlet
1	-2353.29898916	-2353.25140304	-2353.22038063
2	-2353.29882984	-2353.25138536	-2353.22115825
3	-2353.27694887	-2353.24702917	-2353.22095944
4	-2353.27391092	-2353.22530594	-2353.21815290
5	-2353.27389514	-2353.22500363	-2353.21235162
6		-2353.22619059	-2353.19872202
7		-2353.22617828	-2353.19870900
8		-2353.22371915	-2353.19815609
9		-2353.22124931	-2353.19974381
10		-2353.22277152	-2353.19832331
11		-2353.22275755	-2353.19150743
12		-2353.21241703	-2353.19048336
13		-2353.20103927	-2353.19046930
14		-2353.21829500	-2353.17855292
15		-2353.19439816	-2353.17529773
16		-2353.19437710	-2353.17344618
17		-2353.19379185	-2353.17343164
18		-2353.19412889	-2353.17159449
19		-2353.19412128	-2353.17033963
20		-2353.20985673	-2353.17052787
21		-2353.20844275	-2353.17577599
22		-2353.19719216	-2353.15329819
23		-2353.19278452	-2353.15329492
24		-2353.19277394	-2353.15731169
25		-2353.17488338	-2353.16505746
26		-2353.17488938	-2353.13869841
27		-2353.18962539	-2353.15734779
28		-2353.18561188	-2353.15731158
29		-2353.18571027	-2353.16187251
30		-2353.17544194	-2353.15333716
31		-2353.19847512	-2353.15334218
32		-2353.19499488	-2353.15746095
33		-2353.18989544	-2353.15212380
34		-2353.18962450	-2353.15211413
35		-2353.18962578	-2353.15893586
36			-2353.14681786
37			-2353.14753932
38			-2353.14624530
39			-2353.14623648
40			-2353.13970015
41			-2353.13813577
42			-2353.11932903
43			-2353.11932855
44			-2353.11806942
45			-2353.10754224

Table S61: Computed absolute energies with the SA-CASSCF, CASPT2, tPBE methods for the Fe(o-tol)$_4^{2-}$ complex when using the (6,10) active space.

	SA-CASSCF			CASPT2			tPBE		
No.	Quintet	Triplet	Singlet	Quintet	Triplet	Singlet	Quintet	Triplet	Singlet
1	-2348.10942766	-2348.03570712	-2347.99126226	-2351.10435641	-2351.04081246	-2351.01072056	-2355.02748922	-2354.99022036	-2354.96064998
2	-2348.10825818	-2348.03569149	-2347.98860065	-2351.10339409	-2351.04079335	-2351.00575541	-2355.02655347	-2354.99020049	-2354.96310267
3	-2348.08806310	-2348.03318459	-2347.98858868	-2351.07990060	-2351.03853959	-2351.00567862	-2355.00476724	-2354.98516656	-2354.96280474
4	-2348.08548914	-2348.01697981	-2347.98855120	-2351.07693095	-2351.02337990	-2351.00681547	-2355.00132518	-2354.95829914	-2354.95769757
5	-2348.08547534	-2348.01684165	-2347.98654112	-2351.07691458	-2351.02364021	-2351.00437299	-2355.00130966	-2354.95835714	-2354.95249795
6		-2348.01628045	-2347.97380100		-2351.02047457	-2350.99118000		-2354.96336168	-2354.93459065
7		-2348.01627190	-2347.97379179		-2351.02045910	-2350.99116388		-2354.96334842	-2354.93458007
8		-2348.01453079	-2347.97316401		-2351.02492443	-2350.99200959		-2354.95726933	-2354.93342606
9		-2348.01310690	-2347.97092348		-2351.02215041	-2350.98772167		-2354.95625248	-2354.93369219
10		-2348.01059569	-2347.97016206		-2351.01351008	-2350.99145915		-2354.96040298	-2354.93538292
11		-2348.01058557	-2347.96676151		-2351.01355787	-2350.98532172		-2354.96038656	-2354.92493200
12		-2348.00028557	-2347.96533139		-2351.01121619	-2350.97988979		-2354.94721429	-2354.92676625
13		-2347.99564467	-2347.96531852		-2351.00828454	-2350.97986723		-2354.95940489	-2354.92675389
14		-2347.99405815	-2347.95192702		-2350.99755224	-2350.96169006		-2354.93370035	-2354.91527202
15		-2347.99170618	-2347.94999771		-2350.99517400	-2350.96576171		-2354.92842045	-2354.91199844
16		-2347.99169123	-2347.94908157		-2350.99515083	-2350.95854562		-2354.92839717	-2354.90826806
17		-2347.99087232	-2347.94906933		-2350.99526702	-2350.95852194		-2354.92710469	-2354.90825084
18		-2347.98853439	-2347.94780573		-2350.99947974	-2350.96644736		-2354.94868347	-2354.90635805
19		-2347.98804141	-2347.93849830		-2350.99081500	-2350.96059984		-2354.92744191	-2354.90347149
20		-2347.98803156	-2347.93648256		-2350.99080021	-2350.96096632		-2354.92743351	-2354.91084048
21		-2347.98674134	-2347.93586093		-2350.99797539	-2350.95408708		-2354.94816881	-2354.90202339
22		-2347.98168949	-2347.92852518		-2350.98682218	-2350.93502568		-2354.93532964	-2354.89325731
23		-2347.97957972	-2347.92852200		-2350.98562177	-2350.93502396		-2354.92987929	-2354.89325502
24		-2347.97957504	-2347.92646258		-2350.98561059	-2350.93091488		-2354.92986921	-2354.89646974
25		-2347.96663674	-2347.90973250		-2350.97078767	-2350.93136717		-2354.92382052	-2354.90764310
26		-2347.96662854	-2347.90776491		-2350.97083241	-2350.92831851		-2354.92387873	-2354.90036735
27		-2347.96652806	-2347.90776109		-2350.97266380	-2350.92830222		-2354.92842932	-2354.90032749
28		-2347.96612983	-2347.90575206		-2350.96487178	-2350.91470947		-2354.91434383	-2354.88317536
29		-2347.96612468	-2347.89749395		-2350.96487570	-2350.92553600		-2354.91440771	-2354.90622450
30		-2347.96510661	-2347.89635189		-2350.96196441	-2350.92317934		-2354.91215392	-2354.89572630
31		-2347.96038076	-2347.89634452		-2350.97943745	-2350.92317338		-2354.94278205	-2354.89573470
32		-2347.95791564	-2347.89277526		-2350.97610005	-2350.92017353		-2354.93872448	-2354.90531907
33		-2347.95109467	-2347.89255856		-2350.97069493	-2350.91910928		-2354.93548939	-2354.89626272
34		-2347.95044180	-2347.89254844		-2350.96927595	-2350.91909620		-2354.93430813	-2354.89624131
35		-2347.95044076	-2347.89229839		-2350.96927713	-2350.91965291		-2354.93430499	-2354.90577370
36			-2347.84682344			-2350.88284593			-2354.89168188
37			-2347.84260666			-2350.88179106			-2354.90098807
38			-2347.84181619			-2350.87984994			-2354.89828341
39			-2347.84181207			-2350.87984233			-2354.89826849
40			-2347.83621904			-2350.87114798			-2354.89360758
41			-2347.83451052			-2350.86952142			-2354.89278950
42			-2347.81364258			-2350.84285764			-2354.87420688
43			-2347.81363978			-2350.84286315			-2354.87420723
44			-2347.81298960			-2350.84129239			-2354.87272447
45			-2347.74814750			-2350.80345469			-2354.87741740

Table S62: Computed absolute energies with the tPBE0 methods for the $Fe(o\text{-tol})_4^{2-}$ complex when using the (6,10) active space.

	tPBE0		
No.	Quintet	Triplet	Singlet
1	-2353.29797383	-2353.25159205	-2353.21830305
2	-2353.29697965	-2353.25157324	-2353.21947717
3	-2353.27559121	-2353.24717107	-2353.21925073
4	-2353.27236617	-2353.22296931	-2353.21541098
5	-2353.27235108	-2353.22297827	-2353.21100874
6		-2353.22659137	-2353.19439324
7		-2353.22657929	-2353.19438300
8		-2353.22158470	-2353.19336055
9		-2353.22046609	-2353.19300001
10		-2353.22295116	-2353.19407771
11		-2353.22293631	-2353.18538938
12		-2353.21048211	-2353.18640754
13		-2353.21846484	-2353.18639505
14		-2353.19878980	-2353.17443577
15		-2353.19424188	-2353.17149826
16		-2353.19422069	-2353.16847144
17		-2353.19304660	-2353.16845546
18		-2353.20864620	-2353.16671997
19		-2353.19259179	-2353.16222819
20		-2353.19258302	-2353.16725100
21		-2353.20781194	-2353.16048278
22		-2353.19691960	-2353.15207428
23		-2353.19230440	-2353.15207177
24		-2353.19229567	-2353.15396795
25		-2353.18452458	-2353.15816545
26		-2353.18456618	-2353.15221674
27		-2353.18795401	-2353.15218589
28		-2353.17729033	-2353.13881954
29		-2353.17733695	-2353.15404186
30		-2353.17539209	-2353.14588270
31		-2353.19718173	-2353.14588716
32		-2353.19352227	-2353.15218312
33		-2353.18939071	-2353.14533668
34		-2353.18834155	-2353.14531809
35		-2353.18833893	-2353.15240487
36			-2353.13046727
37			-2353.13639272
38			-2353.13416661
39			-2353.13415439
40			-2353.12926045
41			-2353.12821976
42			-2353.10906581
43			-2353.10906537
44			-2353.10779075
45			-2353.09509993

Table S63: Computed absolute energies with the SA-CASSCF, CASPT2, and tPBE methods for the Fe(o-tol)$_4^{2-}$ complex when using the (12,8) active space.

	SA-CASSCF			CASPT2			tPBE		
No.	Quintet	Triplet	Singlet	Quintet	Triplet	Singlet	Quintet	Triplet	Singlet
1	-2348.02442758	-2347.94876515	-2347.90093870	-2351.12503860	-2351.05137115	-2351.01252326	-2355.04772168	-2355.00049233	-2354.96039135
2	-2348.02426197	-2347.94858554	-2347.89933763	-2351.12456882	-2351.05139729	-2351.00747570	-2355.04711770	-2355.00038841	-2354.96128486
3	-2348.00296218	-2347.94636401	-2347.89932728	-2351.10164171	-2351.04979523	-2351.00747311	-2355.02444172	-2354.99589671	-2354.96127222
4	-2348.00058236	-2347.92904796	-2347.89856045	-2351.09894091	-2351.03741932	-2351.01004177	-2355.02162474	-2354.96970944	-2354.95916998
5	-2348.00056662	-2347.92902634	-2347.89734986	-2351.09892238	-2351.03769871	-2351.00718857	-2355.02160823	-2354.96976223	-2354.95306636
6		-2347.92761688			-2351.03451365			-2354.97477209	
7		-2347.92747146			-2351.03457091			-2354.97464972	
8		-2347.92643472			-2351.04042378			-2354.97164982	
9		-2347.92413877			-2351.03735526			-2354.96855532	
10		-2347.92255983			-2351.02752704			-2354.97111159	
11		-2347.92242771			-2351.02756182			-2354.97105278	
12		-2347.91092403			-2351.02800149			-2354.95957560	
13		-2347.90678182			-2351.02411400			-2354.97317674	
14		-2347.90443081			-2351.01344634			-2354.94450842	
15		-2347.90240983			-2351.01180324			-2354.94002169	

Table S64: Computed absolute energies with the tPBE0 method for the Fe(o-tol)$_4^{2-}$ complex when using the (12,8) active space.

	tPBE0		
No.	Quintet	Triplet	Singlet
1	-2353.29189816	-2353.23756054	-2353.19552819
2	-2353.29140377	-2353.23743769	-2353.19579805
3	-2353.26907184	-2353.23351354	-2353.19578599
4	-2353.26636415	-2353.20954407	-2353.19401760
5	-2353.26634783	-2353.20957826	-2353.18913724
6		-2353.21298329	
7		-2353.21285516	
8		-2353.21034605	
9		-2353.20745118	
10		-2353.20897365	
11		-2353.20889651	
12		-2353.19741271	
13		-2353.20657801	
14		-2353.18448902	
15		-2353.18061873	

6.3.7 Co(o-tol)$_4^{2-}$Complex

Table S65: Computed absolute energies with the SA-CASSCF, CASPT2, tPBE, and tPBE0 methods for the Co(o-tol)$_4^{2-}$ complex when using the (7,5) active space.

	SA-CASSCF		CASPT2		tPBE		tPBE0	
No.	Quartet	Doublet	Quartet	Doublet	Quartet	Doublet	Quartet	Doublet
1	-2468.56262326	-2468.47402236	-2471.71360371	-2471.64566201	-2475.69079818	-2475.63457075	-2473.90875445	-2473.84443365
2	-2468.54051859	-2468.47391427	-2471.68537461	-2471.64554629	-2475.66471168	-2475.63502711	-2473.88366341	-2473.84474890
3	-2468.53831308	-2468.47391324	-2471.68196849	-2471.64554537	-2475.66134710	-2475.63502464	-2473.88058860	-2473.84474679
4	-2468.53831084	-2468.47255084	-2471.68188675	-2471.64438306	-2475.66134472	-2475.63670986	-2473.88058625	-2473.84567011
5	-2468.52213244	-2468.47210560	-2471.66530975	-2471.64338936	-2475.63798240	-2475.63691679	-2473.85901991	-2473.84571399
6	-2468.52213081	-2468.46551725	-2471.66530831	-2471.63297171	-2475.63798158	-2475.61281212	-2473.85901889	-2473.82598840
7	-2468.52126247	-2468.45584247	-2471.66759212	-2471.62406941	-2475.63387378	-2475.60852255	-2473.85572095	-2473.82035253
8	-2468.45789988	-2468.45466767	-2471.62509357	-2471.62256626	-2475.63375250	-2475.60656015	-2473.83978935	-2473.81858703
9	-2468.45690696	-2468.45466612	-2471.62138339	-2471.62256461	-2475.62425467	-2475.60655734	-2473.83241774	-2473.81858454
10	-2468.45690516	-2468.44325697	-2471.62138346	-2471.61803320	-2475.62425145	-2475.61085572	-2473.83241488	-2473.81895603
11		-2468.44235150		-2471.61067311		-2475.60932289		-2473.81758004
12		-2468.44233008		-2471.61507640		-2475.60651116		-2473.81546589
13		-2468.44232844		-2471.61505966		-2475.60647800		-2473.81544061
14		-2468.44013677		-2471.60840495		-2475.59958172		-2473.80972048
15		-2468.44013486		-2471.60840141		-2475.59958274		-2473.80972077
16		-2468.43698920		-2471.60817984		-2475.60372684		-2473.81204243
17		-2468.43393888		-2471.60364523		-2475.59961667		-2473.80819722
18		-2468.42425883		-2471.59301711		-2475.59093651		-2473.79926709
19		-2468.42384258		-2471.58968376		-2475.59022024		-2473.79862583
20		-2468.42384133		-2471.58968317		-2475.59021861		-2473.79862429
21		-2468.41907902		-2471.57951989		-2475.57723816		-2473.78769838
22		-2468.41907715		-2471.57951527		-2475.57723451		-2473.78769517
23		-2468.41684565		-2471.57425556		-2475.57555052		-2473.78587430
24		-2468.41047107		-2471.56968634		-2475.57156605		-2473.78129231
25		-2468.41046906		-2471.56968071		-2475.57156395		-2473.78129023
26		-2468.41001993		-2471.56661388		-2475.56949177		-2473.77962381
27		-2468.39725966		-2471.54285412		-2475.55794871		-2473.76777645
28		-2468.39657587		-2471.54310758		-2475.55889804		-2473.76831750
29		-2468.36584018		-2471.55584611		-2475.60621304		-2473.79611983
30		-2468.35892149		-2471.54898571		-2475.60073910		-2473.79028470

Table S66: Computed absolute energies with the SA-CASSCF, CASPT2, tPBE, and tPBE0 methods for the Co(o-tol)$_4^{2-}$ complex when using the (7,10) active space.

	SA-CASSCF		CASPT2		tPBE		tPBE0	
No.	Quartet	Doublet	Quartet	Doublet	Quartet	Doublet	Quartet	Doublet
1	-2468.66666100	-2468.58054343	-2471.67482143	-2471.60307962	-2475.65166696	-2475.59269212	-2473.90541547	-2473.83965495
2	-2468.64408672	-2468.58045498	-2471.64798802	-2471.60331247	-2475.62626140	-2475.59263190	-2473.88071773	-2473.83958767
3	-2468.64143606	-2468.58045381	-2471.64503546	-2471.60331097	-2475.62275686	-2475.59262954	-2473.87742666	-2473.83958561
4	-2468.64143417	-2468.57896513	-2471.64503298	-2471.60065227	-2475.62275442	-2475.59369340	-2473.87742436	-2473.84001133
5	-2468.62519345	-2468.57854685	-2471.62739605	-2471.60000115	-2475.60104922	-2475.59337120	-2473.85708528	-2473.83966511
6	-2468.62519181	-2468.57013092	-2471.62739408	-2471.58892551	-2475.60104840	-2475.56768761	-2473.85708425	-2473.81829844
7	-2468.62432154	-2468.56119514	-2471.62843988	-2471.58118456	-2475.59674594	-2475.56484023	-2473.85363984	-2473.81392896
8	-2468.56901058	-2468.55990717	-2471.58767475	-2471.58011779	-2475.59507098	-2475.56371263	-2473.83855588	-2473.81276127
9	-2468.56830710	-2468.55990542	-2471.58612922	-2471.58011519	-2475.58587080	-2475.56370979	-2473.83147988	-2473.81275870
10	-2468.56830528	-2468.55122311	-2471.58612759	-2471.57354731	-2475.58586781	-2475.56392571	-2473.83147718	-2473.81075006
11		-2468.54994625		-2471.57145681		-2475.56139497		-2473.80853279
12		-2468.54994473		-2471.57145435		-2475.56139240		-2473.80853048
13		-2468.54929073		-2471.56821027		-2475.56380789		-2473.81017860
14		-2468.54686385		-2471.56569486		-2475.55796921		-2473.80519287
15		-2468.54686173		-2471.56569155		-2475.55796620		-2473.80519008
16		-2468.54455575		-2471.56634975		-2475.55965920		-2473.80588334
17		-2468.54116289		-2471.56169825		-2475.55514539		-2473.80164977
18		-2468.53066846		-2471.55086273		-2475.54546703		-2473.79176739
19		-2468.53017042		-2471.54763527		-2475.54496519		-2473.79126650
20		-2468.53016909		-2471.54763512		-2475.54496391		-2473.79126521
21		-2468.52526288		-2471.53849937		-2475.53293355		-2473.78101588
22		-2468.52526105		-2471.53849507		-2475.53292996		-2473.78101273
23		-2468.52275988		-2471.53392266		-2475.53057888		-2473.77862413
24		-2468.51663055		-2471.53045613		-2475.52890815		-2473.77583875
25		-2468.51662855		-2471.53045291		-2475.52890665		-2473.77583713
26		-2468.51582147		-2471.52843189		-2475.52744282		-2473.77453748
27		-2468.50245206		-2471.50838454		-2475.51774253		-2473.76391991
28		-2468.50194154		-2471.50812527		-2475.51867903		-2473.76449466
29		-2468.47884023		-2471.51500790		-2475.55840144		-2473.78851114
30		-2468.47234521		-2471.50773472		-2475.55301325		-2473.78284624

Table S67: Computed absolute energies with the SA-CASSCF, CASPT2, tPBE, and tPBE0 methods for the Co(o-tol)$_4^{2-}$ complex when using the (13,8) active space.

	SA-CASSCF		CASPT2		tPBE		tPBE0	
No.	Quartet	Doublet	Quartet	Doublet	Quartet	Doublet	Quartet	Doublet
1	-2468.55751725	-2468.46904796	-2471.70967325	-2471.63697336	-2475.68566875	-2475.62592414	-2473.90363088	-2473.83670510
2	-2468.53425377	-2468.46892791	-2471.68247826	-2471.63671599	-2475.65890224	-2475.62674140	-2473.87774012	-2473.83728803
3	-2468.53210897	-2468.46892675	-2471.67944689	-2471.63671478	-2475.65551879	-2475.62673620	-2473.87466634	-2473.83728384
4	-2468.53210650	-2468.46760597	-2471.67944659	-2471.63376746	-2475.65551651	-2475.62568806	-2473.87466401	-2473.83616754
5	-2468.51502581	-2468.46735842	-2471.66369790	-2471.63292470	-2475.63147229	-2475.62601854	-2473.85236067	-2473.83635351
6	-2468.51502421	-2468.45969136	-2471.66369850	-2471.62589300	-2475.63147222	-2475.60460290	-2473.85236022	-2473.81837502
7	-2468.51426418	-2468.44987086	-2471.66552148	-2471.61488436	-2475.62775157	-2475.59709347	-2473.84937972	-2473.81028782
8	-2468.45193387	-2468.44880409	-2471.62592674	-2471.61313638	-2475.63168217	-2475.59576490	-2473.83674510	-2473.80902470
9	-2468.45099604	-2468.44880247	-2471.62278751	-2471.61313516	-2475.62234631	-2475.59576329	-2473.82950874	-2473.80902309
10	-2468.45099393	-2468.43804573	-2471.62278457	-2471.61010739	-2475.62234225	-2475.60139490	-2473.82950517	-2473.81055761
11		-2468.43711647		-2471.60800710		-2475.59876979		-2473.80835646
12		-2468.43711505		-2471.60800472		-2475.59876843		-2473.80835509
13		-2468.43638473		-2471.60314528		-2475.60040197		-2473.80939766
14		-2468.43448395		-2471.60119257		-2475.59429889		-2473.80434516
15		-2468.43448175		-2471.60118954		-2475.59429780		-2473.80434379
16		-2468.43106273		-2471.60357261		-2475.59743668		-2473.80584319
17		-2468.42846846		-2471.59981639		-2475.59419373		-2473.80276241
18		-2468.41742781		-2471.58483145		-2475.58056321		-2473.78977936
19		-2468.41713607		-2471.58264495		-2475.58077024		-2473.78986170
20		-2468.41713495		-2471.58264581		-2475.58076837		-2473.78986002
21		-2468.41196871		-2471.57270279		-2475.56710242		-2473.77831899
22		-2468.41196677		-2471.57269894		-2475.56709938		-2473.77831623
23		-2468.41014861		-2471.56904745		-2475.56729911		-2473.77801149
24		-2468.40347844		-2471.56058924		-2475.55941569		-2473.77043138
25		-2468.40347626		-2471.56058591		-2475.55941359		-2473.77042926
26		-2468.40278385		-2471.55793327		-2475.55714718		-2473.76855635
27		-2468.38922413		-2471.53597819		-2475.54677209		-2473.75738510
28		-2468.38887906		-2471.53607844		-2475.54796947		-2473.75819687
29		-2468.36060890		-2471.55638561		-2475.60440307		-2473.79345453
30		-2468.35288251		-2471.54680562		-2475.59583673		-2473.78509818

6.3.8 Ni(o-tol)$_4^{2-}$ Complex

Table S68: Computed absolute energies with the SA-CASSCF, CASPT2, tPBE, and tPBE0 methods for the Ni(o-tol)$_4^{2-}$ complex when using the (8,5) active space.

	SA-CASSCF		CASPT2		tPBE		tPBE0	
No.	Triplet	Singlet	Triplet	Singlet	Triplet	Singlet	Triplet	Singlet
1	-2595.76033140	-2595.68877214	-2599.02367277	-2598.98059564	-2602.97978466	-2602.95733332	-2601.17492135	-2601.14019303
2	-2595.74316715	-2595.68263265	-2599.00160181	-2598.97629425	-2602.95234049	-2602.95776257	-2601.15004716	-2601.13898009
3	-2595.74134021	-2595.66965270	-2598.99866603	-2598.95455724	-2602.95040052	-2602.93256768	-2601.14813544	-2601.11683894
4	-2595.73826874	-2595.66814464	-2598.99096893	-2598.95077512	-2602.95159208	-2602.92744486	-2601.14826125	-2601.11261981
5	-2595.72745154	-2595.66509729	-2598.98112618	-2598.94731638	-2602.92983432	-2602.92279259	-2601.12923863	-2601.10836877
6	-2595.72197656	-2595.63916711	-2598.97056683	-2598.93358443	-2602.92508859	-2602.93584797	-2601.12431058	-2601.11167776
7	-2595.71098145	-2595.63793053	-2598.95337036	-2598.91921761	-2602.91681911	-2602.89762385	-2601.11535970	-2601.08270052
8	-2595.65752537	-2595.63565910	-2598.93337581	-2598.92116352	-2602.91888009	-2602.92232451	-2601.10354141	-2601.10065816
9	-2595.65067430	-2595.62654440	-2598.92305910	-2598.90782497	-2602.90665654	-2602.90419862	-2601.09266098	-2601.08478507
10	-2595.64482478	-2595.62242957	-2598.91616210	-2598.89787957	-2602.90054617	-2602.89327944	-2601.08661582	-2601.07556697
11		-2595.62005718		-2598.89403905		-2602.89945692		-2601.07960699
12		-2595.61347417		-2598.88293123		-2602.87159096		-2601.05706176
13		-2595.61230244		-2598.87565359		-2602.88181098		-2601.06443385
14		-2595.60972021		-2598.87477715		-2602.87352644		-2601.05757488
15		-2595.46862630		-2598.78038437		-2602.89691993		-2601.03984652

Table S69: Computed absolute energies with the SA-CASSCF, CASPT2, tPBE, and tPBE0 methods for the Mo(o-tol)$_4$ complex when using the (8,10) active space.

	SA-CASSCF		CASPT2		tPBE		tPBE0	
No.	Triplet	Singlet	Triplet	Singlet	Triplet	Singlet	Triplet	Singlet
1	-2595.89600988	-2595.82652930	-2598.97049546	-2598.91884518	-2602.92631720	-2602.89538458	-2601.16874037	-2601.12817076
2	-2595.87808818	-2595.82003747	-2598.94883375	-2598.91218972	-2602.89995966	-2602.89491720	-2601.14449179	-2601.12619727
3	-2595.87606487	-2595.80586844	-2598.94621507	-2598.89289727	-2602.89800858	-2602.87091669	-2601.14252265	-2601.10465463
4	-2595.87287003	-2595.80357821	-2598.94095768	-2598.88948982	-2602.90000451	-2602.86590428	-2601.14322089	-2601.10032276
5	-2595.86181906	-2595.80068879	-2598.92947920	-2598.88686996	-2602.87977230	-2602.85924907	-2601.12528399	-2601.09460900
6	-2595.85578315	-2595.77767406	-2598.92094163	-2598.86990430	-2602.87498223	-2602.87076644	-2601.12018246	-2601.09749335
7	-2595.84455842	-2595.77542530	-2598.90681786	-2598.86193106	-2602.86775656	-2602.83629191	-2601.11195703	-2601.07107526
8	-2595.79724932	-2595.77304318	-2598.88042606	-2598.85999933	-2602.86597769	-2602.85699639	-2601.09879560	-2601.08600809
9	-2595.79027629	-2595.76380882	-2598.87225017	-2598.84832467	-2602.85420564	-2602.83522017	-2601.08822330	-2601.06736733
10	-2595.78465972	-2595.75919626	-2598.86623155	-2598.84071251	-2602.84865862	-2602.83273622	-2601.08265890	-2601.06435123
11		-2595.75697219		-2598.83885518		-2602.83951938		-2601.06888258
12		-2595.75031989		-2598.82958110		-2602.81234214		-2601.04683658
13		-2595.74828482		-2598.82381850		-2602.82305169		-2601.05435997
14		-2595.74617420		-2598.82301541		-2602.81668776		-2601.04905937
15		-2595.61389358		-2598.71824169		-2602.81086450		-2601.01162177

Table S70: Computed absolute energies with the SA-CASSCF, CASPT2, tPBE, and tPBE0 methods for the Ni(o-tol)$_4^{2-}$ complex when using the (14,8) active space.

	SA-CASSCF		CASPT2		tPBE		tPBE0	
No.	Triplet	Singlet	Triplet	Singlet	Triplet	Singlet	Triplet	Singlet
1	-2595.75780754	-2595.68861858	-2599.01920810	-2598.96896509	-2602.97578376	-2602.94835579	-2601.17128971	-2601.13342149
2	-2595.73944556	-2595.68289068	-2598.99778005	-2598.96165820	-2602.94866124	-2602.94707790	-2601.14635732	-2601.13103110
3	-2595.73766442	-2595.66897606	-2598.99491082	-2598.94324578	-2602.94691353	-2602.92322494	-2601.14460125	-2601.10966272
4	-2595.73436303	-2595.66737109	-2598.98738698	-2598.94019848	-2602.94709689	-2602.91875718	-2601.14391343	-2601.10591066
5	-2595.72318636	-2595.66423019	-2598.97853411	-2598.93907274	-2602.92489803	-2602.91552543	-2601.12447011	-2601.10270162
6	-2595.71740434	-2595.63850817	-2598.96819010	-2598.92492907	-2602.91989709	-2602.92919940	-2601.11927390	-2601.10652659
7	-2595.70610832	-2595.63667373	-2598.95102674	-2598.91394955	-2602.91217855	-2602.89180493	-2601.11066099	-2601.07802213
8	-2595.65409733	-2595.63449181	-2598.93554905	-2598.91360200	-2602.91904054	-2602.91554587	-2601.10280474	-2601.09528236
9	-2595.64708899	-2595.62531173	-2598.92449798	-2598.90088971	-2602.90598667	-2602.89740306	-2601.09126225	-2601.07938023
10	-2595.64128670	-2595.62109493	-2598.91804869	-2598.89244066	-2602.90024710	-2602.88758565	-2601.08550700	-2601.07096297
11		-2595.61871086		-2598.88805118		-2602.89337370		-2601.07470799
12		-2595.61205859		-2598.87785730		-2602.86641422		-2601.05282531
13		-2595.61100275		-2598.87022817		-2602.87554146		-2601.05940678
14		-2595.60838224		-2598.86925015		-2602.86790315		-2601.05302292
15		-2595.47219768		-2598.80075314		-2602.90004484		-2601.04308305

7 Geometrical Distortions Following Lower Energetic Vibrational Modes

Using the geometry optimized structures with DFT, we selected two vibrational modes of Ti(o-tol)$_4^{2-}$, V(o-tol)$_4^{-}$, and Cr(o-tol)$_4$ complexes related to the symmetric stretching of the metal-aryl bonds, and the aryl-metal-aryl scissoring modes (See Figure S23). Following the normal modes, we selected eight structures for which we computed the triplet ground state T_0, the first singlet and triplet excited states S_1 and T_1, and associated energy gaps: $\Delta E_{T_0-S_1}$, $\Delta E_{T_0-T_1}$, and $\Delta E_{S_1-T_1}$. The energy differences are reported in Tables S71-S76. Additionally, the relative energies for the lowest-lying triplet and singlet excited states and the triplet ground state are shown in Figures S24-S29.

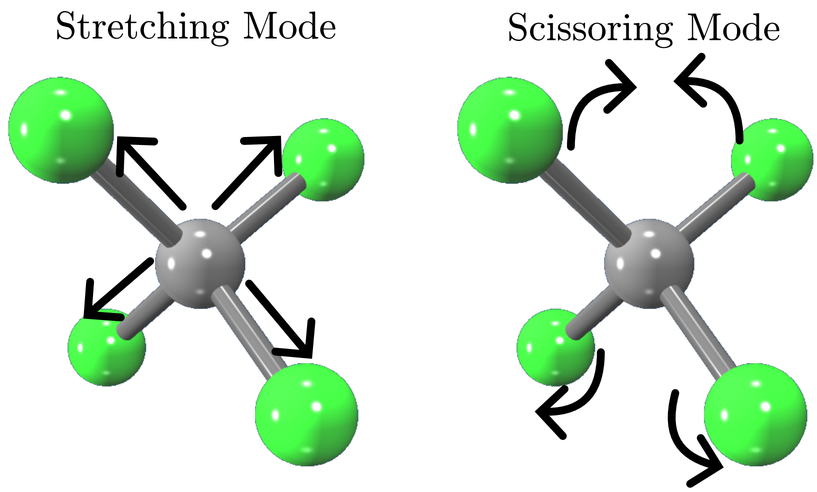

Figure S23: Schemes for the stretching and scissoring modes between the metal center and the o−tolyl ligands considered for the geometrical distortions of Cr(o-tol)$_4$, V(o-tol)$_4^{-}$, Ti(o-tol)$_4^{2-}$ complexes.

Table S71: Energy differences in eV between ground state T_0 and first excited state S_1, between the two triplet excited states T_0-T_1, and between the singlet and triplet excited states S_1-T_1 of Cr(o-tol)$_4$ complex for the Cr–C symmetric stretching. The equilibrium bond distance is $d = 1.98$ Å.

	$\Delta E_{T_0-S_1}$		$\Delta E_{T_0-T_1}$		$\Delta E_{S_1-T_1}$	
Δd (Å)	CASPT2	tPBE0	CASPT2	tPBE0	CASPT2	tPBE0
0.077	1.52	1.29	2.00	2.06	0.48	0.76
0.058	1.50	1.28	2.03	2.08	0.52	0.80
0.039	1.49	1.27	2.06	2.11	0.57	0.85
0.019	1.47	1.25	2.09	2.14	0.62	0.89
0.000	1.46	1.24	2.13	2.17	0.67	0.93
-0.019	1.44	1.22	2.16	2.20	0.72	0.97
-0.039	1.43	1.21	2.19	2.23	0.77	1.02
-0.058	1.41	1.20	2.23	2.26	0.82	1.06
-0.077	1.39	1.18	2.26	2.29	0.87	1.11

Table S72: Energy differences in eV between ground state T_0 and first excited state S_1, between the two triplet excited states T_0-T_1, and between the singlet and triplet excited states S_1-T_1 of Cr(o-tol)$_4$ complex for the C–Cr–C scissoring mode. The equilibrium bond angle is $\theta = 104.9°$.

	$\Delta E_{T_0-S_1}$		$\Delta E_{T_0-T_1}$		$\Delta E_{S_1-T_1}$	
$\Delta\theta$ (°)	CASPT2	tPBE0	CASPT2	tPBE0	CASPT2	tPBE0
-6.569	1.44	1.24	2.06	2.19	0.62	0.95
-4.922	1.45	1.24	2.13	2.18	0.69	0.94
-3.277	1.45	1.24	2.13	2.17	0.68	0.94
-1.636	1.45	1.24	2.13	2.17	0.67	0.93
0.000	1.46	1.24	2.13	2.17	0.67	0.93
1.634	1.45	1.24	2.13	2.17	0.67	0.93
3.261	1.45	1.24	2.13	2.17	0.68	0.94
4.882	1.45	1.24	2.13	2.18	0.69	0.94
6.497	1.44	1.24	2.06	2.19	0.62	0.95

Table S73: Energy differences in eV between ground state T_0 and first excited state S_1, between the two triplet excited states T_0-T_1, and between the singlet and triplet excited states S_1-T_1 of $V(o\text{-tol})_4^-$ complex for the V–C symmetric stretching. The equilibrium bond distance is $d = 2.07$ Å.

	$\Delta E_{T_0-S_1}$		$\Delta E_{T_0-T_1}$		$\Delta E_{S_1-T_1}$	
Δd (Å)	CASPT2	tPBE0	CASPT2	tPBE0	CASPT2	tPBE0
-0.098	1.09	0.78	1.49	1.45	0.40	0.67
-0.074	1.11	0.80	1.44	1.40	0.32	0.60
-0.049	1.14	0.82	1.39	1.35	0.25	0.53
-0.025	1.16	0.84	1.34	1.30	0.18	0.46
0.000	1.17	0.85	1.29	1.25	0.11	0.40
0.025	1.19	0.87	1.24	1.21	0.05	0.34
0.049	1.21	0.88	1.20	1.17	-0.01	0.28
0.074	1.22	0.90	1.16	1.13	-0.07	0.23
0.099	1.24	0.91	1.12	1.09	-0.12	0.17

Table S74: Energy differences in eV between ground state T_0 and first excited state S_1, between the two triplet excited states T_0-T_1, and between the singlet and triplet excited states S_1-T_1 of $V(o\text{-tol})_4^-$ complex for the C–V–C scissoring mode. The equilibrium bond angle is $\theta = 105.2°$.

	$\Delta E_{T_0-S_1}$		$\Delta E_{T_0-T_1}$		$\Delta E_{S_1-T_1}$	
$\Delta\theta$ (°)	CASPT2	tPBE0	CASPT2	tPBE0	CASPT2	tPBE0
6.022	1.22	0.87	1.24	1.22	0.02	0.35
4.523	1.21	0.86	1.29	1.27	0.09	0.41
3.019	1.20	0.86	1.34	1.32	0.15	0.46
1.512	1.19	0.86	1.36	1.32	0.18	0.46
0.000	1.18	0.85	1.29	1.25	0.11	0.40
-1.512	1.19	0.86	1.36	1.32	0.18	0.46
-3.019	1.20	0.86	1.34	1.32	0.15	0.46
-4.523	1.21	0.86	1.29	1.27	0.09	0.41
-6.022	1.22	0.87	1.24	1.22	0.02	0.35

Table S75: Energy differences in eV between ground state T_0 and first excited state S_1, between the two triplet excited states $T_0 - T_1$, and between the singlet and triplet excited states $S_1 - T_1$ of Ti(o-tol)$_4^{2-}$ complex for the Ti–C symmetric stretching. The equilibrium bond distance is $d = 2.16$ Å.

	$\Delta E_{T_0-S_1}$		$\Delta E_{T_0-T_1}$		$\Delta E_{S_1-T_1}$	
Δd (Å)	CASPT2	tPBE0	CASPT2	tPBE0	CASPT2	tPBE0
0.077	0.74	0.65	0.64	0.64	-0.10	-0.01
0.058	0.70	0.62	0.63	0.63	-0.07	0.01
0.039	0.65	0.60	0.62	0.63	-0.03	0.03
0.019	0.61	0.57	0.62	0.64	0.01	0.06
0.000	0.56	0.55	0.61	0.64	0.05	0.09
-0.019	0.52	0.52	0.61	0.65	0.09	0.13
-0.039	0.47	0.50	0.62	0.66	0.14	0.16
-0.058	0.43	0.47	0.63	0.68	0.20	0.21
-0.077	0.39	0.45	0.65	0.70	0.26	0.26

Table S76: Energy differences in eV between ground state T_0 and first excited state S_1, between the two triplet excited states $T_0 - T_1$, and between the singlet and triplet excited states $S_1 - T_1$ of Ti(o-tol)$_4^{2-}$ complex for the C–Ti–C scissoring mode. The equilibrium bond angle is $\theta = 106.0°$.

	$\Delta E_{T_0-S_1}$		$\Delta E_{T_0-T_1}$		$\Delta E_{S_1-T_1}$	
$\Delta\theta$ (°)	CASPT2	tPBE0	CASPT2	tPBE0	CASPT2	tPBE0
7.000	0.56	0.55	0.61	0.63	0.05	0.09
5.000	0.56	0.55	0.61	0.64	0.05	0.09
2.277	0.56	0.55	0.61	0.64	0.05	0.09
1.708	0.56	0.55	0.61	0.64	0.05	0.09
1.139	0.56	0.55	0.61	0.64	0.05	0.09
0.570	0.56	0.55	0.61	0.64	0.05	0.09
0.000	0.56	0.55	0.61	0.64	0.05	0.09
-0.569	0.56	0.55	0.61	0.64	0.05	0.09
-1.140	0.56	0.55	0.61	0.64	0.05	0.09
-1.710	0.56	0.55	0.61	0.64	0.05	0.09
-2.281	0.56	0.55	0.61	0.64	0.05	0.09
-5.000	0.56	0.55	0.61	0.64	0.05	0.09
-7.000	0.56	0.55	0.61	0.63	0.05	0.09

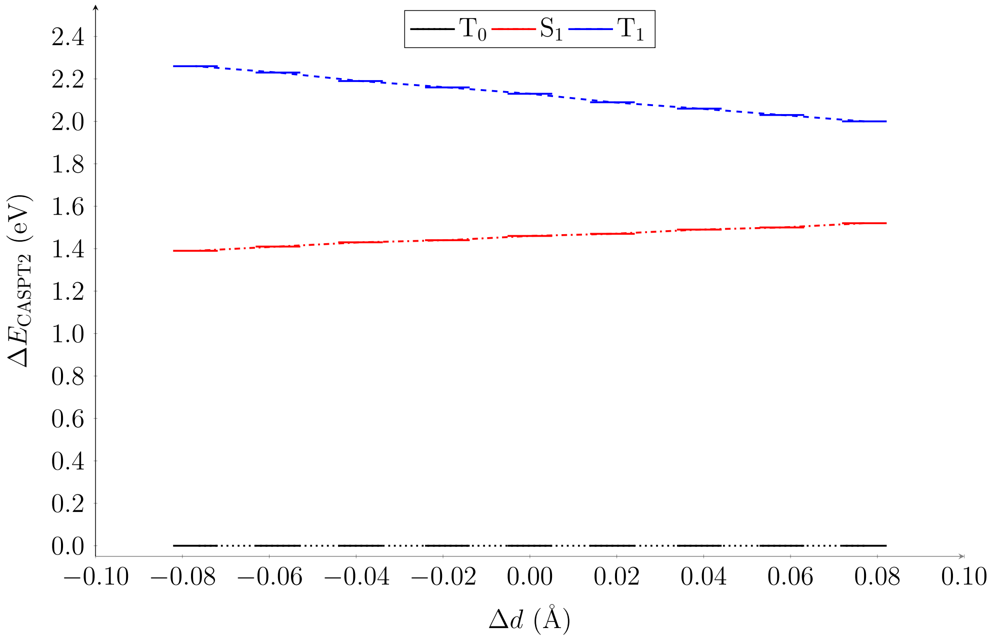

(a) CASPT2 Relative energies

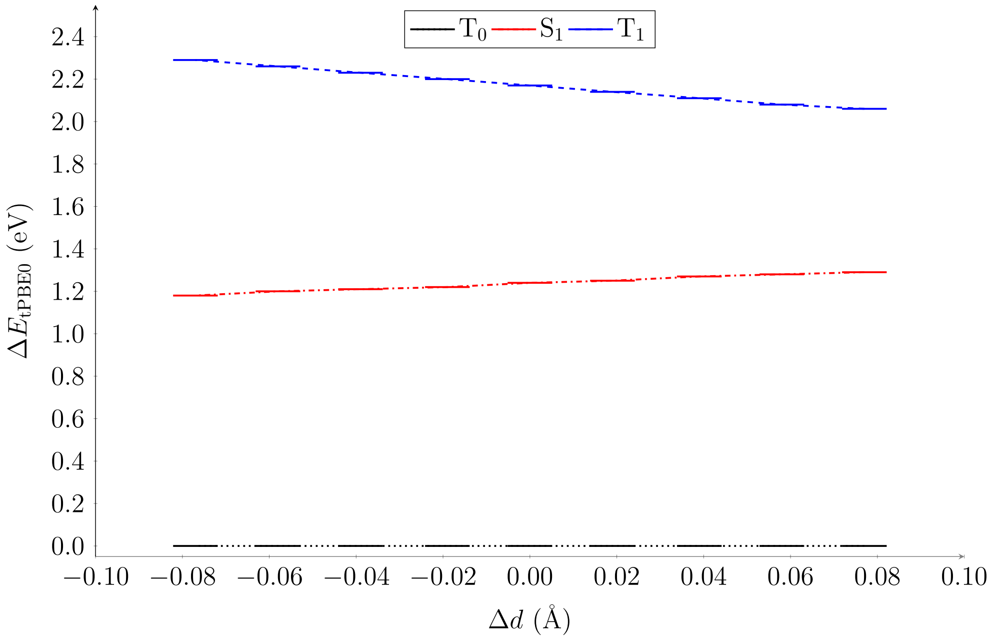

(b) tPBE0 Relative energies

Figure S24: (a) CASPT2 and (b) tPBE0 Relative energies for the lowest-lying three states of Cr(o-tol)$_4$ complex for the Cr–C symmetric stretching normal mode. The equilibrium bond distance is $d = 1.98$ Å.

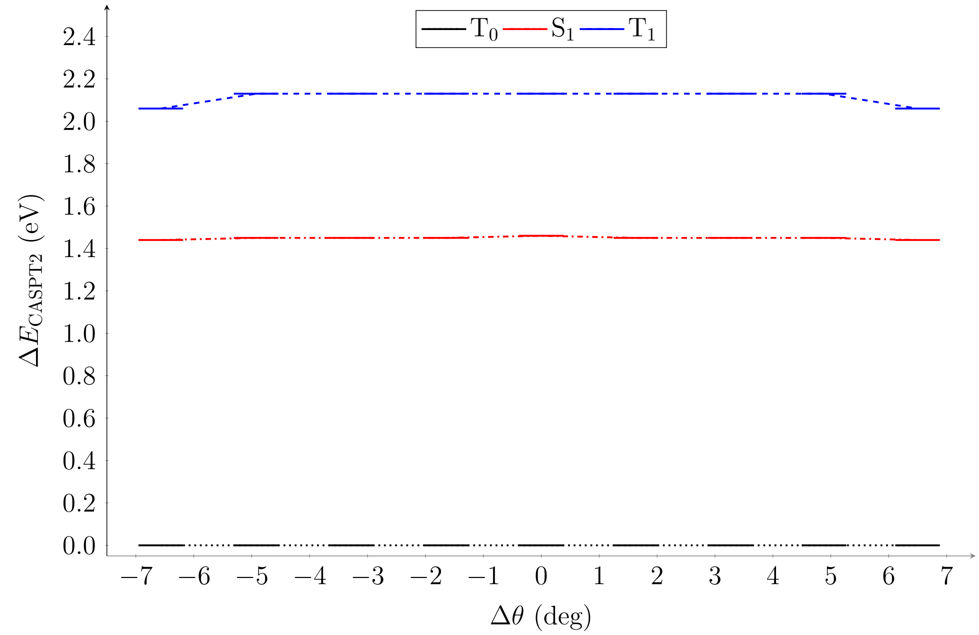

(a) CASPT2 Relative energies

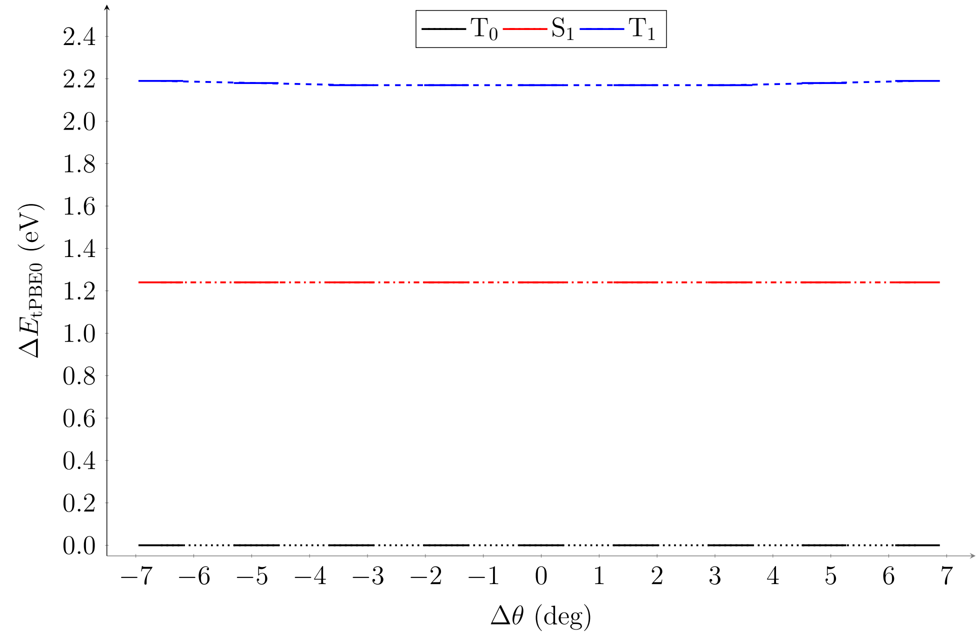

(b) tPBE0 Relative energies

Figure S25: (a) CASPT2 and (b) tPBE0 Relative energies for the lowest-lying three states of Cr(o-tol)$_4$ complex for the C–Cr–C scissoring normal mode. The equilibrium angle is $\theta = 104.9°$.

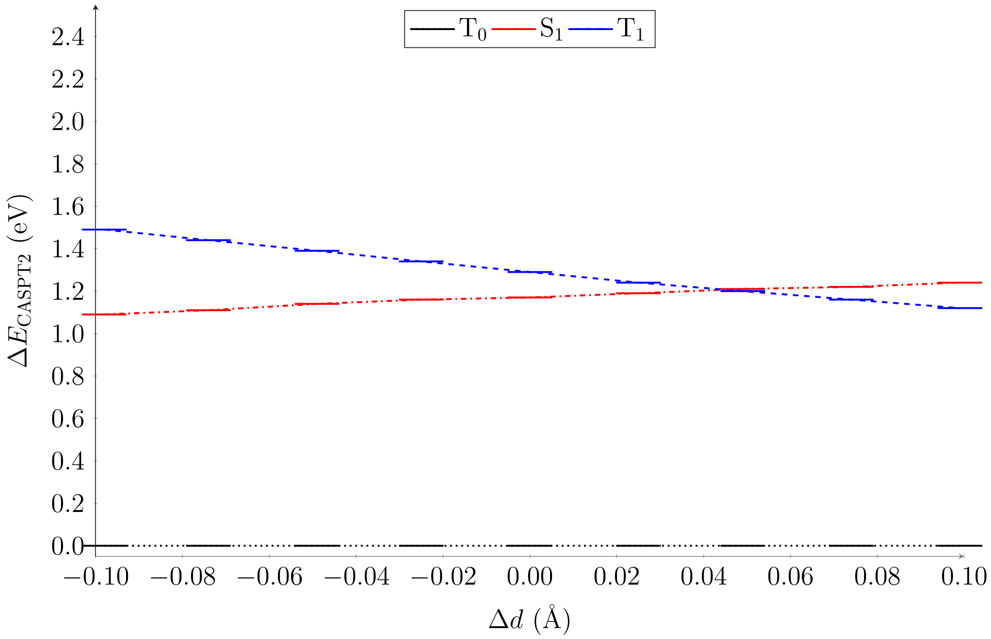

(a) CASPT2 Relative energies

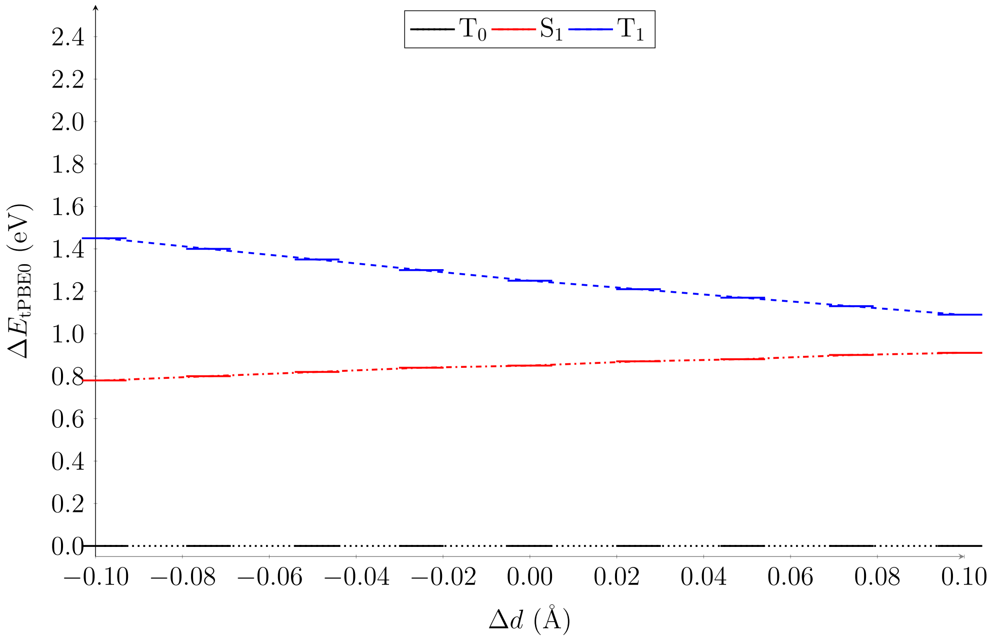

(b) tPBE0 Relative energies

Figure S26: (a) CASPT2 and (b) tPBE0 Relative energies for the lowest-lying three states of $V(o\text{-tol})_4^-$ complex for the V–C symmetric stretching normal mode. The equilibrium bond distance is $d = 2.07$ Å.

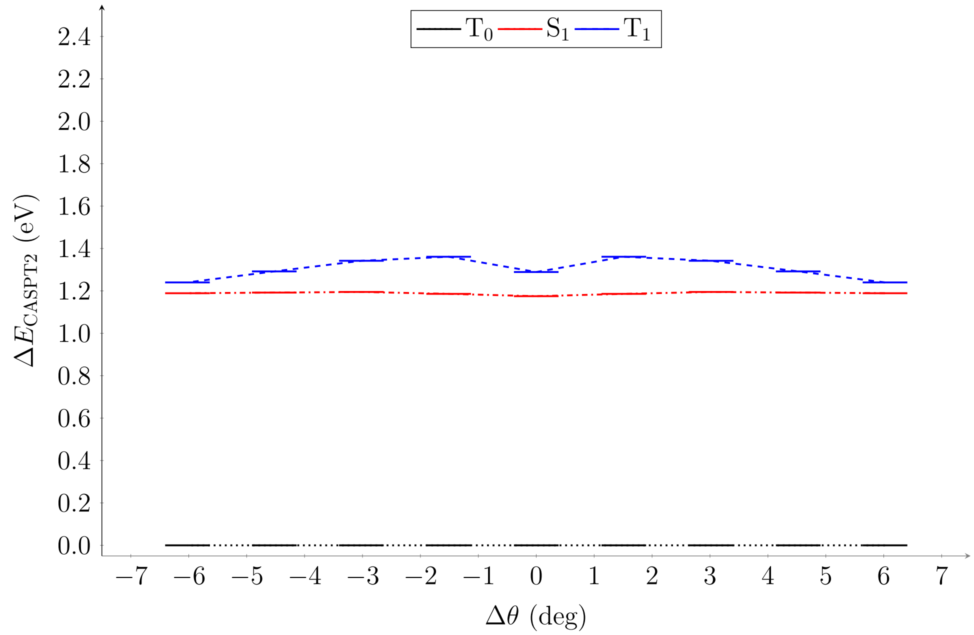

(a) CASPT2 Relative energies

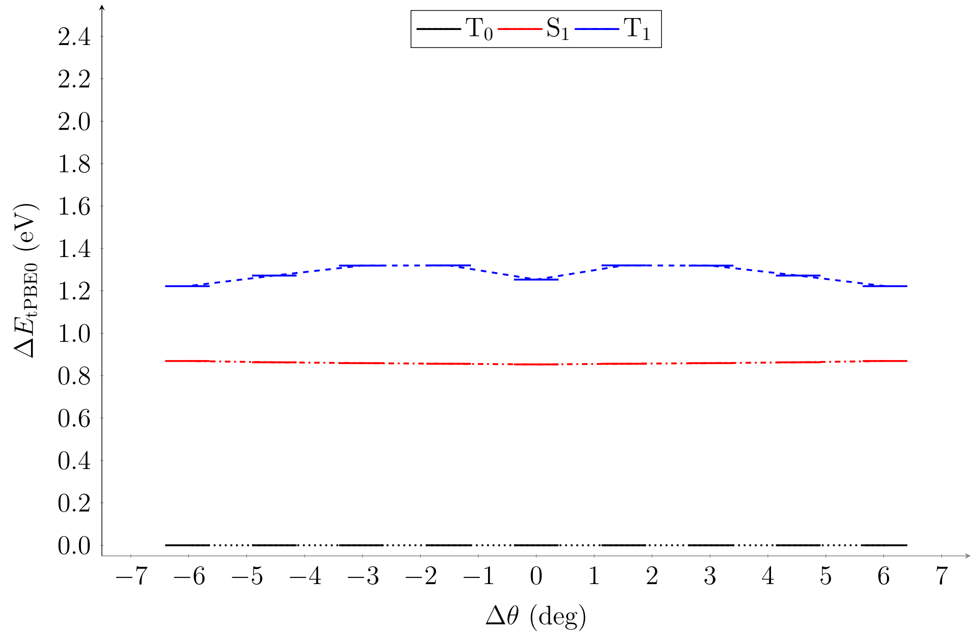

(b) tPBE0 Relative energies

Figure S27: (a) CASPT2 and (b) tPBE0 Relative energies for the lowest-lying three states of $V(o\text{-tol})_4^-$ complex for the C–V–C scissoring normal mode. The equilibrium angle is $\theta = 105.2°$.

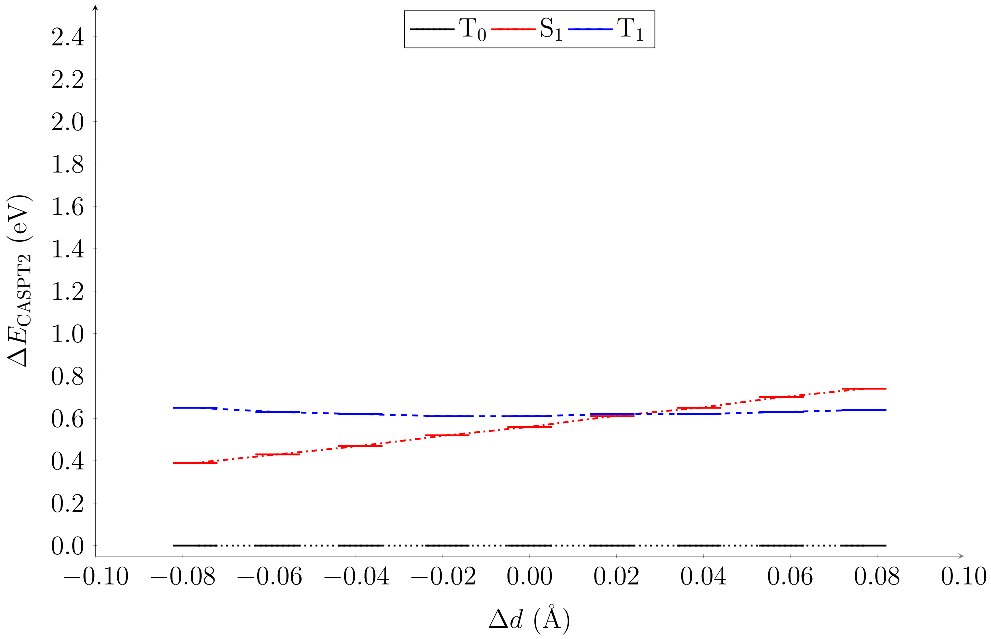

(a) CASPT2 Relative energies

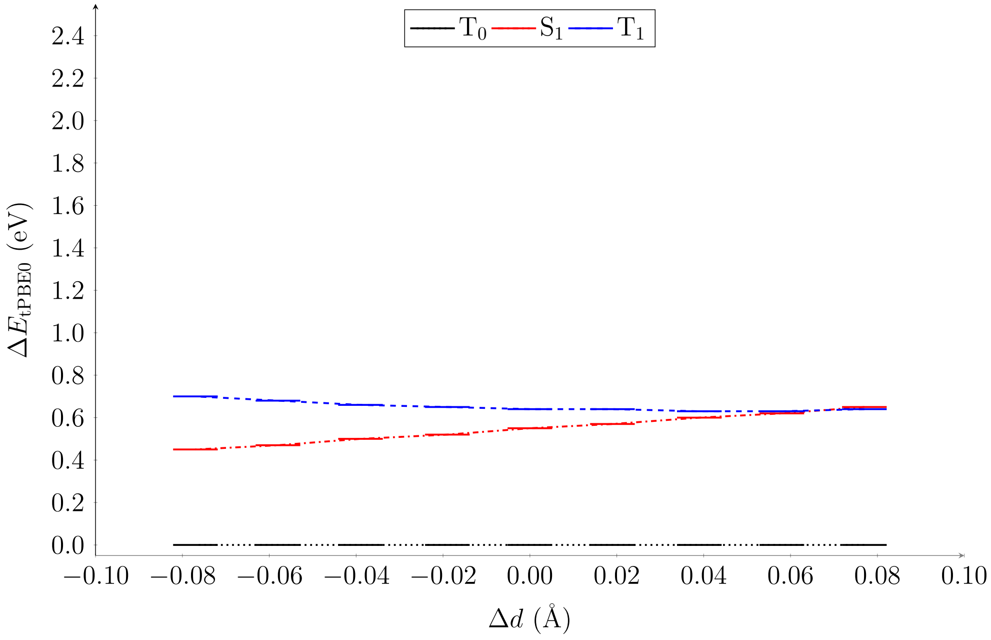

(b) tPBE0 Relative energies

Figure S28: (a) CASPT2 and (b) tPBE0 Relative energies for the lowest-lying three states of $Ti(o\text{-tol})_4^{2-}$ complex for the Ti–C symmetric stretching normal mode. The equilibrium bond distance is $d = 2.16$ Å.

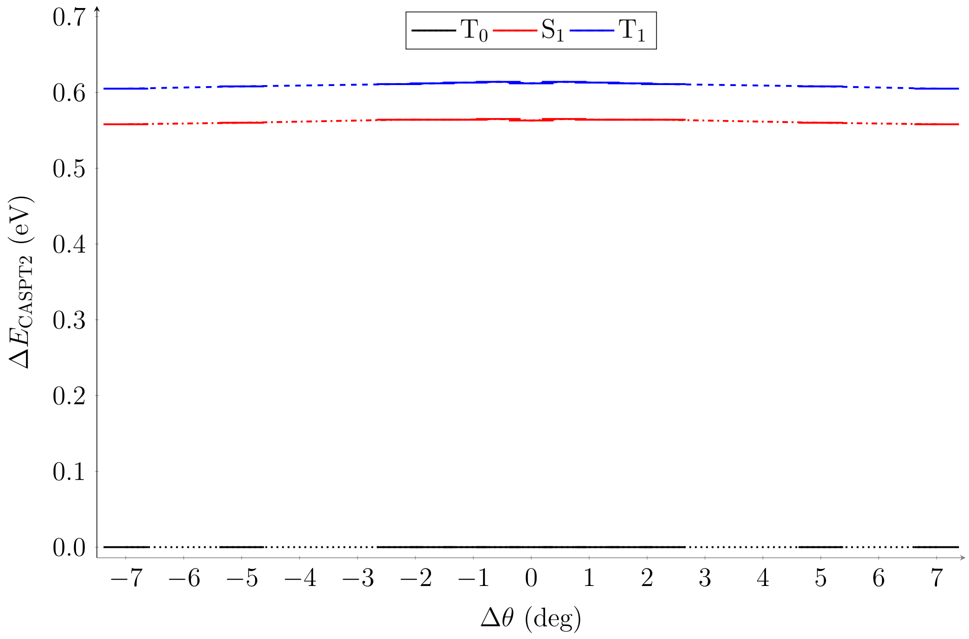

(a) CASPT2 Relative energies

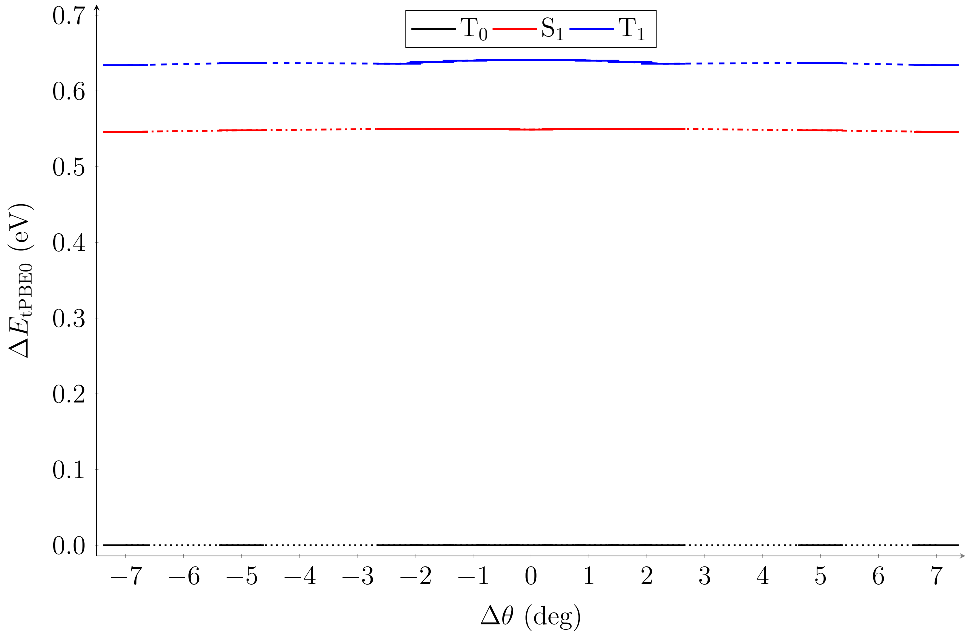

(b) tPBE0 Relative energies

Figure S29: (a) CASPT2 and (b) tPBE0 Relative energies for the lowest-lying three states of Ti(o-tol)$_4^{2-}$ complex for the C–Ti–C scissoring normal mode. The equilibrium angle is $\theta = 106.0°$.

In the subsequent plots, we followed the triplet and singlet manifolds through the scan

coordinate.

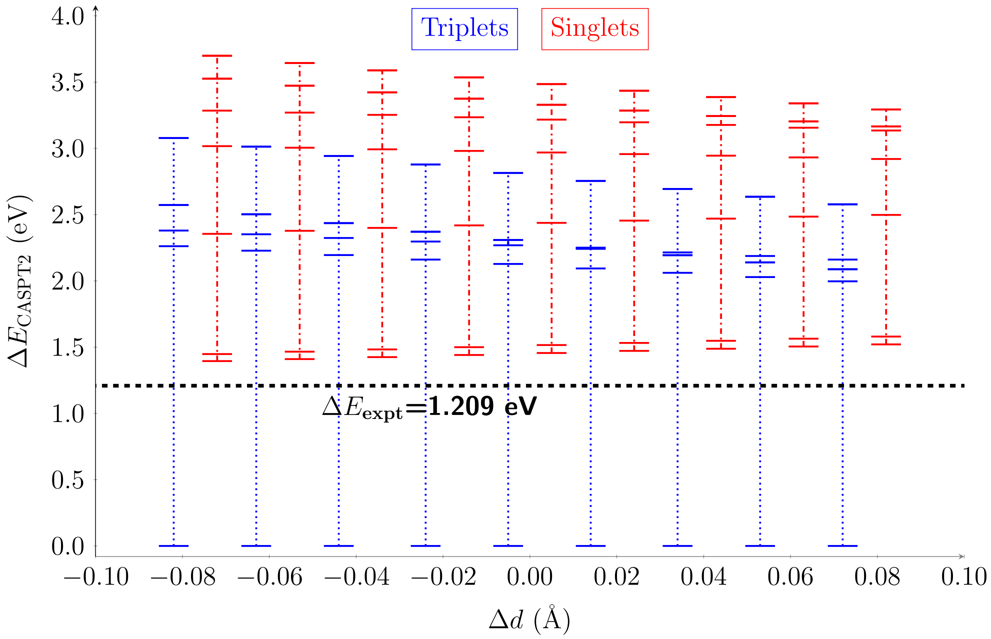

(a) CASPT2 Relative energies

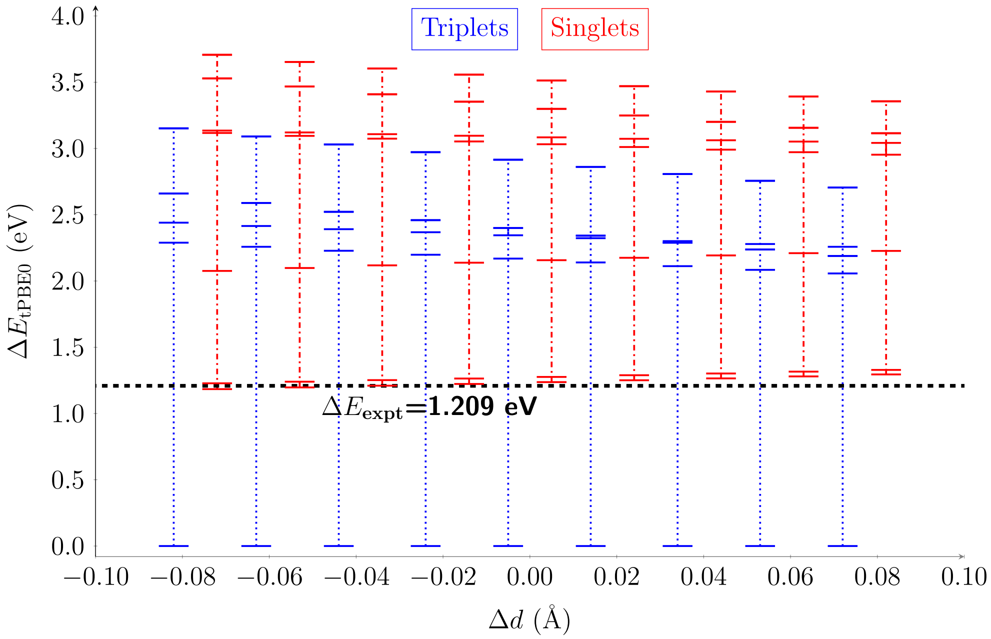

(b) tPBE0 Relative energies

Figure S30: (a) CASPT2 and (b) tPBE0 Relative energies for the lowest-lying 7 triplet and 9 singlet states of $Cr(o\text{-tol})_4$ complex for the Cr–C symmetric stretching normal mode. The equilibrium bond distance is $d = 1.98$ Å.

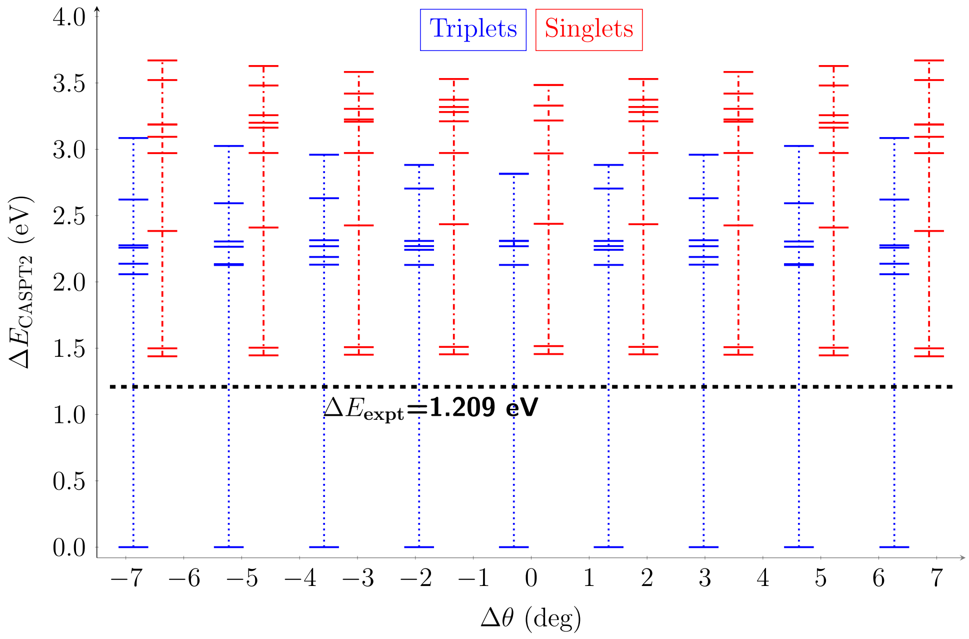

(a) CASPT2 Relative energies

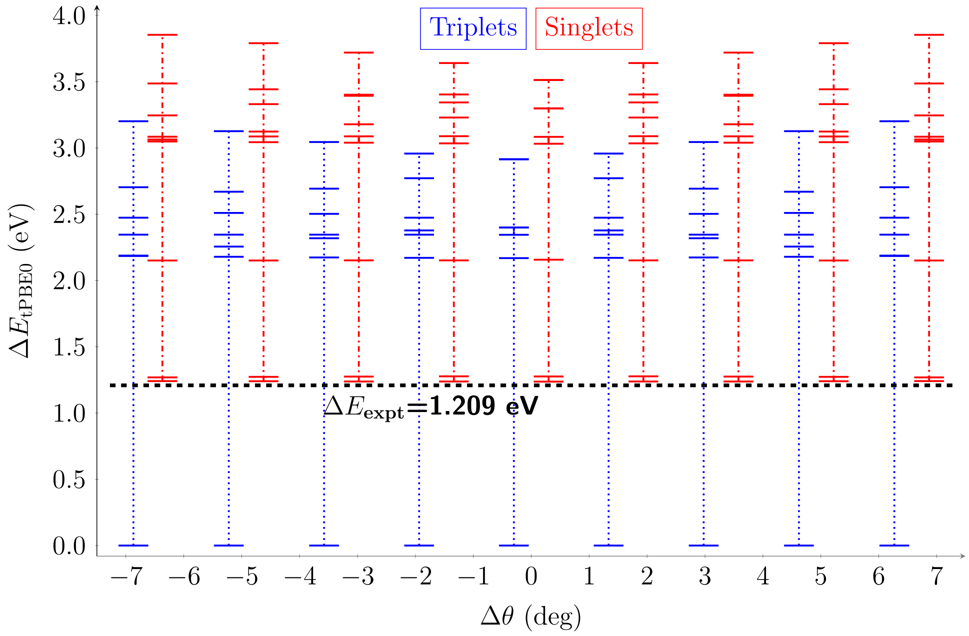

(b) tPBE0 Relative energies

Figure S31: (a) CASPT2 and (b) tPBE0 Relative energies for the lowest-lying 7 triplet and 9 singlet states of Cr(o-tol)$_4$ complex for the C–Cr–C scissoring normal mode. The equilibrium angle is $\theta = 104.9°$.

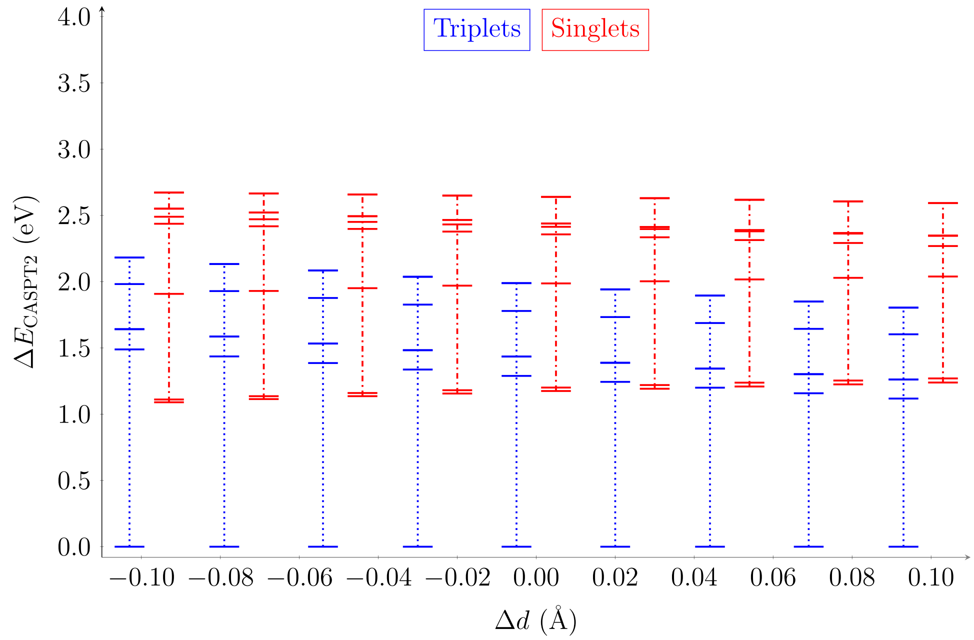

(a) CASPT2 Relative energies

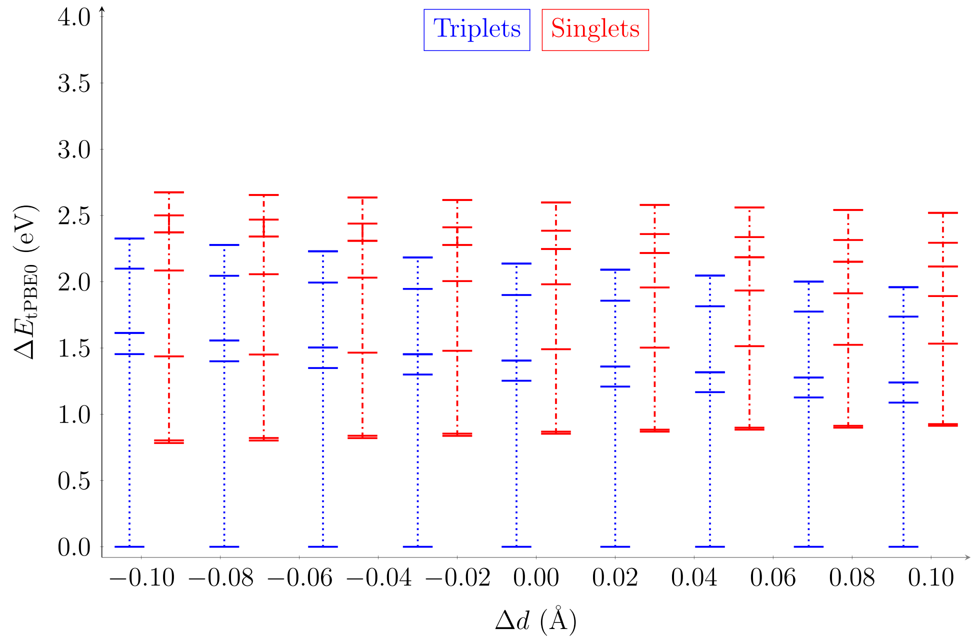

(b) tPBE0 Relative energies

Figure S32: (a) CASPT2 and (b) tPBE0 Relative energies for the lowest-lying 7 triplet and 9 singlet states of $V(o\text{-tol})_4^-$ complex for the V–C symmetric stretching normal mode. The equilibrium bond distance is $d = 2.07$ Å.

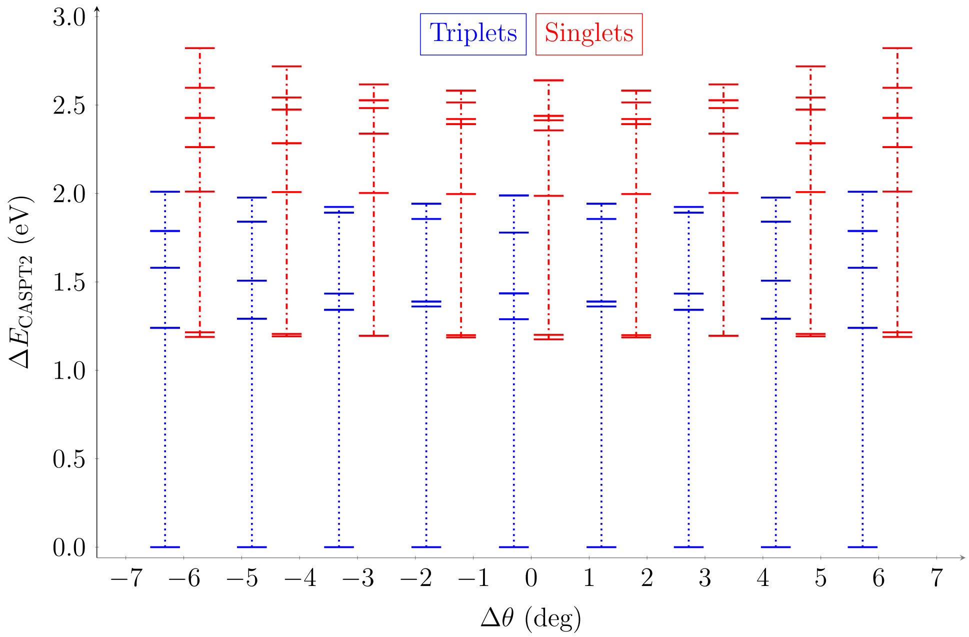

(a) CASPT2 Relative energies

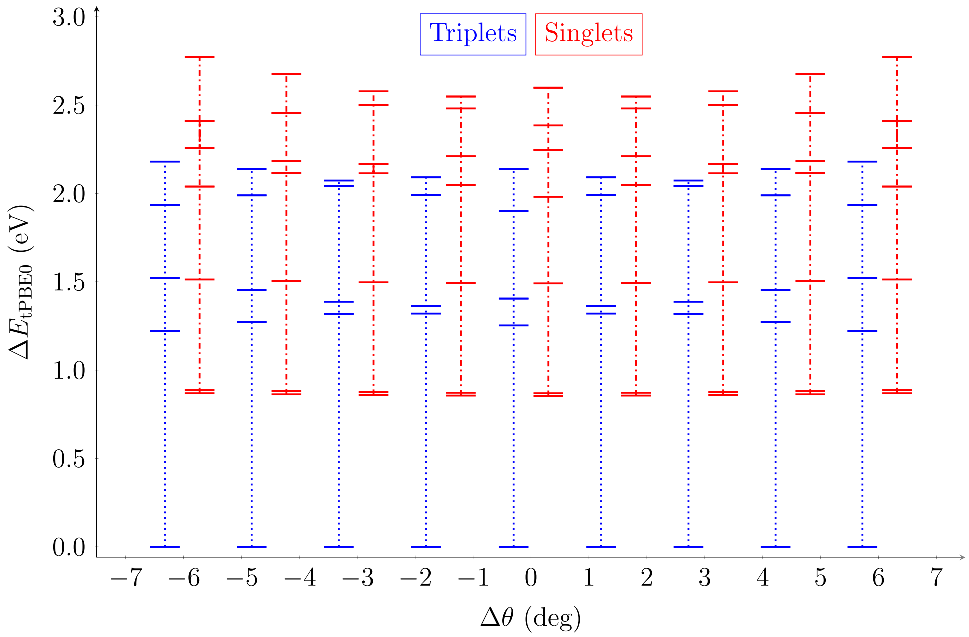

(b) tPBE0 Relative energies

Figure S33: (a) CASPT2 and (b) tPBE0 Relative energies for the lowest-lying 7 triplet and 9 singlet states of $V(o\text{-tol})_4^-$ complex for the C–V–C scissoring normal mode. The equilibrium angle is $\theta = 105.2°$.

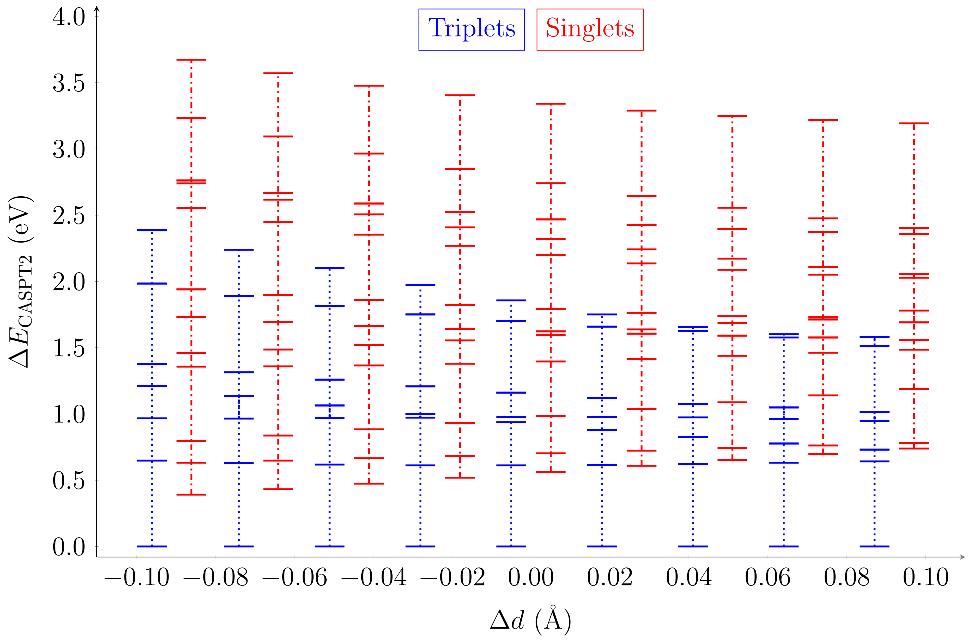

(a) CASPT2 Relative energies

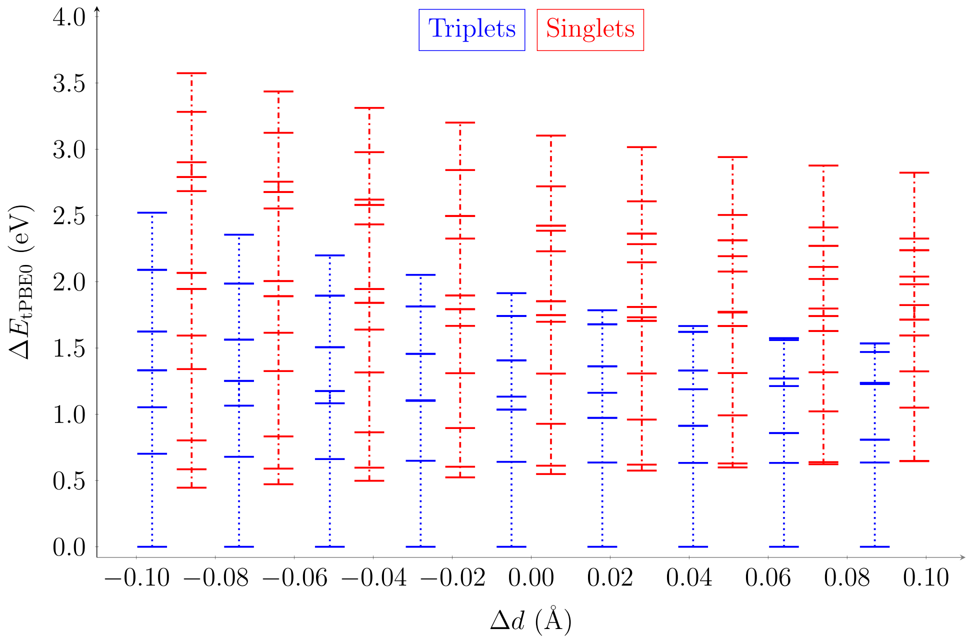

(b) tPBE0 Relative energies

Figure S34: (a) CASPT2 and (b) tPBE0 Relative energies for the lowest-lying 10 triplet and 15 singlet states of $\text{Ti}(o\text{-tol})_4^{2-}$ complex for the Ti–C symmetric stretching normal mode. The equilibrium bond distance is $d = 2.16$ Å.

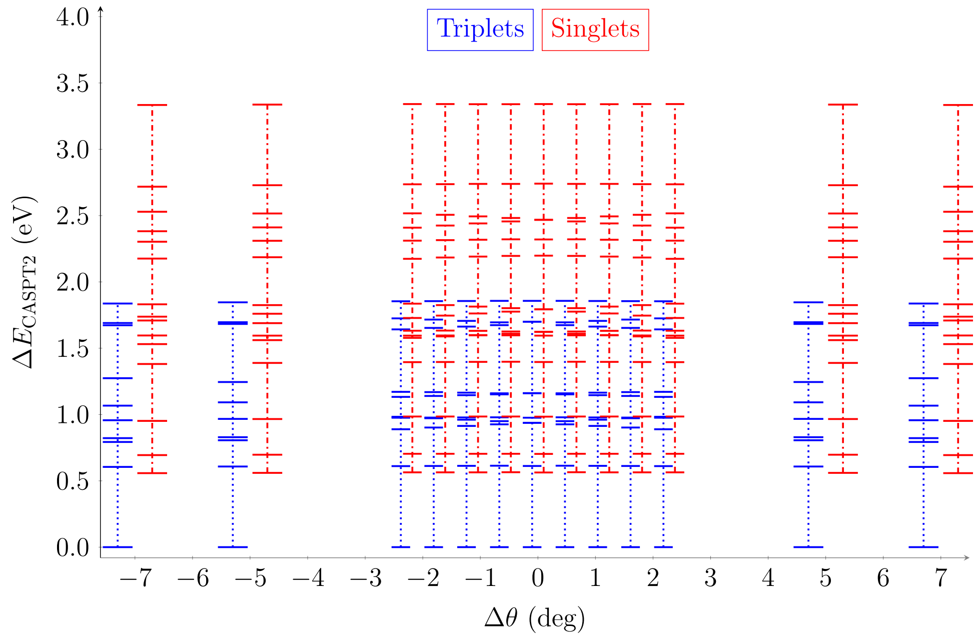

(a) CASPT2 Relative energies

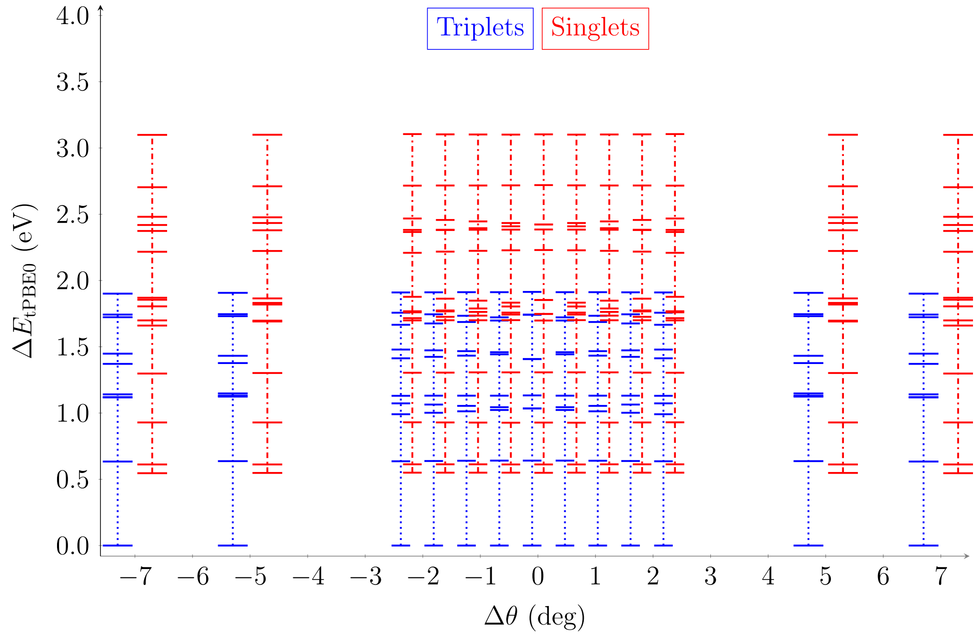

(b) tPBE0 Relative energies

Figure S35: (a) CASPT2 and (b) tPBE0 Relative energies for the lowest-lying 10 triplet and 15 singlet states of Ti(o-tol)$_4^{2-}$ complex for the C–Ti–C scissoring normal mode. The equilibrium angle is $\theta = 106.0°$.

The ZFS axial parameter $|D|$ was computed using the distorted structures obtained along

the normal modes for Cr(o-tol)$_4$, V(o-tol)$_4^-$, and Ti(o-tol)$_4^{2-}$ complexes. In Figures S36-S41 are displayed the $|D|$ values computed with CASPT2 and tPBE0.

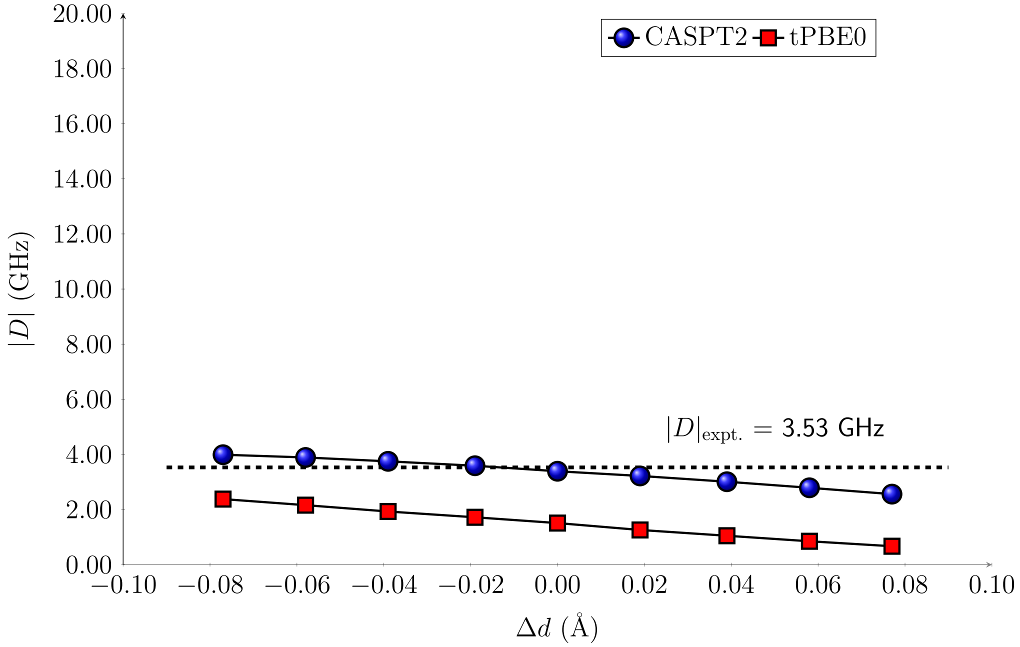

Figure S36: CASPT2 and tPBE0 axial parameter ($|D|$) of Cr(o-tol)$_4$ complex for the Cr–C symmetric stretching normal mode. The equilibrium bond distance is $d = 1.98$ Å.

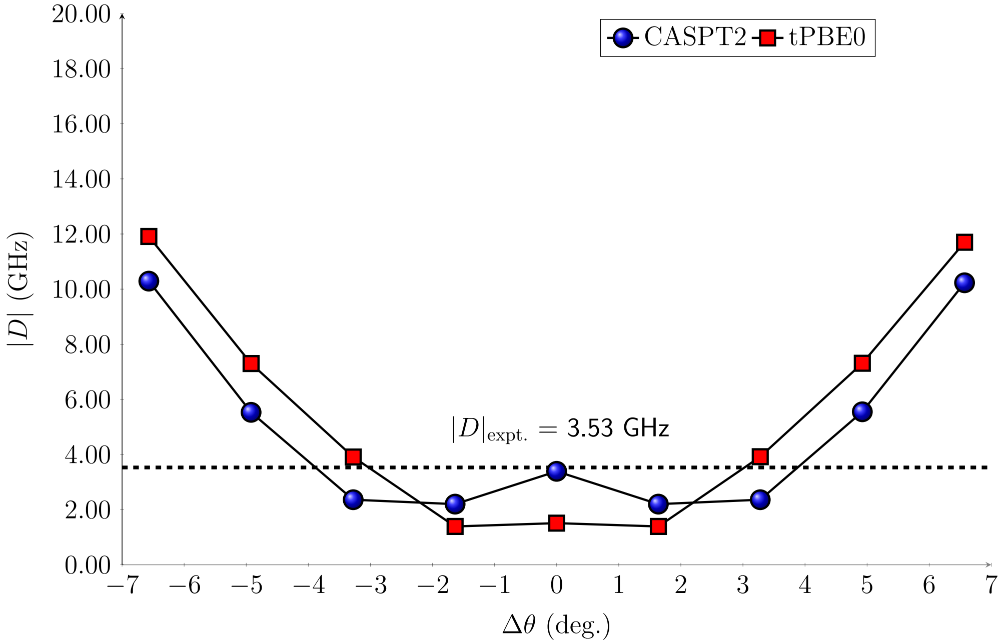

Figure S37: CASPT2 and tPBE0 axial parameter ($|D|$) of Cr(o-tol)$_4$ complex for the C–Cr–C scissoring normal mode. The equilibrium bond angle is $\theta = 104.9°$.

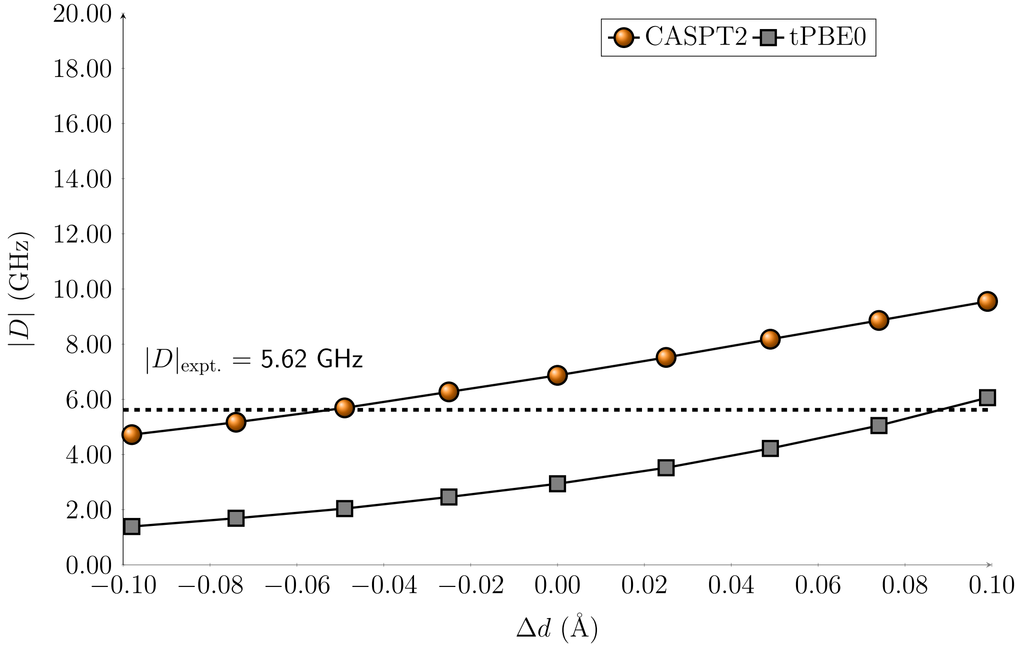

Figure S38: CASPT2 and tPBE0 axial parameter ($|D|$) of $V(o\text{-tol})_4^-$ complex for the V–C symmetric stretching normal mode. The equilibrium bond distance is $d = 2.07$ Å.

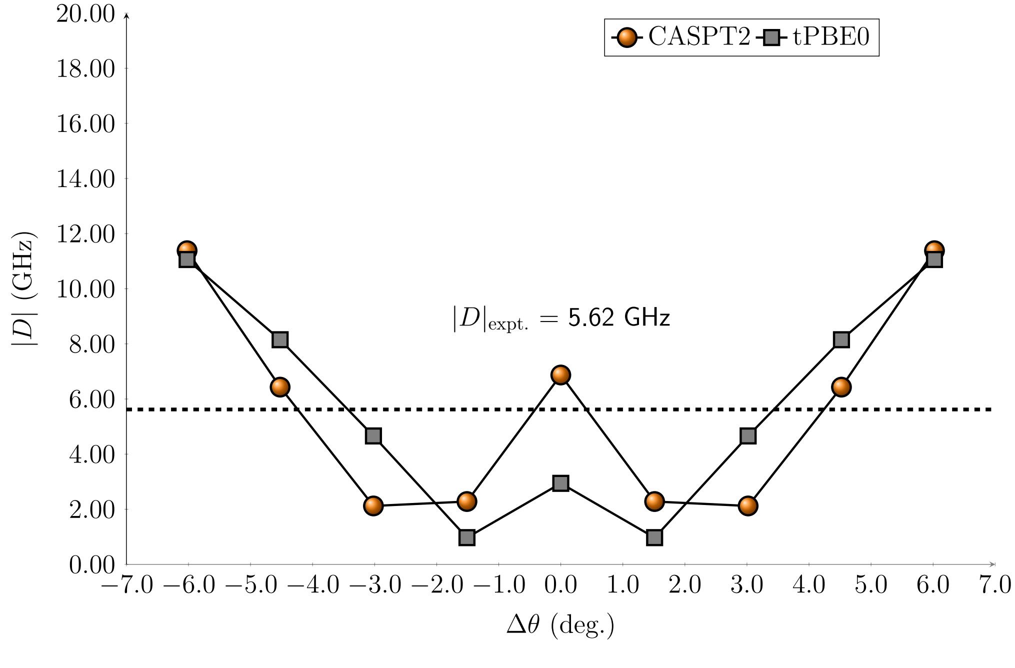

Figure S39: Zero-field splitting parameters computed with CASPT2 and tPBE0 methods for the $V(o\text{-tol})_4^-$ complex considering the C–V–C scissoring normal mode. The equilibrium bond angle is $\theta = 105.2°$.

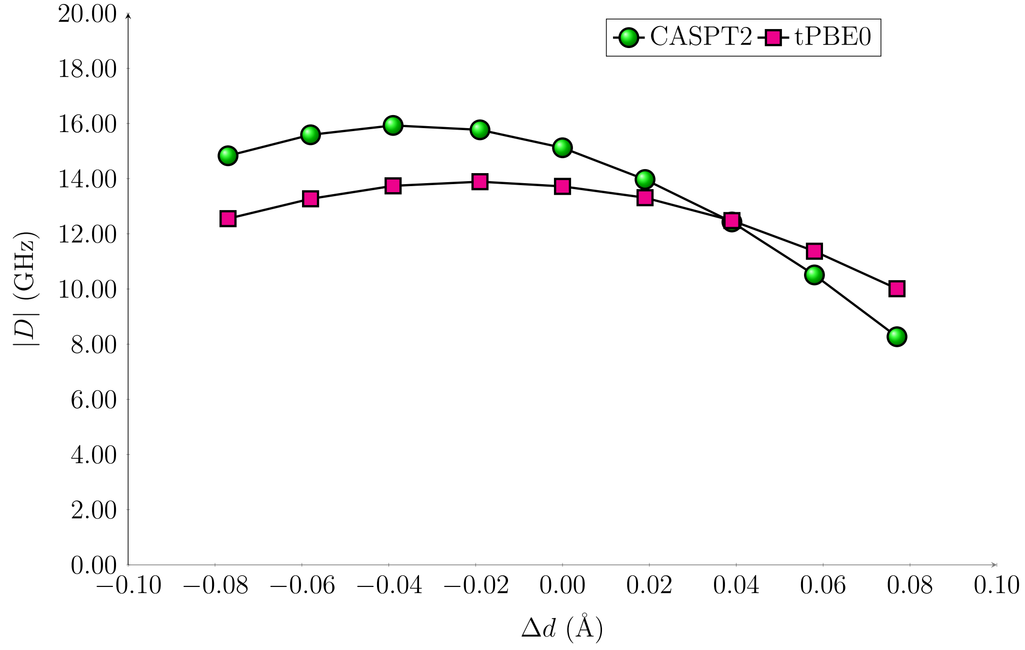

Figure S40: CASPT2 and tPBE0 axial parameter ($|D|$) of $\mathrm{Ti}(o\text{-tol})_4^{2-}$ complex for the Ti–C symmetric stretching normal mode. The equilibrium bond distance is $d = 2.16$ Å.

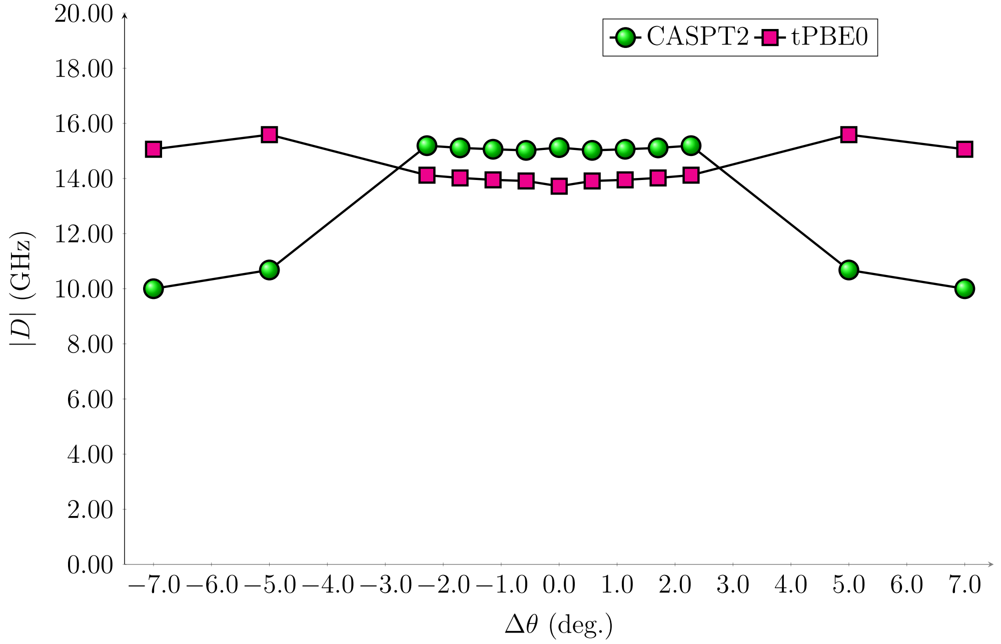

Figure S41: CASPT2 and tPBE0 axial parameter ($|D|$) of $\mathrm{Ti}(o\text{-tol})_4^{2-}$ complex for the C–Ti–C scissoring normal mode. The equilibrium bond angle is $\theta = 106.0°$.

Table S77: Zero-field splitting parameter $|D|$ in GHz computed with CASSCF, CASPT2, tPBE, and tPBE0 methods for the $Cr(o\text{-tol})_4$ complex considering the Cr–C stretching mode. The equilibrium bond distance is $d = 1.98$ Å.

Δd (Å)	CASSCF	CASPT2	tPBE	tPBE0
0.077	3.22	2.56	0.67	0.67
0.058	3.44	2.79	0.51	0.85
0.039	3.67	3.01	0.33	1.05
0.019	3.89	3.22	0.12	1.26
0.000	4.09	3.39	0.15	1.51
-0.019	4.28	3.59	0.37	1.72
-0.039	4.45	3.75	0.60	1.93
-0.058	4.59	3.89	0.88	2.16
-0.077	4.71	3.99	1.15	2.38

Table S78: Zero-field splitting parameter $|D|$ in GHz computed with CASSCF, CASPT2, tPBE, and tPBE0 methods for the $Cr(o\text{-tol})_4$ complex considering the C–Cr–C scissoring mode. The equilibrium angle is $\theta = 104.9°$.

$\Delta\theta$ (°)	CASSCF	CASPT2	tPBE	tPBE0
6.495	8.20	10.29	0.00	11.91
4.881	4.74	5.53	8.60	7.30
3.259	1.84	2.36	4.98	3.91
1.632	3.39	2.20	1.76	1.39
0.001	4.09	3.39	0.15	1.51
-1.638	3.39	2.20	1.76	1.39
-3.279	1.86	2.36	4.98	3.92
-4.923	4.75	5.55	8.60	7.31
-6.570	8.20	10.23	14.23	11.70

Table S79: Zero-field splitting parameter $|D|$ in GHz computed with CASSCF, CASPT2, tPBE, and tPBE0 methods for the $V(o\text{-tol})_4^-$ complex considering the V–C stretching mode. The equilibrium bond distance is $d = 2.07$ Å.

Δd (Å)	CASSCF	CASPT2	tPBE	tPBE0
-0.098	4.52	4.72	0.73	1.39
-0.074	5.05	5.17	0.56	1.69
-0.049	5.63	5.69	0.34	2.04
-0.025	6.27	6.27	0.06	2.46
0.000	6.96	6.87	0.31	2.94
0.025	7.71	7.52	0.79	3.52
0.049	8.52	8.18	1.43	4.22
0.074	9.40	8.86	2.25	5.05
0.099	10.34	9.55	3.31	6.06

Table S80: Zero-field splitting parameter $|D|$ in GHz computed with CASSCF, CASPT2, tPBE, and tPBE0 methods for the $V(o\text{-tol})_4^-$ complex considering the C–V–C scissoring mode. The equilibrium angle is $\theta = 105.2°$.

$\Delta\theta$ (°)	CASSCF	CASPT2	tPBE	tPBE0
6.022	13.31	11.38	10.01	11.06
4.523	8.32	6.43	8.33	8.15
3.019	3.30	2.12	5.73	4.66
1.512	1.77	2.28	2.84	0.97
0.000	6.96	6.87	0.31	2.94
-1.512	1.77	2.28	2.84	0.97
-3.019	3.30	2.12	5.73	4.66
-4.523	8.32	6.43	8.33	8.15
-6.022	13.31	11.38	10.01	11.06

Table S81: Zero-field splitting parameter $|D|$ in GHz computed with CASSCF, CASPT2, tPBE, and tPBE0 methods for the $Ti(o\text{-tol})_4^{2-}$ complex considering the Ti–C stretching mode. The equilibrium bond distance is $d = 2.16$ Å.

Δd (Å)	CASSCF	CASPT2	tPBE	tPBE0
0.077	15.66	8.27	7.42	10.01
0.058	16.28	10.51	9.09	11.37
0.039	16.78	12.43	10.46	12.48
0.019	17.11	13.97	11.51	13.31
0.000	17.21	15.12	12.06	13.72
-0.019	17.07	15.77	12.38	13.89
-0.039	16.66	15.93	12.37	13.74
-0.058	16.01	15.59	12.00	13.27
-0.077	15.12	14.83	11.38	12.55

Table S82: Zero-field splitting parameter $|D|$ in GHz computed with CASSCF, CASPT2, tPBE, and tPBE0 methods for the Ti(o-tol)$_4^{2-}$ complex considering the C–Ti–C scissoring mode. The equilibrium angle is $\theta = 106.0°$.

$\Delta\theta$ (°)	CASSCF	CASPT2	tPBE	tPBE0
7.000	16.49	10.00	13.93	15.06
5.000	16.85	10.68	14.60	15.59
-2.281	17.44	15.19	12.58	14.12
-1.710	17.34	15.11	12.48	14.02
-1.140	17.27	15.06	12.41	13.95
-0.570	17.23	15.02	12.37	13.91
0.001	17.21	15.12	12.06	13.72
0.570	17.23	15.02	12.37	13.91
1.139	17.27	15.06	12.41	13.95
1.708	17.34	15.11	12.48	14.02
2.277	17.44	15.19	12.57	14.12
5.000	16.85	10.68	14.60	15.59
7.000	16.49	10.00	13.93	15.06

References

(1) Bayliss, S. L.; Laorenza, D. W.; Mintum, P. J.; Kovos, B. D.; Freedman, D. E.; Awschalom, D. D. Optically addressable molecular spins for quantum information processing. Science **2020**, 370, 1309–1312.

(2) Laorenza, D. W.; Mullin, K. R.; Weiss, L. R.; Bayliss, S. L.; Deb, P.; Awschalom, D. D.; Rondinelli, J. M.; Freedman, D. E. Coherent spin-control of S = 1 vanadium and molybdenum complexes. Chem. Sci. **2024**, 15, 14016–14026.

(3) Gattesch, D.; Sessoli, R.; Villain, J. Molecular Nanomagnets: Mesoscopic Physics and Nanotechnology; Oxford University Press, 2006.

(4) Pederson, M. R.; Khanna, S. N. Magnetic anisotropy barrier for spin tunneling in $Mn_{12}O_{12}$ molecules. Phys. Rev. B **1999**, 60, 9566–9572.

(5) Reviakine, R.; Arbuznikov, A. V.; Tremblay, J.-C.; Remenyi, C.; Malkina, O. L.; Malkin, V. G.; Kaupp, M. Calculation of zero-field splitting parameters: Comparison

of a two-component noncolinear spin-density-functional method and a one-component perturbational approach. The Journal of Chemical Physics **2006**, 125, 054110.

(6) Chibotaru, L.; Ungur, L.; Soncini, A. The Origin of Nonmagnetic Kramers Doublets in the Ground State of Dysprosium Triangles: Evidence for a Toroidal Magnetic Moment. Angew. Chem. Int. Ed. **2008**, 47, 4126–4129.

(7) Chibotaru, L. F.; Ungur, L.; Aronica, C.; Elmoll, H.; Pilet, G.; Luneau, D. Structure, Magnetism, and Theoretical Study of a Mixed-Valence CoII3CoIII4 Heptanuclear Wheel: Lack of SMM Behavior despite Negative Magnetic Anisotropy. J. Am. Chem. Soc **2008**, 130, 12445–12455.

(8) Chibotaru, L. F.; Ungur, L. Ab initio calculation of anisotropic magnetic properties of complexes. I. Unique definition of pseudospin Hamiltonians and their derivation. J. Chem. Phys. **2012**, 137, 064112.

(9) Sauza-de la Vega, A.; Pandharkar, R.; Stroscio, G.; Sarkar, A.; Truhlar, D.; Gagliardi, L. Multiconfiguration Pair-Density Functional Theory for Chromium(IV) Molecular Qubits. JACS Au **2022**, 2, 2029–2037.

(10) Roos, B. O. The complete active space self-consistent field method and its applications in electronic structure calculations. Adv. Chem. Phys. **1987**, 69, 399–445.

(11) Andersson, K.; Malmqvist, P.; Roos, B. O. Second-order Perturbation Theory with a Complete Active Space Self-consistent Field Reference Function. J. Chem. Phys. **1992**, 96, 1218–1226.

(12) Pulay, P. A perspective on the CASPT2 method. Int. J. Quantum Chem. **2011**, 111, 3273–3279.

(13) Li Manni, G.; Carlson, R. K.; Luo, S.; Ma, D.; Olsen, J.; Truhlar, D. G.; Gagliardi, L.

Multiconfiguration Pair-Density Functional Theory. J. Chem. Theory Comput. **2014**, 10, 3669–3680.

(14) Pandharkar, R.; Hermes, M. R.; Truhlar, D. G.; Gagliardi, L. A New Mixing of Nonlocal Exchange and Nonlocal Correlation with Multiconfiguration Pair-Density Functional Theory. J. Phys. Chem. Lett. **2020**, 11, 10158–10163.

(15) Zhou, C.; Wu, D.; Gagliardi, L.; Truhlar, D. G. Calculation of the Zeeman Effect for Transition-Metal Complexes by Multiconfiguration Pair-Density Functional Theory. J. Chem. Theory Comput. **2021**, 17, 5050 –5063.

(16) Wu, D.; Zhou, C.; Bao, J. J.; Gagliardi, L.; Truhlar, D. G. Zero-Field Splitting Calculations by Multiconfiguration Pair-Density Functional Theory. J. Chem. Theory Comput. **2022**, 18, 2199–2207.

(17) Goh, T.; Pandharkar, R.; Gagliardi, L. Multireference Study of Optically Addressable Vanadium-Based Molecular Qubit Candidates. J. Phys. Chem. A **2022**, 126, 6329–6335.

(18) Hertler, P. R.; Sauza-de la Vega, A.; Darù, A.; Sarkar, A.; Lewis, R. A.; Wu, G.; Gagliardi, L.; Hayton, T. W. A homoleptic Fe(IV) ketimide complex with a low-lying excited state. Chem. Sci. **2024**, 15, 16559–16566.

(19) Andrews, D. L.; Scholes, G. D.; Wiederrecht, G. P. Comprehensive Nanoscience And Technology; Elsevier, 2011.

(20) Koseki, S.; Matsunaga, N.; Asada, T.; Schmidt, M. W.; Gordon, M. S. Spin–Orbit Coupling Constants in Atoms and Ions of Transition Elements: Comparison of Effective Core Potentials, Model Core Potentials, and All-Electron Methods. J. Phys. Chem. A **2019**, 123, 2325–2339.
